# Changes in the distribution of elements in the liver and various brain regions in suicides from southeastern Poland

**DOI:** 10.1038/s41598-025-03283-2

**Published:** 2025-05-29

**Authors:** Jacek Baj, Alicja Forma, Kaja Karakuła, Wojciech Flieger, Michał Flieger, Beata Kowalska, Iwona Kamińska, Dariusz Majerek, Grzegorz Teresiński, Ryszard Maciejewski, Elżbieta Radzikowska-Büchner, Jolanta Flieger

**Affiliations:** 1 Department of Correct, Clinical and Imaging Anatomy, Medical University of Lublin, ul. Jaczewskiego 4, Lublin, 20-090 Poland; 2 Department of Forensic Medicine, Medical University of Lublin, ul. Jaczewskiego 8b, Lublin, 20-090 Poland; 3 Department of Water Supply and Wastewater Disposal, Lublin University of Technology, Nadbystrzycka 40B, Lublin, 20-618 Poland; 4 Environmental Analysis Laboratory, Lublin University of Technology, Nadbystrzycka 40B, Lublin, 20-618 Poland; 5 Department of Applied Mathematics, Lublin University of Technology, Nadbystrzycka 38D, Lublin, 20-618 Poland; 6 Present Address: Institute of Health Sciences, John Paul II Catholic University of Lublin, Konstantynów 1 H, Lublin, 20-708 Poland; 7 Department of Plastic, Reconstructive and Hand Surgery, National Medical Institute of the Ministry of the Interior and Administration, Wołoska 137, Warsaw, 02-507 Poland; 8 Department of Analytical Chemistry, Medical University of Lublin, Chodźki 4a (Collegium Pharmaceuticum), Lublin, 20-093 Poland

**Keywords:** Suicide, Mental health, Brain, Liver, Trace elements, Heavy metals, Natural hazards, Health care, Risk factors

## Abstract

The anthropogenic environment and diet are sources of chemical elements, both essential and toxic. Many of them play a key role in the proper functioning of the brain by controlling oxidative metabolism, neurotransmission and the production of neurotransmitters. Disturbances in element homeostasis are associated with the development of depression and many neurodegenerative diseases. The study examined the content of several elements in liver and brain samples taken from autopsies of residents of south-eastern Poland (*n* = 40). In order to determine the differences in the elemental composition of tissues collected from suicide victims compared to the control group who died from another cause, the Mann-Whitney U test was performed. There was a significant difference in the correlation patterns between the two groups for both brain (χ² = 1490.19, df = 1378, *p* < 7e-122, z = 3.72) and liver χ² = 2998.7, df = 1378, *p* < 0.018, z = 126.71). Two-way non-parametric Spearman rank correlation matrices showed different numbers and strengths of correlations for most brain regions in the compared groups, especially for the head of the caudate nucleus (Latin: *caput nuclei caudati*), superior longitudinal fasciculus (Latin: *fasciculus longitudinalis superior cerebri*), and hippocampus (Latin: *hippocampus*). Differences between groups reached statistical significance in the case of higher lanthanide content in the liver of suicides and lower Ca content in the basal ganglia (head of the caudate nucleus, nucleus accumbens septum), dorsal thalamus and insula. Brain samples from different regions of suicide victims were characterized by higher levels of, among others: Ag, P, Fe and Cu. Our findings suggest clear differences in trace metal content and inter-correlations in the brain and liver tissues of suicide victims compared to controls.

**Supplementary Information:**

The online version contains supplementary material available at 10.1038/s41598-025-03283-2.

## Introduction

According to 2020 WHO data, approximately 800,000 people commit suicide every year^1^. It is disturbing that in the age group 19–25, suicide is the second leading cause of death in the USA. The annual suicide rate is 11.4 per 100,000 people. It should not be forgotten that for every suicide death, there are twenty or more suicide attempts^2^. It has long been known that people who commit suicide often suffer from depressive disorders^3^.

The biopsychosocial concept of depression assumes that mental conditions are based on a biological basis^4^. It has been shown that one of the elements of the pathogenesis of depression is impaired neurotransmission^5^. Significantly higher risk of developing depression was observed in individuals with non-alcoholic fatty liver disease (NAFLD) and insulin resistance^6,7^.Significant evidence links depression to the gut microbiota^8^. The risk of depression increases in patients treated with β-blockers^9^.

In recent years, more and more attention has been paid to the relationship between exposure to toxic metals and mental disorders in humans^10^. Accumulation of toxic metals or exposure to Pb, Cd, Mn, Hg, As, and Sr, as well as Sb, Be, Ce, Tl, Sn, W, and U, have been associated with depression^11^. The study by Figgs et al.^12^ showed that occupational exposure to U and Be was related to the risk of suicide. The study was conducted among 6,820 employees of the nuclear industry. The authors conclude that the risk of suicide is associated with exposure to U, but only exposure to Be resulted in increased hazard ratios (HRs). The study by Fu et al.^13^, which included 4212 participants, showed that elevated urinary levels of tin (Sn) and antimony (Sb) are associated with an increased risk of depression. Recent research by Mergler et al.^14^ conducted among the indigenous community of Grassy Narrows in Ontario (Canada), proves that chronic environmental exposure to Hg is associated with a high suicide rate, especially among youth. The study analyzed the number of suicide attempts in a group of 162 children divided into two age groups, i.e. 5–11 years and 12–17 years. The Grassy Narrows area with the English–Wabigoon river system became contaminated with Hg after 1963. Since the beginning of the 1970 s, there has been a disturbing increase in the number of suicides among the people of Asubpeeschoseewagong Anishinabek.

Trace element deficiencies that disrupt neurochemical processes may contribute to mental disorders^15,16^. Review articles have highlighted studies linking deficiencies in zinc (Zn), chromium (Cr), selenium (Se), iron (Fe), cobalt (Co), and iodine (I) to conditions such as depression, schizophrenia, dementia, cognitive impairment, autism, and ADHD^17^. Conversely, excess levels of copper (Cu), manganese (Mn), cobalt (Co), chromium (Cr), iron (Fe), and vanadium (V) may lead to depression, anxiety, psychosis, and cognitive issues. Quan et al.^18^ reviewed the association between lithium (Li), zinc (Zn), magnesium (Mg), copper (Cu), iron (Fe), and selenium (Se) and depression, recommending supplementation of Se, Zn, Mg, or Li as part of depression treatment.

Cr supplementation is a promising potential therapeutic agent for the treatment of depression. In the study by Młyniec et al.^4^ in rats it was shown that chromium salts (picolinate, chloride) affect the serotonergic and glutamatergic systems. The proof of the effectiveness of this therapy is preliminary studies on the use of chromium picolinate in patients with dysthymia and mood disorders, in patients with atypical depression, and for the treatment of premenstrual dysphoric disorder^19,20^. Research conducted in Alabama showed that this element has a protective effect against mortality due to suicide^21^. There was a statistically significant inverse relationship between Cr concentration and the number of suicide deaths in white people (*p* = 0.009, *r* = − 0.32).

Another element with anti-suicidal properties is Li^22^. This was confirmed by studies on the relationship between the Li content in drinking water and the number of suicides^23,24^. High Li concentrations in potable water were associated with decreased suicide rates^25^. Over the last 5 years, several research papers on the effects of Li on suicidal behavior have been published in the Pubmed database^25^. On the contrary, in the study by Knudsen et al.^26^ conducted on the Danish population (3.7 million people) in the years 1991–2012, it was not found that exposure to Li had a protective effect on the incidence of suicide. It should be emphasized that the tested Li level in drinking water was lower than 31 µg L^−1^ in this study.

Impaired cellular Ca homeostasis, which occurs in patients suffering from Darier-White disease (DD), is also accompanied by suicidal tendencies in addition to various neuropsychiatric disorders^27^. The symptom of suicidal thoughts was observed as a result of hypersensitivity to Ni after endovascular procedures^28^.

Most studies on the relationship between homeostasis disturbances and the occurrence of suicide incidents are based on analyzing biological fluids, e.g. blood, urine, or samples from the living environment of the studied population, e.g. drinking water, occupational exposure, etc.

Exposure to metallic elements occurs through inhalation attached to air particulates (PM), consumption of water or food, and skin contact with cosmetic or medicinal preparations. The final effect depends on the kind of element, duration of the exposure, and the dose taken, therefore it is necessary to monitor the level of toxic elements, especially in drinking water and air. The examples are the commonly monitored presence of Pb in the air or Cr occurring in water at wide concentrations ranging from 2 ppb to 100 ppb according to the Environmental Protection Agency (EPA)^24–31^.

Early research into the causes of suicide focused on psychological aspects^32^. More recently, research has focused on finding biomarkers that may be useful in predicting suicide. Patients diagnosed with major depressive disorder (MDD) and suicidal tendencies have been studied for, among other things, the volume of individual brain structures^33^, abnormalities in the hypothalamic-pituitary-adrenal (HPA) axis^34^, or abnormalities in inflammatory cytokines^35^, and thyroid stimulating hormone (TSH)^36^.

A common feature of suicide victims is undoubtedly an altered response to stress. Assuming that metal dyshomeostasis is associated with brain dysfunction, this study aimed to investigate the distribution of elements in tissues collected post-mortem from different areas of the brain and the liver as the main detoxification center in the suicide group compared to the control group. To exclude temporary exposure and examine the accumulation of elements in tissues, post-mortem samples are rarely utilized. To our knowledge, this is the first study to analyze the relationship between the level of metals in brain and liver tissues and the likelihood of committing suicide.

## Methods

### Tissue samples

Human tissue samples of the brain and liver were obtained a minimum of 24 h after death at the Department of Forensic Medicine, University of Lublin. The autopsies were performed by a professional pathologist. Tissue samples were dissected using ceramic instruments, then washed with ultrapure water with a resistivity of 18.2 MΩ cm obtained from Ultrapure Millipore Direct-Q 3UV-R (Merck, Darmstadt, Germany), dried using sterile blotting paper, placed into glass containers that had previously been cleaned with 2 M HCl, and stored at − 80 °C. Tissue samples were collected from typical anatomical locations intended for histopathological examination^37^. The tissue samples were taken from the following parts of the brain: frontal pole, precentral gyrus, postcentral gyrus, cingulate gyrus, hippocampus, head of caudate nucleus, superior longitudinal fasciculus (SLF), inferior longitudinal fasciculus (ILF), dorsal thalamus, nucleus accumbens (NAc), insula. Additionally, liver samples were collected by dissecting the tissue from the 6 th segment. All subjects were residents of south-eastern Poland. The tissues were obtained from individuals (*n* = 40) whose ages ranged from 17 to 83 years, averaging 51.5 years. Fifteen of these died from suicides(hanging, arson, fall from a height), remaining were included as the control group. People who suffered from depressive disorders were excluded from the control group. Female (*n* = 9) constituted 22.5% and male (*n* = 31) 77.5% of the study population. The age range of females was 17–83 years with an average of 49.22 ± 23.13. The age range of males was 18–83 years with an average of 52.32 ± 18.10. Body mass index (BMI) for women and men was 25.02 ± 9.91 and 25.75 ± 6.24 respectively. Comparisons of the individuals included in this study is presented in Table 1. Comparisons were performed using the Mann-Whitney U test. Fisher’s exact test was used only when comparing sex ratios across study groups. The groups did not differ significantly in terms of sex, age, body mass, and BMI as the p value > 0.05.

**Table 1:** Comparison of the analyzed groups by age, weight, sex and BMI.

Parameter	Control Group (n = 25)	Suicide Group (n = 15)	*p*
Age (mean ± SD)	52.2 ± 18.7	50.3 ± 20.4	0.76
Sex (n, %)	women: 6 (24%)	women: 3 (20%)	1.00
	men: 19 (76%)	men: 12 (80%)	
Weight (mean ± SD)	76.9 ± 23.8	73.8 ± 16.3	0.68
BMI (kg/m^2^, mean ± SD)	25.6 ± 7.2	24.9 ± 6.2	0.47

### Mineral analysis by ICP-MS

The sample preparation procedure and ICP-MS analysis conditions were described in a previous work^16^. In brief, the tissues were mineralized to remove the organic matrix using nitric acid (69% suprapur HNO_3_, Baker, Radnor, PA, USA) in the microwave mineralization system Multiwave 5000 (Anton Paar, Graz, Austria). After the mineralization step, HCl (Merck, Darmstadt, Germany) was added and diluted by ultrapure water. The elemental analysis (^107^Ag, ^137^Ba, ^44^Ca, ^140^Ce, ^52^Cr, ^133^Cs, ^63^Cu, ^56^Fe, ^71^Ga, ^157^Gd, ^39^K, ^24^Mg, ^55^Mn, ^146^Nd, ^60^Ni, ^31^P, ^141^Pr, ^195^Pt, ^85^Rb, ^169^Tm, ^66^Zn, ^75^As, ^75→91^As, ^9^Be, ^153^Eu, ^178^Hf, ^165^Ho, ^121^Sb, ^232^Th, ^238^U, ^205^Tl, ^27^Al, ^209^Bi, ^111^Cd, ^59^Co, ^163^Dy, ^166^Er, ^201^Hg, ^202^Hg, ^139^La, ^95^Mo, ^23^Na, ^208^Pb, ^105^Pd, ^78^Se, ^78→94^Se, ^147^Sm, ^118^Sn, ^88^Sr, ^159^Tb, ^47^Ti, ^51^V, ^172^Yb, ^90^Zr) was performed using the inductively coupled plasma mass spectrometer Agilent 8900 ICP-MS Triple Quad (Agilent, Santa Clara, CA, USA). The internal standard ISTD (Sc, Y, Lu) with a concentration of 0.5 ppm was added automatically. ICP commercial analytical standards were purchased from Agilent Technologies, Santa Clara, CA, USA (Mul-ti-Element Calibration Standard 2 A-Hg, Environmental Calibration Standard, Multi-Element Calibration Standard 2 A), Merck Millipore, Darmstadt, Germany (ICP-Multi-Element Calibration Standard XVII, ICP-Multi-Element Calibration Standard VI, Phosphorus ICP standard), Honeywell Fluka™ analytical standards (Platinum Standard for ICP, Palladium Standard for ICP), and Inorganic Ventures, Christiansburg, Virginia, US (Rare Earth, Standards). The validation report with BEC-background equivalent concentration, DL-detection limits, ISTD, calibration equation, and the correlation coefficient r, calibration curves, is presented in Fig. S1.

### Statistics

The statistical methods were employed for analyzing the complex relationships between elemental concentrations in autopsy samples and their potential associations with suicide. The primary statistical technique utilized was the two-way non-parametric Spearman’s rank correlation. This method is specifically chosen due to its robustness in handling ordinal data or continuous data that do not meet the assumptions of normality required for parametric tests like Pearson’s correlation^38,39^. The Mann-Whitney U test, also known as the Wilcoxon rank-sum test applied in the article, is a non-parametric statistical method used to compare differences between two independent groups when the assumption of normal distribution is not met^40,41^. To assess the significance of differences between two correlations matrices Chi-squared test was used^42^. All statistical calculations and analyses reported in this study were conducted using the R statistical environment^43^.

### Ethics statement

This study was approved by the Local Bioethics Committee of the Medical University of Lublin (number KE-0254/152/2021, approval date 24 June 2021). The biological material used in the study was collected during forensic autopsies ordered by the prosecutor in accordance with the provisions of national criminal law, which in such situations makes the collection of material and its use independent of the “written informed consent” of the deceased or their relatives. After the material was used for judicial purposes, the archived and anonymized remnants, originally intended for disposal, were used in this study with the prosecutor’s consent.

### Human experiment statement

The experiments were carried out following The Code of Ethics of the World Medical Association (Declaration of Helsinki) for experiments involving human subjects.

## Results

**Fig. 1:**
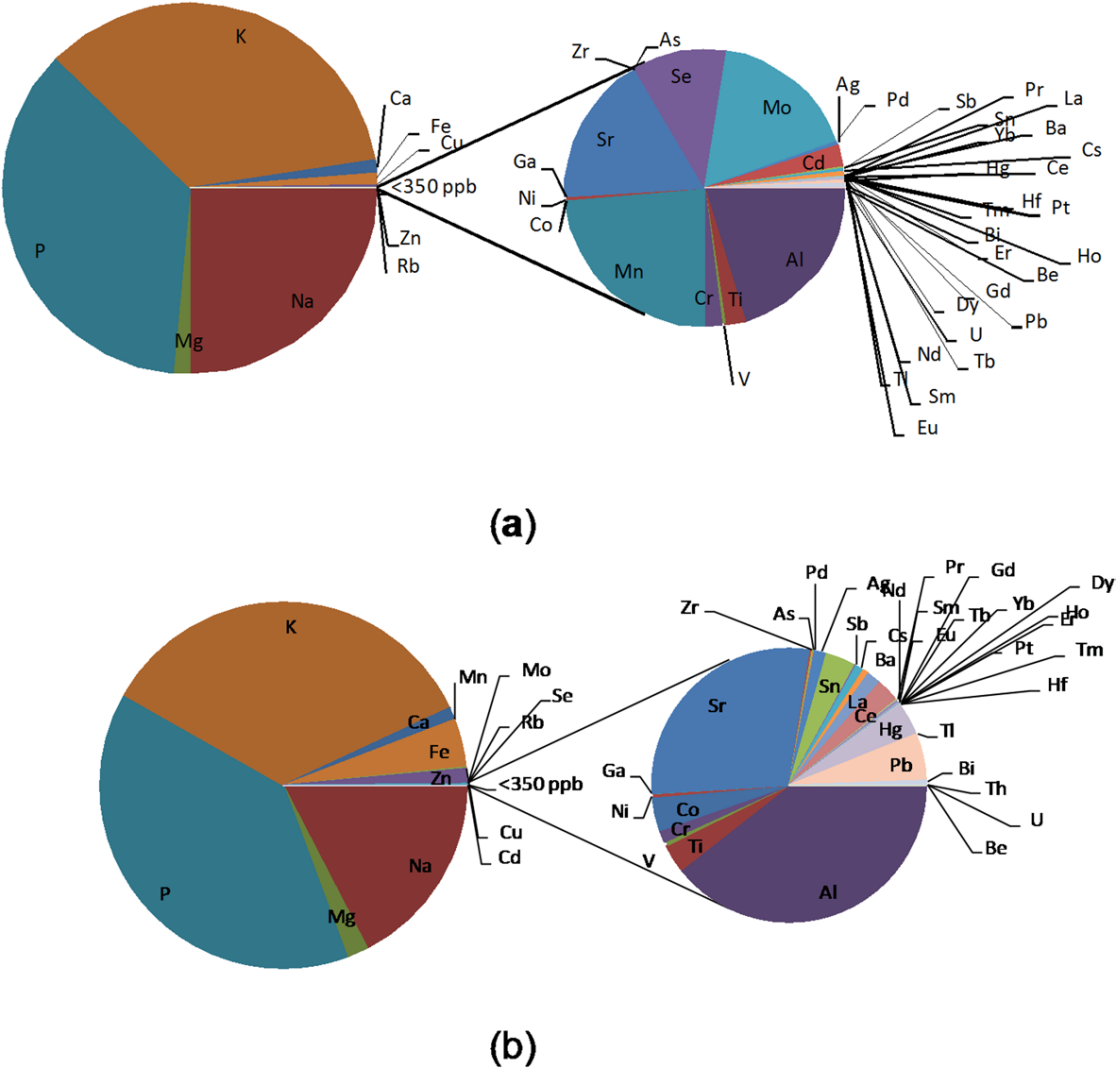
Pie of pie chart showing the mean concentration of elements in the brain **(a)** (mean of the concentration measured in the samples taken from 12 different brain areas), and the liver **(b)**. A value of 350 ppb splits the series. The symbol As is for ^75-> 91^ As [O_2_], the symbol Se is for ^78-> 94^ Se [O_2_], and the symbol Hg is for ^201^Hg [He].

The liver and brain are rich in metals, which coexist in different locations. The pie charts in Fig. 1 visualize the average element content in liver and brain samples (average of 12 areas) for the entire study population (*n* = 40).

Elements such as Ca, K, Na, P, Mg, Zn, and Fe are in the highest concentrations. In addition to essential elements, toxic elements are present in significant amounts in both organs, mainly Cd, Al, Pb, Hg, and Tl.

The contents of elements in tissue samples are characterized by quite high variability visible in the relative standard deviation (RSD%) values exceeding 25%. However, the average levels of most of these elements in brain tissue were less than 0.03 ppb (Pd and Pt), less than 0.02 ppb (Er, Ga, Yb, Tm, Dy, Tb, Eu, Sm), or even less than 0.001 ppb (U). The elements showed particular preferences for certain areas of the brain. Most metals (Bi, Mo, Zn, Ni, Mn, Cr) tended to accumulate in the nucleus accumbens, of which the highest average concentration was found for Mn (274 ppb). The content of elements in liver samples is characterized by even greater variability compared to the results obtained for the brain. The RSD% value exceeded 50% for the entire group of elements.

To handle the high dimensionality of the data set, which includes multiple measurements from different brain regions and liver tissues, Principal Component Analysis (PCA) was used^44^. The results of the PCA for all brain regions combined show that a large number of components would be needed to explain an adequate amount of variance. Even with 20 principal components, the total explained variance is 79%. The first two principal components explained only about 22% of the original variance. In the case of the liver, the 10 principal components explain about 78% of the original variance. The first two principal components explain about 33% of the variance.

Descriptive statistics were calculated for all tissues examined, separately for the control and suicide groups (Table S1). All elements were characterized by right-sided asymmetry in terms of concentration, so the T-test cannot be used to test the significance of differences between groups, as it requires a normal probability distribution in both groups, especially when groups are relatively small. Therefore, the non-parametric Mann-Whitney test was used for statistical analysis to verify mutual differences. ‘Comparisons of correlation matrices between the two study groups (suicidal vs. control) for both liver and whole brain showed significant differences. In order to compare the interrelationships between element concentrations across brain and liver, we constructed correlation matrices separately for the suicide group (R1) and the control group (R2). The analysis revealed a statistically significant difference between the two matrices (χ² = 1490.19, df = 1378, p < 0.018, z = 126.71), indicating a dissimilar pattern of correlations in the suicide group compared to the control group. This suggests that the relationships between elemental concentrations are markedly altered in the liver of individuals who died by suicide. The statistical test comparing the correlation matrices for the brain samples also showed a significant difference between the two datasets (χ² = 2998.7, df = 1378, p < 7e-122, z = 3.72), indicating a substantial dissimilarity in the correlation patterns.

This significant difference in the correlation matrices for both brain and liver samples suggests that the underlying relationships between variables are not identical in the two groups studied. Further investigation is warranted to understand the nature of these differences and their implications for the phenomenon under investigation. To identify the elements responsible for these differences between the groups, as well as the preferred sites of element accumulation, different brain areas were analysed separately.

### Comparison of elemental composition of different brain regions between examined groups

**Table 2:** Comparison of the element level (the median values) in different brain areas and liver between studied groups.

Region	Suicide > Control	Suicide = Control	Suicide < Control	(*p* < 0.05)
Frontal pole	^107^Ag, ^137^Ba, ^44^Ca, ^140^Ce, ^52^Cr, ^133^Cs, ^63^Cu, ^56^Fe, ^71^Ga, ^157^Gd, ^39^K, ^24^Mg, ^55^Mn, ^146^Nd, ^60^Ni, ^31^P, ^141^Pr, ^195^Pt, ^85^Rb, ^169^Tm, ^66^Zn	^75^As, ^75→91^As, ^9^Be, ^153^Eu, ^178^Hf, ^165^Ho, ^121^Sb, ^232^Th, ^238^U, ^205^Tl	^27^Al, ^209^Bi, ^111^Cd, ^59^Co, ^163^Dy, ^166^Er, ^201^Hg, ^202^Hg, ^139^La, ^95^Mo, ^23^Na, ^208^Pb, ^105^Pd, ^78^Se, ^78→94^Se, ^147^Sm, ^118^Sn, ^88^Sr, ^159^Tb, ^47^Ti, ^51^V, ^172^Yb, ^90^Zr	-
Precentral gyrus	^107^Ag, ^44^Ca, ^52^Cr, ^133^Cs, ^63^Cu, ^153^Eu, ^56^Fe, ^157^Gd, ^201^Hg, ^202^Hg, ^39^K, ^139^La, ^24^Mg, ^23^Na, ^146^Nd, ^60^Ni, ^31^P, ^195^Pt, ^85^Rb, ^78^Se, ^78→94^Se, ^147^Sm, ^169^Tm, ^51^V, ^172^Yb, ^66^Zn, ^90^Zr	^75^As, ^75→91^As, ^9^Be, ^140^Ce, ^178^Hf, ^165^Ho, ^121^Sb, ^232^Th, ^205^Tl	^27^Al, ^137^Ba, ^209^Bi, ^111^Cd, ^59^Co, ^163^Dy, ^166^Er, ^71^Ga, ^55^Mn, ^95^Mo, ^208^Pb, ^105^Pd, ^141^Pr, ^118^Sn, ^88^Sr, ^159^Tb, ^47^Ti, ^238^U	-
Postcentral gyrus	^107^Ag, ^27^Al, ^140^Ce, ^163^Dy, ^166^Er, ^56^Fe, ^71^Ga, ^24^Mg, ^23^Na, ^31^P, ^78^Se, ^78→94^Se, ^118^Sn, ^159^Tb, ^47^Ti, ^172^Yb, ^66^Zn	^75^As, ^75→91^As, Be, ^178^Hf, ^165^Ho, ^121^Sb, ^147^Sm, ^232^Th, ^205^Tl	^137^Ba, ^209^Bi, ^44^Ca, ^111^Cd, ^59^Co, ^52^Cr, ^133^Cs, ^63^Cu, ^153^Eu, ^157^Gd, ^201^Hg, ^202^Hg, ^39^K, ^139^La, ^55^Mn, ^95^Mo, ^146^Nd, ^60^Ni, ^208^Pb, ^105^Pd, ^141^Pr, ^195^Pt, ^85^Rb, ^88^Sr, ^169^Tm, ^238^U, ^51^V, ^90^Zr	La(0.047), Pb(0.019), Pd(0.035), Pr(0.023) Pt(0.033), U(0.048)
Cingulate gyrus	^107^Ag, ^63^Cu, ^153^Eu, ^56^Fe, ^201^Hg, ^202^Hg, ^39^K, ^139^La, ^55^Mn, ^23^Na, ^31^P, ^78^Se, ^78→94^Se, ^147^Sm, ^47^Ti, ^169^Tm, ^172^Yb, ^66^Zn	^75^As, ^75→91^As, ^9^Be, ^178^Hf, ^165^Ho, ^121^Sb, ^232^Th, ^205^Tl	^27^Al, ^137^Ba, ^209^Bi, ^44^Ca, ^111^Cd, ^140^Ce, ^59^Co, ^52^Cr, ^133^Cs, ^163^Dy, ^90^Zr, ^166^Er, ^71^Ga, ^157^Gd, ^24^Mg, ^95^Mo, ^146^Nd, ^60^Ni, ^51^V, ^208^Pb, ^105^Pd, ^141^Pr, ^195^Pt, ^238^U, ^85^Rb, ^118^Sn, ^88^Sr, ^159^Tb	U(0.042)
Hippocampus	^107^Ag, ^133^Cs, ^63^Cu, ^166^Er, ^71^Ga, ^201^Hg, ^202^Hg, ^39^K, ^95^Mo, ^31^P, ^105^Pd, ^141^Pr, ^85^Rb, ^78^Se, ^78→94^Se, ^169^Tm, ^90^Zr	^75^As, ^75→91^As, ^9^Be, ^157^Gd, ^178^Hf, ^165^Ho, ^121^Sb, ^232^Th, ^205^Tl, ^238^U	^27^Al, ^137^Ba, ^209^Bi, ^44^Ca, ^111^Cd, ^140^Ce, ^59^Co, ^52^Cr, ^163^Dy, ^153^Eu, ^56^Fe, ^139^La, ^24^Mg, ^55^Mn, ^23^Na, ^146^Nd, ^60^Ni, ^208^Pb, ^195^Pt, ^147^Sm, ^118^Sn, ^88^Sr, ^159^Tb, ^47^Ti, ^51^V, ^172^Yb, ^66^Zn	-
Head of caudate nucleus	^107^Ag, ^137^Ba, ^209^Bi, ^111^Cd, ^140^Ce, ^52^Cr, ^63^Cu, ^163^Dy, ^153^Eu, ^56^Fe, ^157^Gd, ^201^Hg, ^202^Hg, ^39^K, ^139^La, ^95^Mo, ^146^Nd, ^60^Ni, ^31^P, ^105^Pd, ^141^Pr, ^78^Se, ^78→94^Se, ^118^Sn, ^47^Ti, ^169^Tm, ^66^Zn, ^90^Zr	^75^As, ^75→91^As, ^9^Be, ^178^Hf, ^165^Ho, ^121^Sb, ^159^Tb, ^232^Th, ^205^Tl, ^238^U	^27^Al, ^44^Ca, ^59^Co, ^133^Cs, ^166^Er, ^71^Ga, ^24^Mg, ^55^Mn, ^23^Na, ^208^Pb, ^195^Pt, ^85^Rb, ^147^Sm, ^88^Sr, ^51^V, ^172^Yb	Fe(0.031), Ca(0.041)
SLF	^107^Ag, ^209^Bi, ^59^Co, Cr, ^63^Cu, ^153^Eu, ^71^Ga, ^139^La, ^31^P, ^195^Pt, ^78^Se, ^78→94^Se, ^118^Sn	^75^As, ^75→91^As, ^9^Be, ^178^Hf, ^165^Ho, ^121^Sb, ^159^Tb, ^232^Th, ^205^Tl, ^238^U	^27^Al, ^137^Ba, ^44^Ca, ^111^Cd, ^140^Ce, ^133^Cs, ^163^Dy, ^166^Er, ^56^Fe, ^157^Gd, ^201^Hg, ^202^Hg, ^39^K, ^24^Mg, ^55^Mn, ^95^Mo, ^23^Na, ^146^Nd, ^60^Ni, ^208^Pb, ^105^Pd, ^141^Pr, ^85^Rb, ^147^Sm, ^88^Sr, ^47^Ti, ^169^Tm, ^51^V, ^172^Yb, ^66^Zn, ^90^Zr	^75^As(0.018), ^60^Ni(0.031), ^147^Sm(0.039), ^51^V(0.046)
ILF	^107^Ag, ^137^Ba, ^140^Ce, ^59^Co, ^63^Cu, ^157^Gd, ^139^La, ^95^Mo, ^23^Na, ^31^P, ^105^Pd, ^141^Pr, ^195^Pt, ^78^Se, ^78→94^Se, ^66^Zn	^75^As, ^75→91^As, ^9^Be, ^178^Hf, ^165^Ho, ^121^Sb, ^232^Th, ^205^Tl, ^238^U	^27^Al, ^209^Bi, ^44^Ca, ^111^Cd, ^52^Cr, ^133^Cs, ^163^Dy, ^166^Er, ^153^Eu, ^56^Fe, ^71^Ga, ^201^Hg, ^202^Hg, ^172^Yb, ^39^K, ^24^Mg, ^90^Zr, ^55^Mn, ^146^Nd, ^60^Ni, ^208^Pb, ^85^Rb, ^147^Sm, ^118^Sn, ^88^Sr, ^159^Tb, ^47^Ti, ^169^Tm, ^51^V	-
Dorsal thalamus	^107^Ag, ^27^Al, ^140^Ce, ^52^Cr, ^133^Cs, ^63^Cu, ^153^Eu, ^56^Fe, ^71^Ga, ^201^Hg, ^202^Hg, ^39^K, ^24^Mg, ^95^Mo, ^31^P, ^78^Se, ^78→94^Se, ^47^Ti, ^169^Tm, ^172^Yb, ^66^Zn	^75^As, ^75→91^As, ^9^Be, ^178^Hf, ^165^Ho, ^121^Sb, ^232^Th, ^205^Tl	^137^Ba, ^209^Bi, ^44^Ca, ^111^Cd, ^59^Co, ^163^Dy, ^166^Er, ^157^Gd, ^139^La, ^55^Mn, ^23^Na, ^146^Nd, ^60^Ni, ^208^Pb, ^105^Pd, ^141^Pr, ^195^Pt, ^85^Rb, ^147^Sm, ^118^Sn, ^88^Sr, ^159^Tb, ^238^U, ^51^V, ^90^Zr	^44^Ca(0.007), ^166^Er (0.035), ^31^P(0.006)
NAc	^107^Ag, ^209^Bi, ^52^Cr, ^133^Cs, ^63^Cu, ^56^Fe, ^71^Ga, ^201^Hg, ^202^Hg, ^39^K, ^24^Mg, ^55^Mn, ^95^Mo, ^31^P, ^85^Rb, ^78^Se, ^78→94^Se, ^118^Sn, ^88^Sr, ^47^Ti, ^66^Zn	^75^As, ^75→91^As, ^9^Be, ^178^Hf, ^121^Sb, Ho, ^232^Th, ^205^Tl, ^238^U	^27^Al, ^137^Ba, ^44^Ca, ^111^Cd, ^140^Ce, ^59^Co, ^163^Dy, ^166^Er, ^153^Eu, ^157^Gd, ^139^La, ^23^Na, ^146^Nd, ^60^Ni, ^208^Pb, ^105^Pd, ^141^Pr, ^195^Pt, ^147^Sm, ^159^Tb, ^169^Tm, ^51^V, ^172^Yb, ^90^Zr	^78^Se(0.046), ^27^Al(0.009), ^44^Ca(0.023), ^147^Sm(0.035), ^159^Tb(0.014), ^90^Zr(0.029)
Insula	^107^Ag, ^137^Ba, ^63^Cu, ^163^Dy, ^166^Er, ^153^Eu, ^157^Gd, ^39^K, ^24^Mg, ^23^Na, ^146^Nd, ^31^P, ^195^Pt, ^47^Ti, ^169^Tm, ^51^V, ^172^Yb	^75^As, ^75→91^As, ^9^Be, ^178^Hf, ^165^Ho, ^141^Pr, ^121^Sb, ^147^Sm, ^232^Th, ^205^Tl, ^238^U	^27^Al, ^209^Bi, ^44^Ca, ^111^Cd, ^140^Ce, ^59^Co, ^52^Cr, ^133^Cs, ^56^Fe, ^71^Ga, ^201^Hg, ^202^Hg, ^139^La, ^55^Mn, ^95^Mo, ^60^Ni, ^208^Pb, ^105^Pd, ^85^Rb, ^78^Se, ^78→94^Se, ^118^Sn, ^88^Sr, ^159^Tb, ^66^Zn, ^90^Zr	^163^Dy(0.012), ^195^Pt(0.045), ^44^Ca(0.037)
Liver	^107^Ag, ^27^Al, ^44^Ca, ^111^Cd, ^140^Ce, ^52^Cr, ^133^Cs, ^63^Cu, ^163^Dy, ^166^Er, ^153^Eu, ^56^Fe, ^71^Ga, ^157^Gd, ^201^Hg, ^202^Hg, ^165^Ho, ^39^K, ^139^La, ^24^Mg, ^55^Mn, ^95^Mo, ^23^Na, ^146^Nd, ^60^Ni, ^31^P, ^141^Pr, ^195^Pt, ^85^Rb, ^78^Se, ^78→94^Se, ^147^Sm, ^118^Sn, ^159^Tb, ^47^Ti, ^205^Tl, ^51^V, ^66^Zn	^137^Ba, ^9^Be, ^178^Hf, ^232^Th	^75^As, ^75→91^As, ^209^Bi, ^59^Co, ^208^Pb, ^105^Pd, ^121^Sb, ^88^Sr, ^169^Tm, ^238^U, ^172^Yb, ^90^Zr	^140^Ce(0.037), ^153^Eu(0.009), ^157^Gd(0.029), ^139^La(0.034), ^24^Mg(0.044), ^146^Nd(0.029), ^141^Pr(0.026), ^159^Tb(0.022)

**Fig. 2:**
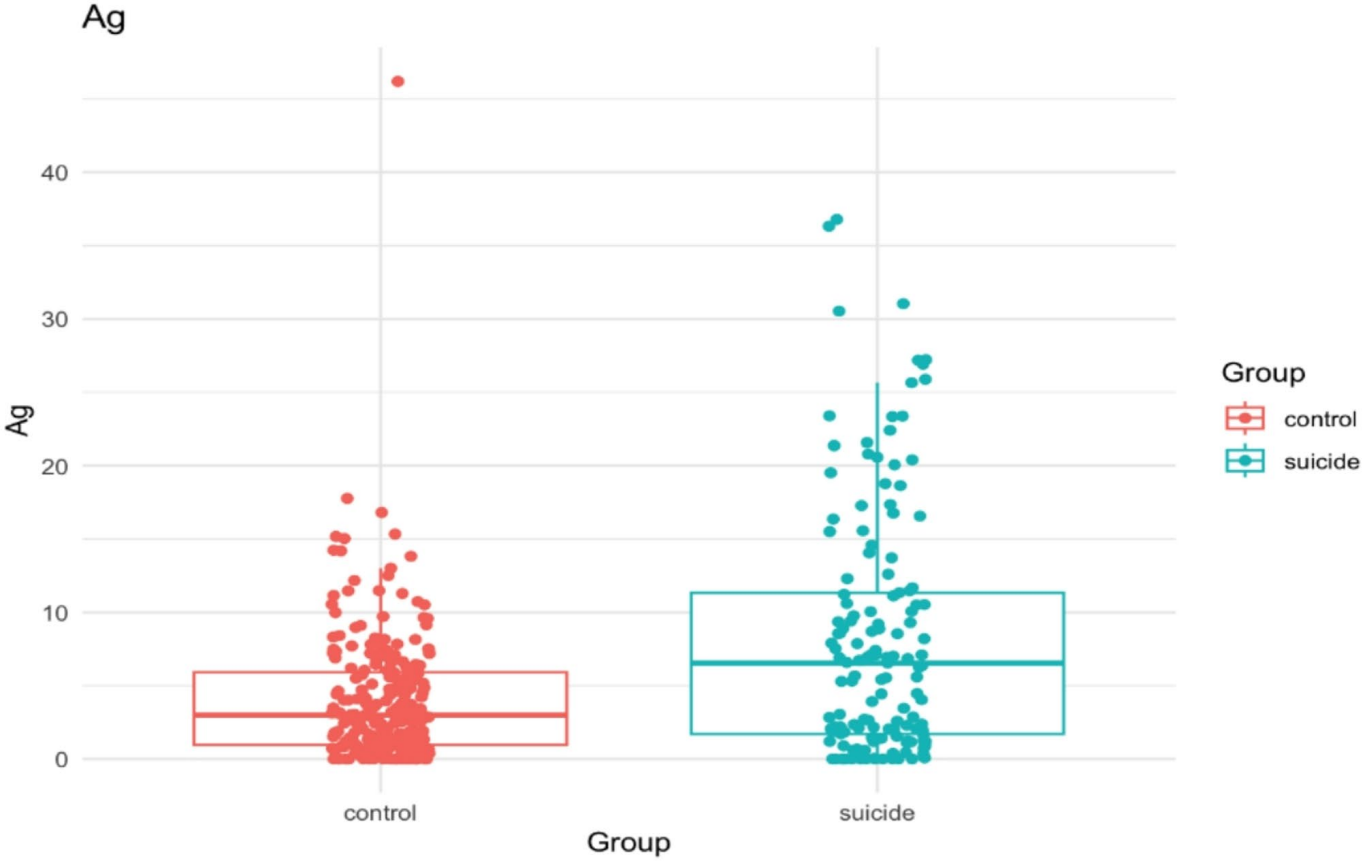
Difference in medians of Ag content in the brains between groups of suicide victims and the control group.

The data summarized in Table 2 reveal that in the suicide group, samples from specific regions of the brain contain several times more Ag compared to the control group. Although this difference in Ag content between groups did not reach statistical significance at selected brain regions, a comparison of medians for the whole brain area showed a significant difference between groups (*p* = 0.000) (Fig. 2). A comparison between groups was performed using the Mann–Whitney U test.

The examined tissues can be ranked depending on how many times more Ag is present in the group of suicides compared to controls in the following series:

frontal pole (3.08) > hippocampus (2.89) > liver (2.87) > precentral gyrus (2.83) > head of caudate nucleus (2.44) > postcentral gyrus (2.24) > dorsal thalamus (2.17) > SLF(2.09) > NAc(1.94) > cingulate gyrus (1.35) > ILF (1.26).

**Fig. 3:**
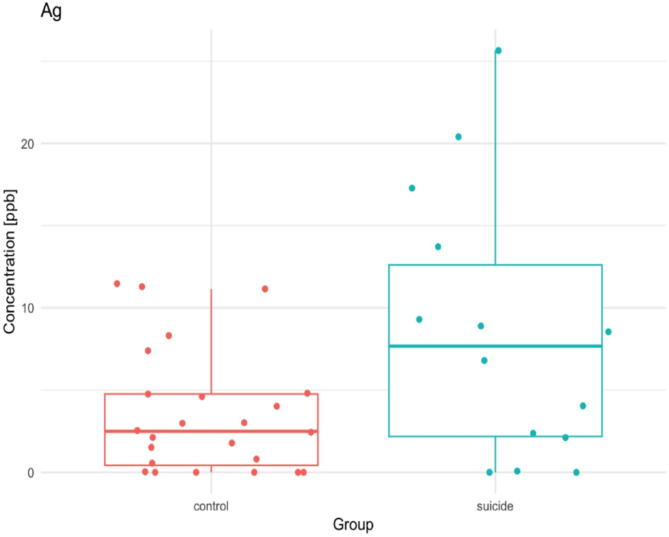
Differences in the ^107^Ag content (median, ppb) in the frontal pole between the studied groups.

The biggest difference in Ag content occurs in the frontal pole, where the suicide group has approximately three times higher Ag content than controls (Fig. 3).

The summary in Table S1 also indicates a higher Cu content in almost all tested samples, except for the postcentral gyrus, in the suicide group compared to the control. However, changes in Cu content do not exceed 1.3 times, but they occur systematically.

**Fig. 4:**
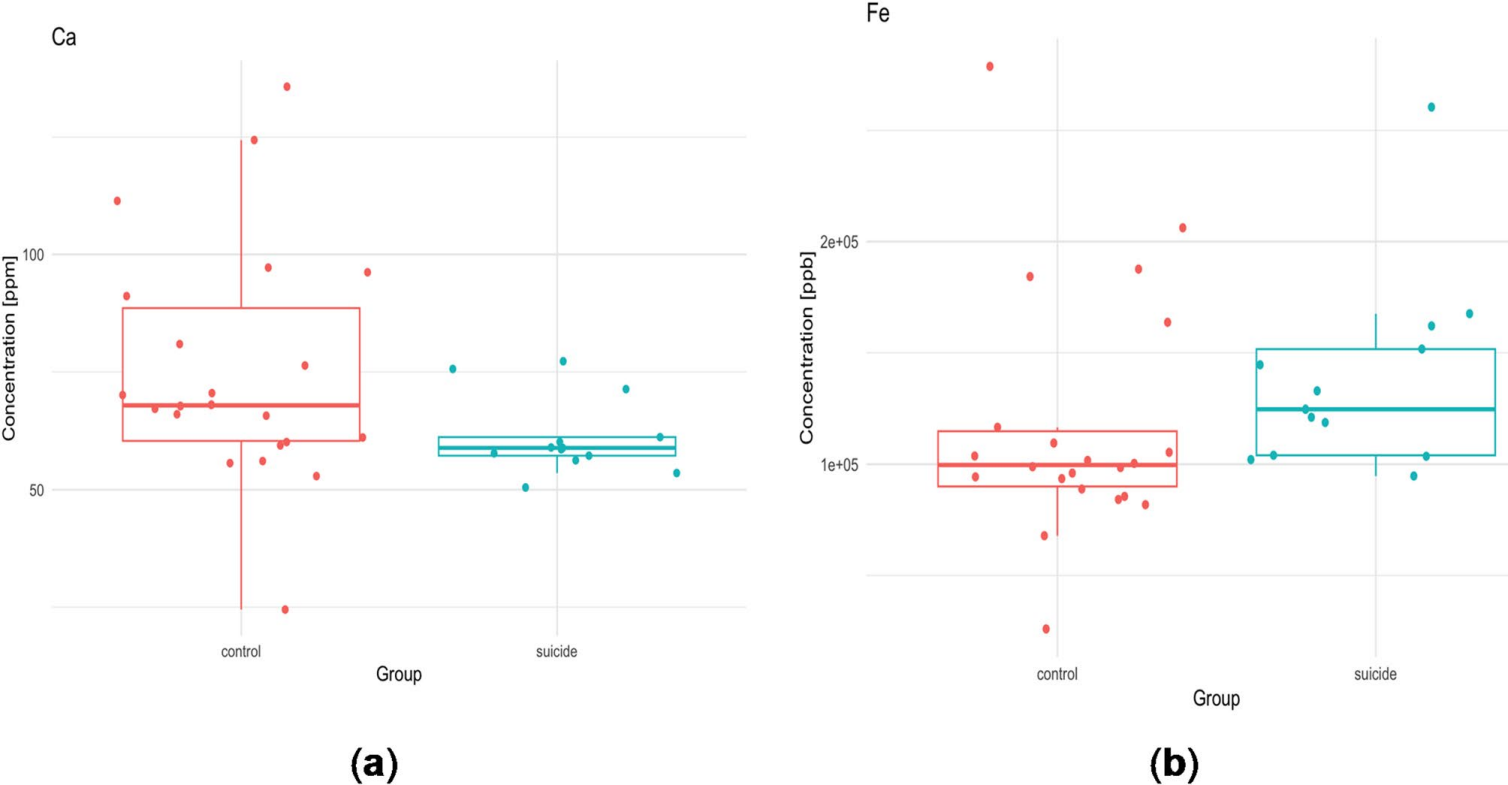
Statistically significant differences in the ^44^Ca (median, ppm) (*p* = 0.041), and ^56^Fe (median, ppb) (*p* = 0.031) contents in the head of caudate nucleus between studied groups.

In most areas, the median Ca values ​​in the suicide group are lower compared to the control group. Only in the frontal pole (control group: 105.36, suicide group: 109.15 ppm) and in the precentral gyrus the situation is reversed (control group: 76.952, suicide group: 88.588 ppm). Typically, the reduction in Ca levels compared to the control group ranges from 10 to 20% and is the smallest for the inferior longitudinal fasciculus of the brain (1.7%) and the largest for the hippocampus (21.6%). The significant difference in Ca concentration was observed in the head of caudate nucleus. In the suicide population there was significantly less of this element (mean value 58.894 ppm) than in the controls (67.912 ppm), whereas for Fe we can observe an inverse relationship. The suicide group had a significantly (*p* = 0.031) higher mean value (124728.428 ppb) compared to the control group (99679.482 ppb) (Fig. 4).

**Fig. 5:**
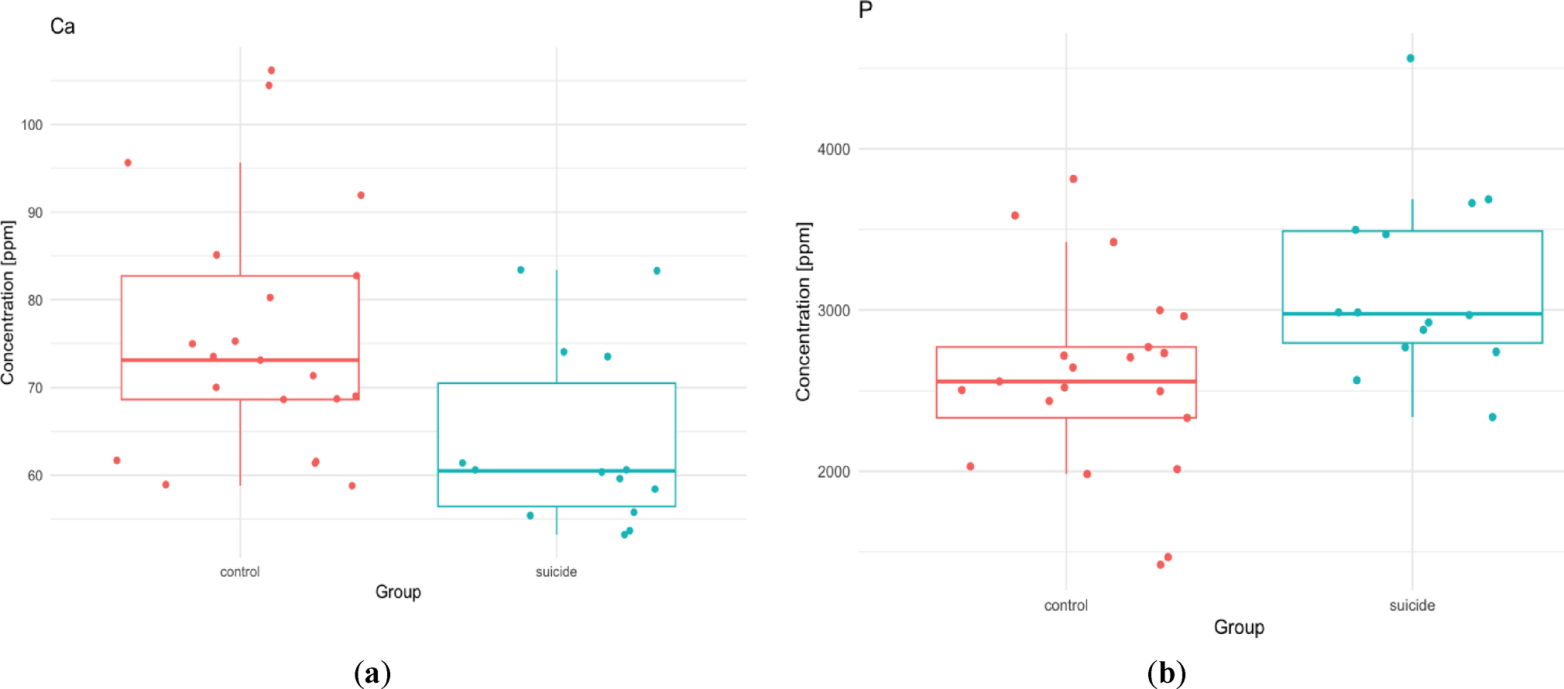
Statistically significant differences in the ^40^Ca content (median, ppm), and ^31^P in the dorsal thalamus.

In the dorsal thalamus, there were also a statistically significant differences for Ca (*p* = 0.007), and P (*p* = 0.006). The suicide group had a higher average P content (control 2577.168 ppm; suicide group 3144.562 ppm), but a lower Ca content (control 75.874 ppm, suicide group 63.813 ppm) compared to the control (Fig. 5).

In the suicide group, the P content is higher compared to the control for all tissues tested. These are not changes that reached statistical significance except for the dorsal thalamus area. They usually lie in the range of 4.5–7.5%. The least significant differences in median values occur for the SLF (1.9%) and ILF (1.2%) areas, and the largest for the hippocampus (15%), head of caudate nucleus (11.5%), and dorsal thalamus (16%).

Analysis of the correlation matrices prepared for each brain area shows that the number of negative correlations (blue) and the positive correlations (red) changes in the suicide group compared to the controls.

**Fig. 6:**
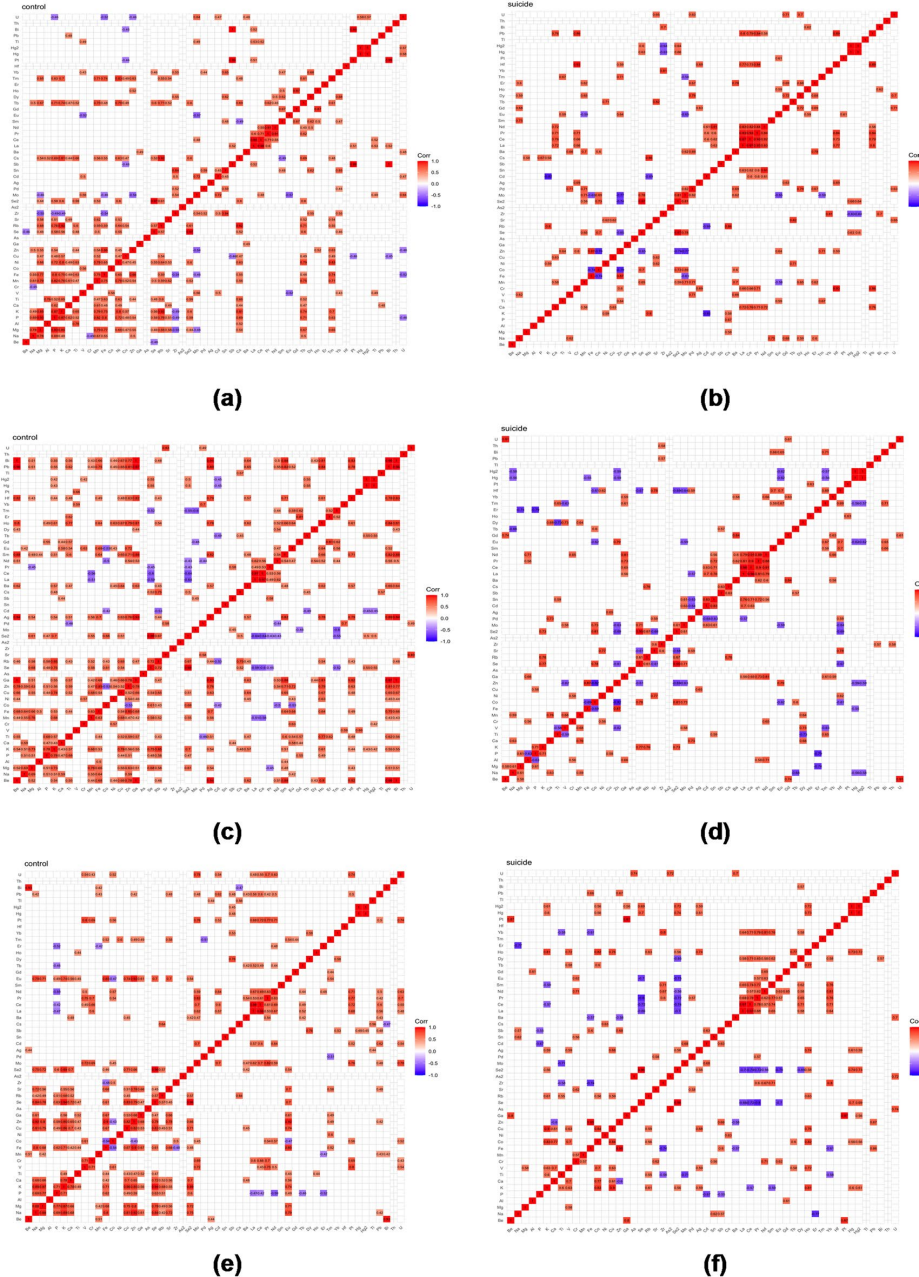
Spearman rank-order correlation matrix for the **(a)** control **(b)** suicide group in the hippocampus; the **(c)** control **(d)** suicide group in the head of caudate nucleus; the **(e)** control **(f)** suicide group in SLF area.

The number of negative correlations decreased in the cingulate gyrus, hippocampus, head of the caudate nucleus, and dorsal thalamus. The most significant decrease was in the frontal pole, where negative correlations dropped by nearly 90% in the suicide group compared to the control group. Meanwhile, the number and strength of negative correlations increased in the nucleus accumbens, ISF, and SLF regions in the suicide group. The weakening of the negative and positive correlation strength is visible in Spearman rank-order correlation matrices for the hippocampus, the head of caudate nucleus, and the SLF regions (Fig. 6).

### Mineral status of the liver

**Fig. 7:**
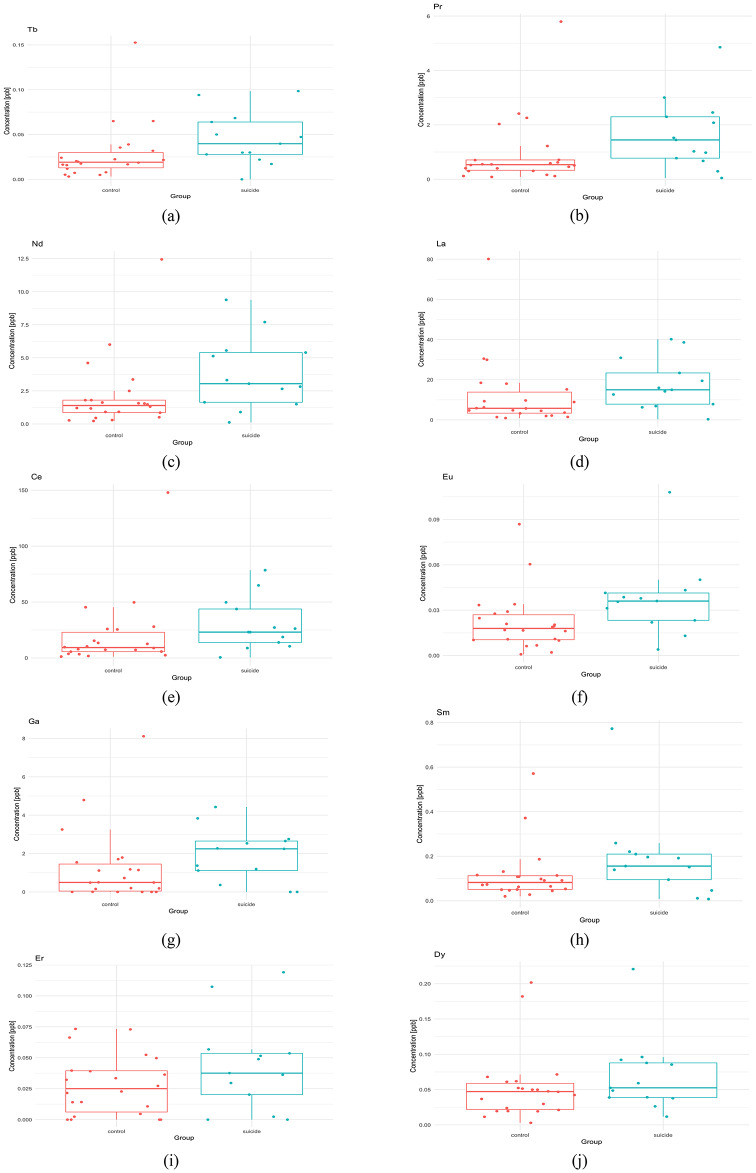
The differences in the element content in the liver between suicide and control groups for elements achieving statistical significance: **(a)** Tb (*p* = 0.022); **(b)** Pr (*p* = 0.026); **(c)** Nd (*p* = 0.029); **(d)** La (*p* = 0.034); **(e)** Ce (*p* = 0.037); **(f)** Eu (*p* = 0.009); and elements did not achieve statistical significance(*p* > 0.05): **(g)** Ga; **(h)** Sm; **(i)** Er; **(j)** Dy.

**Fig. 8:**
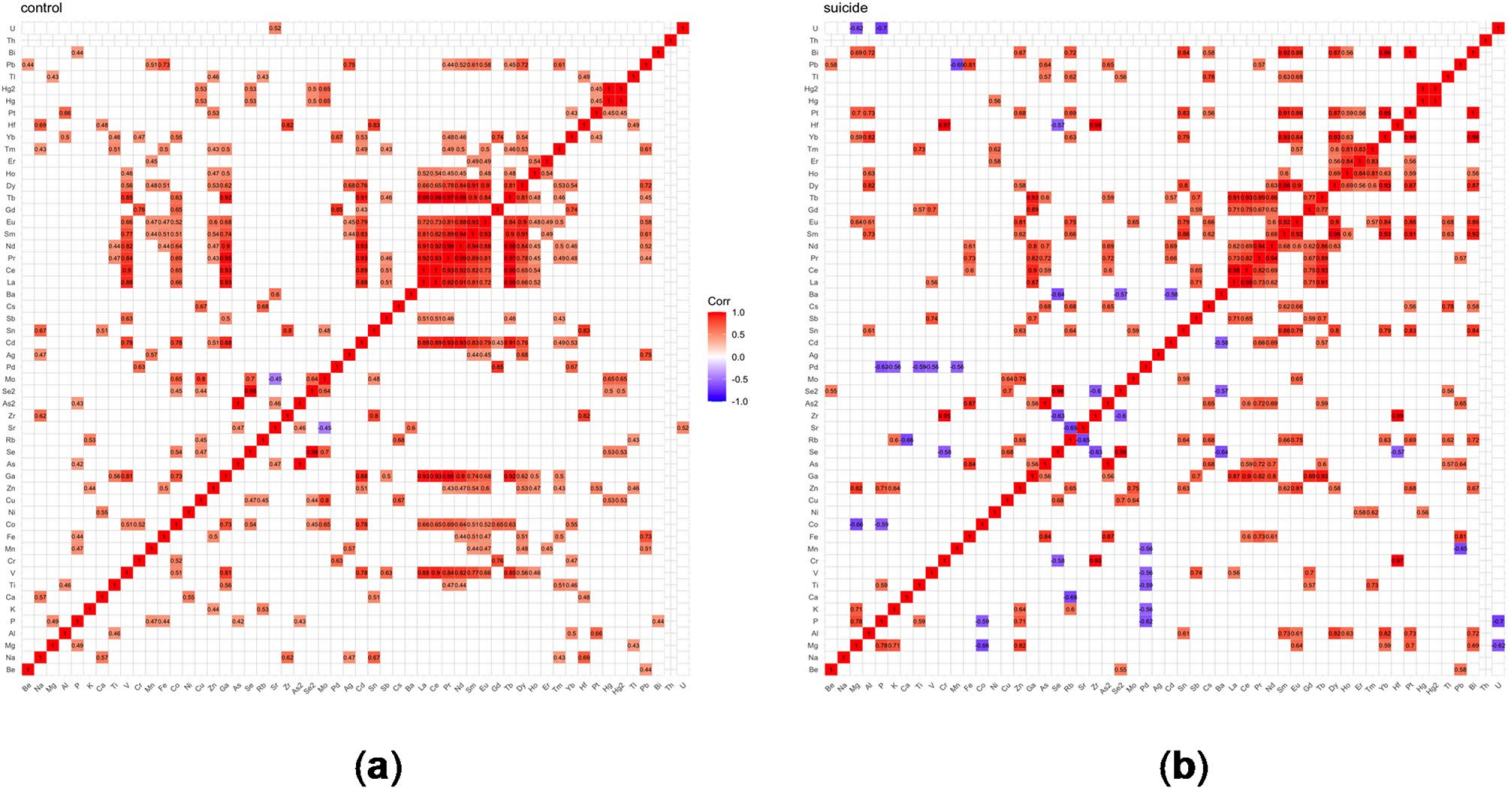
Spearman rank-order correlation matrix for the **(a)** control **(b)** suicide group in the liver.

In the suicide group, higher concentrations of toxic metals (Al, Cd, Cr, Hg, and Tl) are present, as well as several lanthanide elements that reached statistical significance (*p* < 0.05) (Table S1, XII liver). A statistically significant difference between the groups was specifically found for Nd, Ce, Eu, La, Pr, Tb, and Gd (Table 2; Fig. 7). Referring to the correlation matrices, it can be observed that the number and strength of negative correlations increase in the suicide group compared to the control group (Fig. 8).

## Discussion

Based on the performed analysis, it can be concluded that liver and brain tissues have similar elemental compositions, with the liver tending to accumulate elements such as Fe, Mo, Mn, and Cd preferentially compared to the brain. In turn, Cu, Na, P, and K are less abundant in the liver than in the brain. These differences between liver and brain tissues contribute to our understanding of organ-specific elemental accumulation and provide insights into the potential role of specific elements in the function of these organs.

The brain accumulates metals to use them for its functions^45,46^. Fe, Cu, and Zn, mainly found as cofactors in metalloproteins, are used for myelin synthesis and neurotransmission. The total concentration of essential metals is not so important as how they are sequestered in different brain regions. Each brain region has a unique composition of metals to ensure proper functioning. It is an inappropriate sequestration that is mainly responsible for brain pathology. The presence of toxic metals, even in trace amounts, is undesirable. Unfortunately, the neurotoxic metals Hg, Pb, As, and an exceptionally high amount of Al were found in the tissues examined.

Depression is known to be associated with high rates of suicide^47^. The etiology of depression is being investigated, but there are still many uncertainties, and diagnosis is primarily based on the assessment of clinical symptoms. A study using functional magnetic resonance imaging (fMRI) to assess neural networks and brain activity has shown abnormal brain areas and disruption of functional connectivity networks in people with depression. Functional dysfunction is the result of dysfunction at the molecular level, including element accumulation or metal sequestration dysfunction. Our study showed that both the liver and brain tissue of the suicide group differed in these respects from the control group, which recruited people who had died of other causes.

A higher content of toxic elements (Al, Cd, Cr, Hg, and Tl) and elements from the lanthanide group (Nd, Ce, Eu, Gd, La, Tr, Tb) was found in the liver tissue of the suicide group. Our previous work confirmed the increased affinity of selected elements from the lanthanide family to the arachnoid mater in a group of suicide victims^48^. The concentration of lanthanides was two to three orders of magnitude higher in the meninges than, for example, in the anterior chamber of the eye^49,50^. To date, lanthanides have not been shown to play a role in physiological responses in the body. It is therefore reasonable to assume that they may be a source of toxicity. Lanthanides are widely distributed in the Earth’s crust and their use is increasing in various industries, from the petroleum industry to medical diagnostics and therapy. As a result, the amount of these elements in the environment, and therefore human exposure, is increasing. The toxicity of lanthanide compounds is variable and depends primarily on the dose and route of exposure. Toxicological studies in animal models have shown that approximately 80% of Ce, Pr, Eu, Dy, Lu, and Yb accumulate in the liver after intravenous injection^51^. Lanthanides are thought to act both as analogs and as competitors for calcium ions in biological processes^51,52^. The mechanism of the neurotoxic effect of lanthanides is probably related to the influence of ions of these elements on the flow of neuronal ions, e.g. through calcium channels, and the efficacy is inversely proportional to the radius of the hydrated ion according to the following order: Lu > Er > Tb > Eu > Nd > Ce > La. To our knowledge, there are no studies on the negative effects of lanthanide on mental health, suicidal ideation, and suicide rates. In 2020, an animal model study showed the advantageous influence of CeO_2_ nanoparticles to increase neuronal plasticity and alleviate neurodegeneration by inhibiting inflammatory and oxidative markers in depression^53^. Further work is therefore needed to clarify these inconsistencies and to compare study conditions.

Our study found that Ag levels in the suicide group were several times higher than in the control group, both in different areas of the brain and in the liver. The most common form of Ag exposure is nanoparticles, which are widely used in cosmetics and medicine because of their strong antibacterial properties. Because silver nanoparticles cross the blood-brain barrier, they pose a threat to brain function^54^. The work of Dziendzikowska showed that the ratio of serotonin to dopamine neurotransmitters was disturbed in rats treated with AgNPs or Ag ions. In addition, an induction of peripheral inflammation and an increase in serum levels of inflammatory mediators were observed in rats exposed to AgNPs. Exposure to AgNPs during pregnancy was also found to cause depressive-like behaviour in rat offspring^55^. The neurotoxic effects that occur during neuronal development are likely to be responsible for this finding. The greatest difference in Ag levels between the groups studied occurred in the area of the frontal pole.

The frontal pole of the brain is known to be involved in higher order processes, both social, emotional and cognitive^56,57^. Research suggests that major depressive disorder is associated with structural disturbances in the form of reduced grey matter volume in the prefrontal cortex and metabolic disturbances in the serotonergic system of the frontal pole^58–61^.

The higher levels of Cu and Fe in tissue samples from suicide victims deserve attention. Both elements are essential trace elements and, together with Zn, are abundant in the brain. However, due to their redox activity, Cu and Fe in excess can have toxic effects, causing the endogenous production of reactive oxygen species (ROS) such as superoxide (O^2−−^), hydrogen peroxide (H_2_O_2_) and the most reactive hydroxyl (OH^−^), which threaten the brain by affecting mitochondrial function and causing damage to carbohydrates, lipids, proteins and DNA^62^. However, it should be emphasised that free radical production is also caused by excess redox-neutral metals, such as Zn, e.g. by activation of protein kinase C, NADPH oxidase and NO synthase^63,64^. Therefore, dysregulation of metal homeostasis, especially Fe, Cu and Zn, may contribute to brain disease and dysfunction. High levels of metals threaten brain function by causing oxidative stress and inflammatory responses, resulting in oxidative damage, protein aggregation, neurotoxicity and neurodegeneration^65^. Recent research points to pro-inflammatory states as a predictor associated with suicide risk, even independent of the presence of depressive states. In 2019, a study was conducted on the relationship between levels of inflammatory indicators (C-reactive protein (CRP), white blood cell count (WBC) and immunoglobulin E (IgE)) and the Dietary Inflammatory Index^®^ (DII) on suicidal ideation (SI) and major depressive disorder (MDD)^66^. The study identified the DII as an indicator associated with suicidal ideation in US adults with and without depression. The impact of inflammation on neuropsychiatric symptoms and suicidality is complex, and the evidence to date supporting this association has been summarised in the review article by Brunden et al.^67^.

Previous research confirms that inflammation occurs in suicidal individuals. Increased inflammation in the brains of suicide victims has been reported by Tonelli et al.^68^ who showed increased IL-4 and IL-13 mRNA transcripts in the orbitofrontal cortex, Steiner et al.^69^ who showed increased microgliosis, Lindqvist et al.^70^ showing increased levels of IL-6 in cerebrospinal fluid (CSF), Isung et al.^71^ finding lower levels of neuroprotective IL-8 in CSF, Pandey et al.^72^ finding increased mRNA and protein levels of IL-1β, IL-6 and tumour necrosis factor alpha (TNF-α) in brain tissue of suicide victims. In recent years, many studies have been published confirming the usefulness of inflammatory biomarkers in predicting suicide^73^. Undoubtedly, increased inflammation of the nervous system together with increased levels of inflammatory markers (interleukin-6 and other cytokines in plasma or cerebrospinal fluid) are factors that increase the risk of death by suicide^74^.

The relationship between Cu homeostasis and depression is still under investigation and the results are inconclusive^75^. Oxidative stress is thought to be the basic molecular mechanism of Cu-induced depression^76^, due to the redox activity of copper and its involvement in the Fenton reaction^77^. Excessive Cu intake is known to cause oxidative damage by activating antioxidant protective signals and toxicity by reducing the activity of antioxidant enzymes such as superoxide dismutase (SOD), catalase (CAT) and glutathione peroxidase (GSH-Px)^78^. However, it should be remembered that dietary Cu deficiency may also contribute to the development of depression. This has been confirmed by cross-sectional studies of more than 14,000 people, including Americans^79^ and others^80,81^. Typically, the excess Cu in the suicide group was accompanied by a higher Fe content, which is understandable since they do not compete with each other, on the contrary, as Cu intake increases, so does Fe content^82,83^.

It is also possible to biomineralise Fe and Cu in the form of nanoparticles (zero-valent), which are much more reactive^84^. The accumulation of metallic nanoparticles can cause local overproduction of ROS, leading to oxidative stress, inflammation and ultimately neuronal failure. In 1992, Kirschvink et al. detected magnetic magnetite nanocrystals in human brain tissue^85^. More recent research in 2021^86^ confirmed the possibility of bio-mineralisation of nanoscale metallic Cu and Fe in the brain. It should be emphasised that chemical speciation of human tissues is much less common due to the need to use highly specialised equipment, e.g. scanning transmission X-ray microscopy (STXM).

In our study, Ca levels were lower in the suicide group than in the controls. Ca affects many functions of nerve cells, such as excitability, nerve conduction and synaptic transmission. Lower Ca levels as a predictor of suicide have already been considered. A 1998 population-based cohort study hypothesised that calcium channel blockade increases the risk of suicide^87^. The study included about 150 municipalities in Sweden and a population of about 3.5 thousand people. The risk of suicide in users (650) compared with non-users (2780) of calcium channel blockers was statistically significant (*r* = 0.36, *p* < 0.001). The risk of suicide among calcium channel blocker users was confirmed in subsequent years^88^. Although in our study Ca levels were reduced in most suicide samples, the difference between groups in its content reached statistical significance for the head of the caudate nucleus, the dorsal thalamus, the NAc and the insula. The dopamine system in the striatum has been studied in suicidal depression since the last century^89^. Although the basal ganglia are primarily associated with motor control, their role in emotional and cognitive processes should not be forgotten. The connection between the basal ganglia and the cerebral cortex regulates motor and emotional functions. Damage to these basal nuclei leads to disturbances in cortical-subcortical connections (dorsolateral prefrontal loop, orbitofrontal loop and anterior cingulate loop), resulting in cognitive and emotional disorders such as depression, mania, anxiety and apathy^90^. Resting-state functional magnetic resonance imaging (rs-fMRI) was used to investigate functional connectivity (FC) in the striatal circuit. Measurements in groups of depressed patients even suggest that FC disturbances were observed in the striatal circuit, which may be a characteristic marker of MDD and have the potential to predict MDD risk^91–93^. Significantly lower Ca levels in the caudate nucleus may result in neurotransmitter dysfunction. It should be noted that the standard deviation of the quantitative determinations in the suicide group is much smaller (SD = 8.229) compared to the control group (SD = 25.344). Therefore, the coefficient of variation (CV) for the suicide group, which is 13.4%, is lower than for the control group (33.6%), which indicates that the distribution of this characteristic, the Ca content in the caudate nucleus, varies very little for the suicide group. In the suicide group, statistically lower Ca levels were found in the NAc compared to the control group. The NAc is also a component of the striatum that receives information from both the limbic and motor systems^94^. Many studies have shown that the NAc is associated with depression^95^. The pathogenesis of these mental disorders is supported by the fact that they can be treated with NAc-targeted therapies^96^.

An interesting study was conducted in 2016 on a large population of over 250 patients who committed or attempted suicide and a control group^97^. Differences in the volume of subcortical structures (caudate nucleus, pallidum, putamen, nucleus accumbens, hippocampus, amygdala, ventral diencephalon and thalamus) were examined using 1.5 T magnetic resonance imaging. Only negative correlations were found between the volume of the left (*r* = −0.35, *p* = 0.002) and right (*r* = −0.41, *p* < 0.0005) nucleus accumbens and the mortality of the last suicidal act. The authors of the study emphasise that the study confirms the role of the NAc in decision-making or in the processing of psychological pain.

In the insula samples, a statistically significant correlation was found for several elements, among others Ca. It should be noted that the suicide group had a lower Ca content (mean control value: 71.159 ppm; mean suicide value 61.674 ppm). Lower Ca content in the suicide group may confirm poorer functional connectivity the the insula region.

Hao’s 2023 work^98^ identified three classes of psychological pain in MDD patients. They found that people attempting suicide had the highest levels of pain avoidance. Using machine learning, pain avoidance was distinguished as a basic feature of the suicide attempt classification model. The authors of the paper based their conclusions on the basis of brain imaging and patterns of functional connectivity (FC) of the brain between the neural circuits responsible for pain processing, i.e. the left amygdala and the right insula, the right orbitofrontal cortex and the left thalamus, the left anterior cingulate cortex and the left insula, the right orbitofrontal, amygdala and gray matter volume (GMV) of the right thalamus. Other authors also confirm that hidden suicide associations are accompanied by network changes in the connectivity of the insula and amygdala identified using magnetoencephalography (MEG)^99^. Among people with a history of suicide attempts, blunted activity of the middle and posterior insula was confirmed^100^.

The insula is a multimodal region composed of three functionally distinct regions, (ventral anterior insula (vAI), dorsal anterior insula (dAI), and posterior insula (pI)) that plays an important role in the processing of emotion and cognition^101^. Previous studies have shown that the transformation of suicidal thoughts into action is accompanied by abnormal insula activity^102,103^. Fang et al.^104^ examining the neural mechanism of this process, they found two distinct neural pathways related to the insula. The study found that suicide attempters had lower resting-state functional connectivity (FC) between the ventral anterior insula (vAI) and the superior/middle frontal gyrus (vAI-SFG, vAI-MFG), as well as between the posterior insula (pI) and MFG (pI-MFG).

It is known that depressive disorders involve functional abnormalities in various brain regions^105–107^. The thalamus is considered an important causal center in depression. In the dorsal thalamus, there were a statistically significant correlation for several elements, namely for Ca, and P. It should be noted that the suicide group had a higher average P content (control 2577.168 ppm; suicide group 3144.562 ppm), but a lower Ca content (control 75.874 ppm, suicide group 63.813 ppm) compared to the control.

Granger causality (GCA) examination of brain connectivity abnormalities confirmed enhanced bottom-up connections from the thalamus to various cortical and subcortical areas responsible for excessive sensory information and reduced top-down connections from these regions to the thalamus responsible for poor downlink suppression of negative emotions^108^. In addition to functional abnormalities in the thalamus, macroscopic structural abnormalities have been detected and have been associated with the neuropathology of major depressive disorder^109^.

The brain is rich in P because of the presence of phospholipids. In the study, P levels were higher in all brain regions in the suicide group than in the control group. However, the higher P content in the suicide group was only statistically significant for the thalamus area. This may be explained by the fact that the individuals came from a region of Poland with a typical agricultural character. The source of P exposure may be organic phosphorus fertilisers, which have neurotoxic effects. Several publications have raised concerns about a link between these pesticides and depression, suicidal ideation and even the number of suicides^110–112^. Organophosphates are considered to be cholinesterase inhibitors, an enzyme in the central nervous system that controls nerve impulses at cholinergic synapses. As cholinesterase inhibitors, organophosphates are responsible for the occurrence of cholinergic syndrome, which is characterised by the accumulation of acetylcholine in neuronal connections^113^. There are studies confirming that the suicide rate among farmers is several times higher than in other professions, especially in areas where exposure to organophosphates is high^114^.

Weakening in Spearman’s rank-order correlation matrices was evident for the hippocampus, head of the caudate, and SLF areas. The first report on the relationship between MDD and hippocampal volume was published in 1996^115^. The report looked at smaller hippocampal volumes in MDD individuals compared to controls. Research since then has confirmed this property of the brain in people with MDD^116^. However, there is no clarity about the cause and effect relationship of this effect. There is no doubt that long-term depression leads to a reduction in hippocampal volume^117,118^. At the same time, there is genetic evidence that structural damage to the hippocampus is associated with the etiology of depression^119^.

An attempt to explain the relationship between the hippocampus and depression is the neurotoxicity hypothesis^120^, which assumes that the reduction in hippocampal volume is the result of damage caused by long-term stress, abnormalities in the functioning of the hypothalamic system, the pituitary-adrenal axis (HPA), and, consequently, chronic exposure. to glucocorticoids, reduced neurogenesis, as well as glial-derived neurotrophic factors such as brain-derived neurotrophic factor (BDNF)^121,122^. These mechanisms may occur simultaneously, so it is possible that they act additively or synergistically. The report by Gerritsen and colleagues^123^ on a group of 636 participants verified these doubts to a small extent. The report confirmed that more episodes of depression are associated with smaller hippocampal volume, but this is not related to HPA abnormalities. Smaller hippocampal volume in depression does not apply to late-onset depression (> 50 years) (LOD), where a smaller volume of the entorhinal cortex was observed, which is characteristic of the preclinical phase of Alzheimer’s disease^124^. This supports the hypothesis that depression is a risk factor for Alzheimer’s disease^125,126^.

Although there were no statistically significant differences in the levels of elements in the hippocampus between the groups, an overall decrease in both positive and negative correlations was observed in the suicide group. This may be related to the structural changes in this area of the brain described above. There are no studies in the literature regarding the elemental composition of the hippocampus. The SLF, in turn, is part of the system of longitudinal association fibres connecting the frontal and parietal lobes, with a clear asymmetry between the two hemispheres^127^. This bundle of fibres is involved in several functional correlates. Individuals with major depressive disorder MDD were found to have significant changes in the microintegrity of the left SLF^128^. There are many studies that support the fact that major depressive disorder (MDD) affects the anatomy and functionality of the cerebral white matter structure in the area of the SLF^129^. The research confirms that the MDD group had reduced Fractional Anisotropy (FA) values in the bilateral area of the superior longitudinal fasciculus (SLF) compared to the control group. Research by Kang et al.^130^ using 3D structural MRI scans processed by Tracts Constrained by UnderLying Anatomy suggests that reduced white matter integrity in these areas is the primary pathophysiology underlying impairments in emotion recognition and description in MDD.

In the SLF area, a statistically significant correlation occurred for several elements, namely for Sm, Ni, V and As. It should be noted that the suicide group had higher mean As content (control 0.00 ppb; suicide group 0.419 ppb), but lower Ni (control 5.269 ppb, suicide group 2.056 ppb) and V (control 2.039 ppb, suicide group 1.142 ppb) compared to the control (Table 1S, 7). Epidemiological studies confirm that exposure to As can cause multiple mental health effects^131^. Tyler et al.^132^ in their review presented a coherent synthesis of studies that focused on the neural mechanisms of dysfunction after arsenic exposure, including the increased incidence of Alzheimer’s disease. Studies in a mouse model have confirmed that subchronic exposure to As induces anxiety-like behaviors and intensifies depression-like behaviors^133^. There are many sources of exposure to As, such as drinking water, food (especially vegetables, shellfish, and seaweed), smoking, air, and cosmetics, with drinking water being the most commonly studied route. As-induced neurodegeneration is the result of various mechanisms, the dominant ones being oxidative stress, inflammation, and mitochondrial dysfunction^134^.

There is an ongoing debate about Ni in the context of its toxicity^135^. It is known to be ubiquitous in food and water. Ni is an essential trace element in several animal species and probably in humans^135,136^. Nickel exposure is considered a risk factor for brain dysfunction and behavioral and neurological symptoms in humans^137^. However, there is a lack of data on the association of Ni levels in various biological fluids with specific symptoms.

The limitation of the study is the relatively small number of suicides and the lack of information on environmental exposure to other substances. Extending the study to a larger population also from other countries could demonstrate and confirm the statistically significant links between metal accumulation and suicide risk observed in the study. Furthermore, understanding the preferential accumulation of metals in the brain may facilitate the identification of new sources of exposure.

## The strengths, limitations, and future research

The strength of the study is that it included many samples from 11 brain regions and the livers of 40 individuals Approximately 500 samples were examined and more than 50 elements were measured. The study is limited by its small population size. Given the increasing number of suicides and mental illnesses, future metallomic studies should focus on a larger patient population to assess the influence of factors such as age, sex, environment, country of origin, chronic diseases, addictions (alcohol, cigarettes, psychoactive substances) or medications taken. It should be emphasised that only post-mortem examinations allow elemental analysis in different tissues and organs. In the group of people who have attempted suicide, certain trends have been observed with regard to the accumulation of trace elements in the central nervous system and the non-homogeneous distribution of elements in different areas, but knowledge in this area is still limited. Further research should help to formulate preventive principles, as the source of exposure to toxic elements absorbed through the skin, inhalation or the digestive system is the living environment and diet.

## Conclusions

The mental health epidemic has become increasingly evident in recent years, reflected in the rise of various disorders that often lead to suicide attempts. Despite extensive research, the exact causes of mental illness remain unclear. Without a precise understanding of the causes, we have numerous treatments, most of which are symptomatic. In the current study, a striking feature of the suicidal brain was observed: a weakening of the strength of inter-item correlations in most brain regions, as well as a decrease in the number of negative correlations in the frontal pole area. Notably, there was a decrease in Ca content and an increase in Ag, P, Fe, and Cu. Higher levels of highly toxic elements such as Hg, Cd, Pb, As, Al, and lanthanides were found in liver tissue compared to the control group. Research on metal metabolism in the brains of suicide victims should continue on a larger scale. The aim of future research should be to identify at-risk groups and explore potential prevention strategies, as the primary sources of macro- and microelements, as well as toxic elements, are both the living environment and diet.

## Electronic supplementary material

Below is the link to the electronic supplementary material.

Supplementary Material 1

Supplementary Material 2

## Data Availability

Jolanta Flieger should be contacted in case of any query or any data for the study.The datasets analysed during the current study are available in the Zenodo repository, doi: https://doi.org/10.5281/zenodo.11368812.
