# Supplementary material for: Changes in the distribution of elements in the liver and various brain regions in suicides from southeastern Poland
Source: Sci Rep. 2025 May 29;15:18946. doi: 10.1038/s41598-025-03283-2 (PMC12123043; doi:10.1038/s41598-025-03283-2)
Supplement: Supplementary file 1 — Supplementary Material 1 [file 41598_2025_3283_MOESM1_ESM.pdf]

Figure S1. The validation protocol of ICP-MS measurements. The report includes validation parameters (background equivalent concentration-BEC, detection limit-DL, internal standard-ISTD, calibration equation with correlation coefficient-R) and individual curves for each examined element.

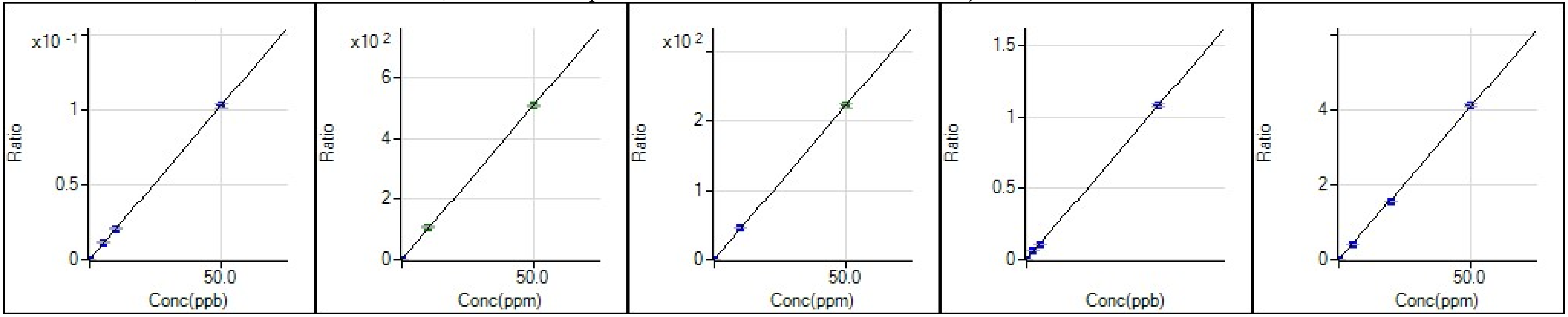

9 Be [No Gas]
ISTD: 45 Sc
y = 2.057E-3 x + 6.955E-6
R 0.9999
DL 0.002158
BEC 0.003382

23 Na [He]
ISTD: 45 Sc
y = 1.019E1 x + 6.326E-1
R 0.9999
DL 0.005241
BEC 0.0621

24 Mg [He]
ISTD: 45 Sc
y = 4.444E0 x + 5.516E-3
R 0.9999
DL 0.0004749
BEC 0.001241

27 Al [He]
ISTD: 45 Sc
y = 1.077E-3 x + 2.405E-3
R 1.0000
DL 0.2184
BEC 2.233

31 P [He]
ISTD: 45 Sc
y = 8.154E-2 x + 5.948E-4
R 0.9997
DL 0.007287
BEC 0.007294

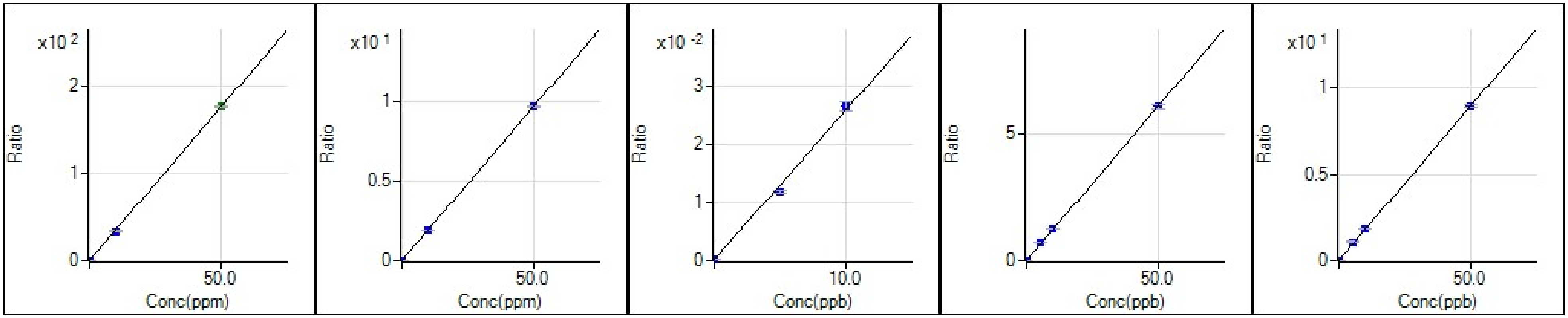

39 K [He]
ISTD: 45 Sc
y = 3.532E0 x + 3.421E-1
R 1.0000
DL 0.003932
BEC 0.09686

44 Ca [He]
ISTD: 45 Sc
y = 1.930E-1 x + 5.666E-3
R 1.0000
DL 0.003975
BEC 0.02936

47 Ti [He]
ISTD: 45 Sc
y = 2.572E-3 x + 1.748E-4
R 0.9979
DL 0.07481
BEC 0.06799

51 V [He]
ISTD: 45 Sc
y = 1.209E-1 x + 2.800E-4
R 0.9999
DL 0.004829
BEC 0.002317

52 Cr [He]
ISTD: 45 Sc
y = 1.785E-1 x + 1.599E-2
R 0.9998
DL 0.032
BEC 0.08958

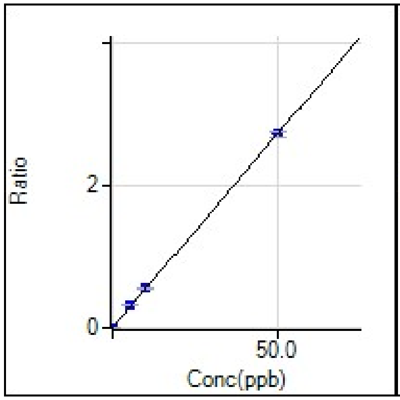

55 Mn [He]
ISTD: 45 Sc
y = 5.459E-2 x + 2.652E-3

R	0.9998
DL	0.007521
BEC	0.04857

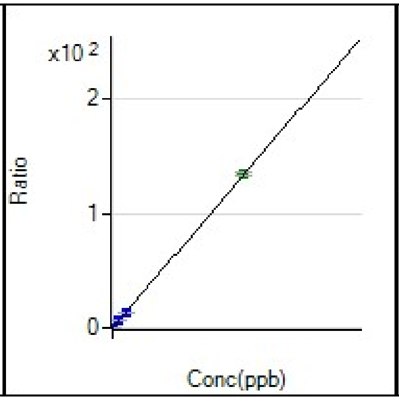

56 Fe [He]
ISTD: 45 Sc
y = 1.345E-1 x + 1.234E-1

R	1.0000
DL	0.05304
BEC	0.9174

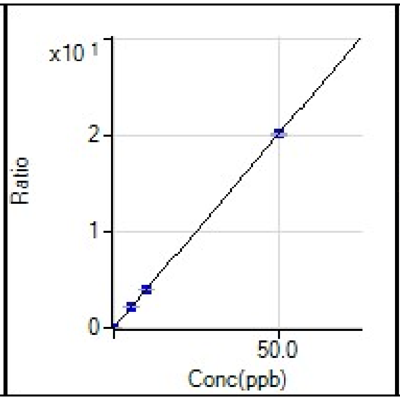

59 Co [He]
ISTD: 45 Sc
y = 4.025E-1 x + 1.069E-3

R	0.9999
DL	0.001483
BEC	0.002655

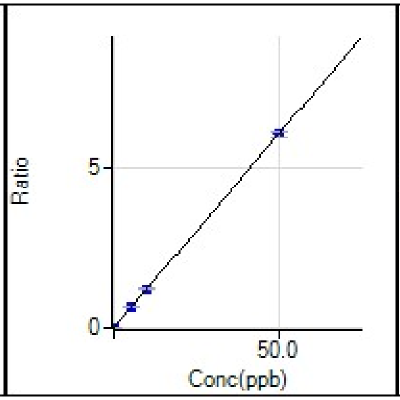

60 Ni [He]
ISTD: 45 Sc
y = 1.207E-1 x + 1.303E-2

R	0.9999
DL	0.02193
BEC	0.108

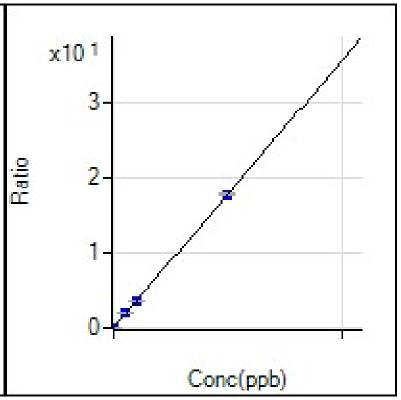

63 Cu [He]
ISTD: 45 Sc
y = 3.559E-1 x + 3.283E-2

R	0.9999
DL	0.02035
BEC	0.09225

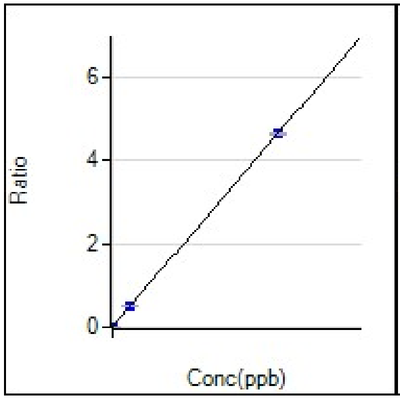

66 Zn [He]
ISTD: 89 Y
y = 4.645E-3 x + 1.122E-2

R	1.0000
DL	0.2187
BEC	2.417

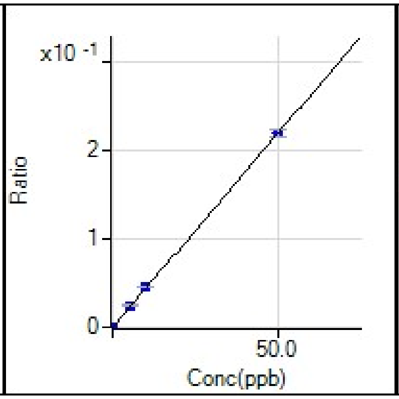

71 Ga [He]
ISTD: 89 Y
y = 4.394E-3 x

R	0.9999
DL	0
BEC	0

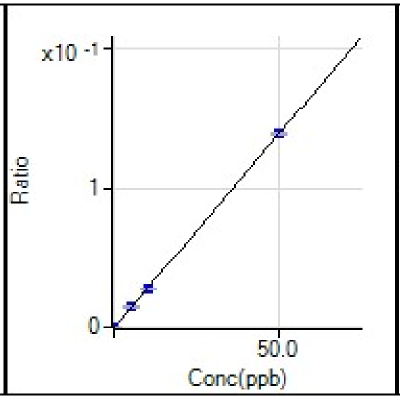

75 As [He]
ISTD: 89 Y
y = 2.785E-3 x + 3.768E-5

R	1.0000
DL	0.02058
BEC	0.01353

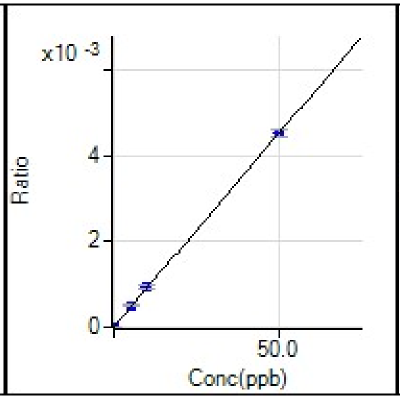

78 Se [He]
ISTD: 89 Y
y = 9.022E-5 x + 7.234E-6

R	0.9999
DL	0.1176
BEC	0.08019

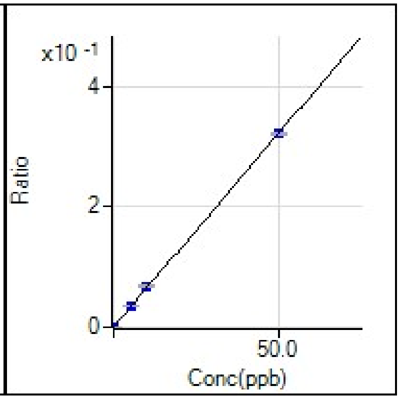

85 Rb [He]
ISTD: 89 Y
y = 6.442E-3 x + 2.060E-4

R	0.9999
DL	0.01087
BEC	0.03198

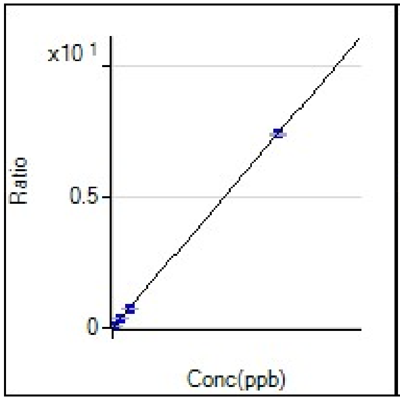

88 Sr [He]
ISTD: 89 Y
y = 7.402E-3 x + 1.694E-3

R	1.0000
DL	0.09661
BEC	0.2288

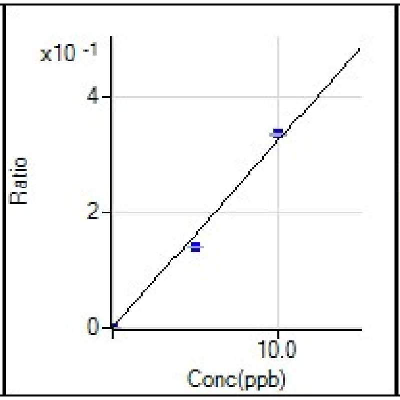

90 Zr [He]
ISTD: 89 Y
y = 3.222E-2 x + 9.700E-4

R	0.9950
DL	0.03327
BEC	0.0301

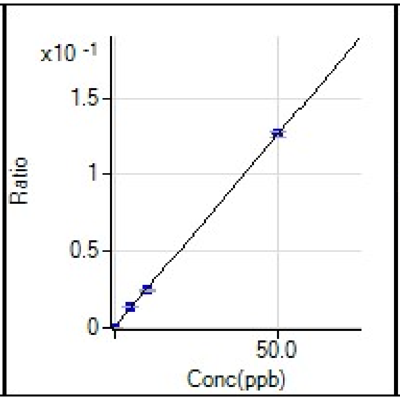

75 -> 91 As [O2]
ISTD: 89 -> 105 Y
y = 2.526E-3 x + 4.927E-5

R	0.9999
DL	0.005205
BEC	0.01951

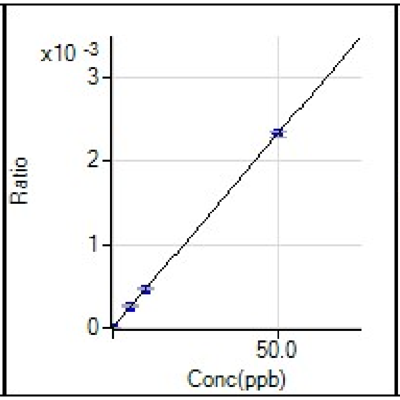

78 -> 94 Se [O2]
ISTD: 89 -> 105 Y
y = 4.629E-5 x + 5.542E-6

R	0.9999
DL	0.02681
BEC	0.1197

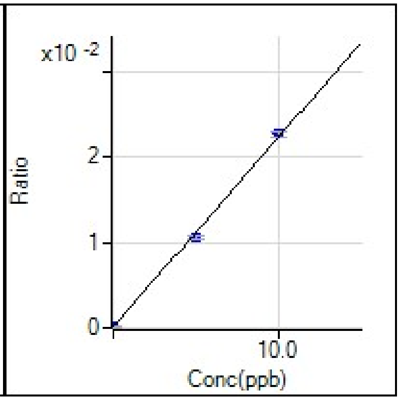

95 Mo [He]
ISTD: 89 Y
y = 2.220E-3 x + 1.251E-4

R	0.9990
DL	0.06203
BEC	0.05633

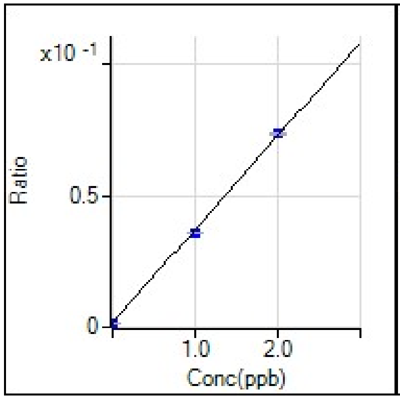

105 Pd [He]
ISTD: 89 Y
y = 3.556E-2 x + 1.702E-3

R	0.9995
DL	0.01726
BEC	0.04786

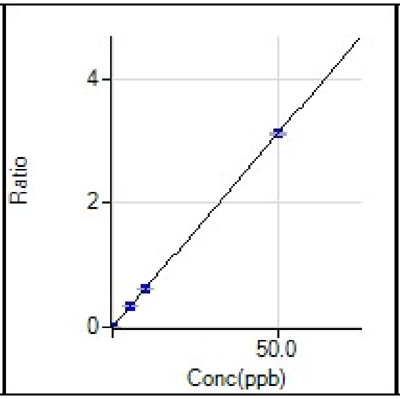

107 Ag [He]
ISTD: 89 Y
y = 6.242E-2 x + 6.733E-4

R	0.9999
DL	0.003667
BEC	0.01079

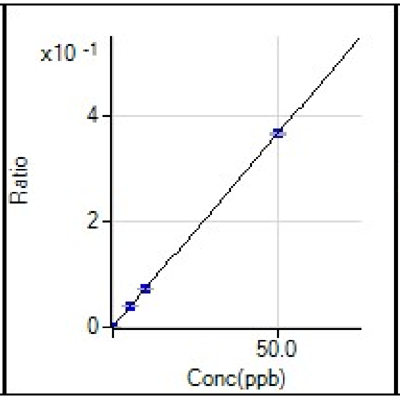

111 Cd [He]
ISTD: 89 Y
y = 7.294E-3 x + 2.418E-5

R	1.0000
DL	0.003736
BEC	0.003315

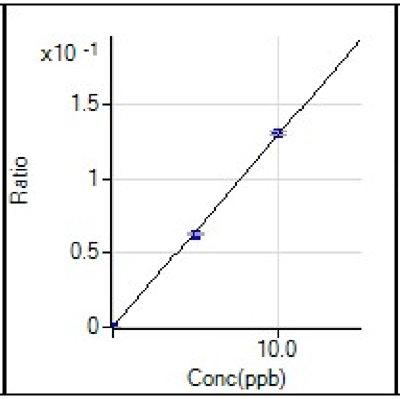

118 Sn [He]
ISTD: 89 Y
y = 1.292E-2 x + 1.653E-4

R	0.9997
DL	0.006777
BEC	0.0128

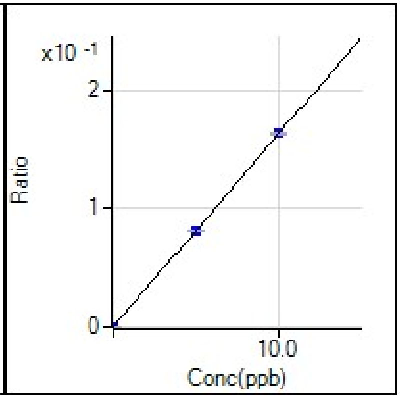

121 Sb [He]
ISTD: 89 Y
y = 1.627E-2 x + 3.873E-4

R	1.0000
DL	0.01147
BEC	0.0238

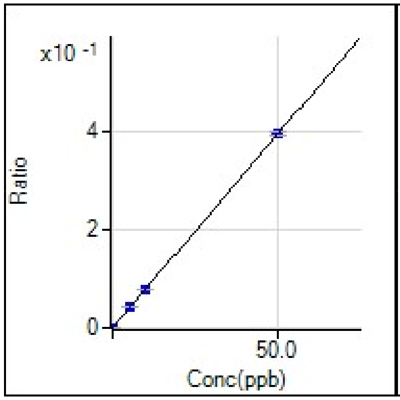

133 Cs [He]
ISTD: 175 Lu
$y = 7.923E-3 x + 3.765E-5$

R	1.0000
DL	0.007771
BEC	0.004753

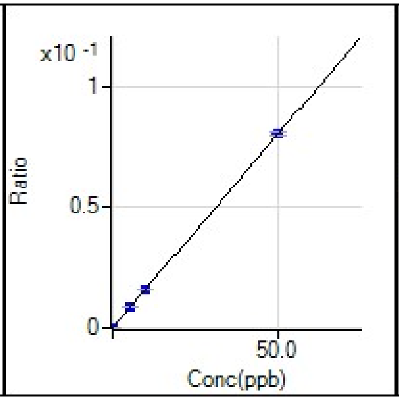

137 Ba [He]
ISTD: 175 Lu
$y = 1.605E-3 x + 4.705E-5$

R	0.9999
DL	0.02638
BEC	0.02931

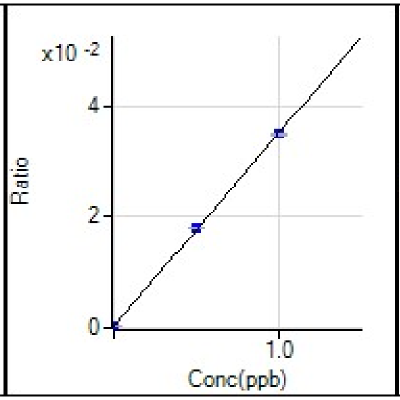

139 La [He]
ISTD: 175 Lu
$y = 3.482E-2 x + 2.903E-4$

R	0.9999
DL	0.00423
BEC	0.008338

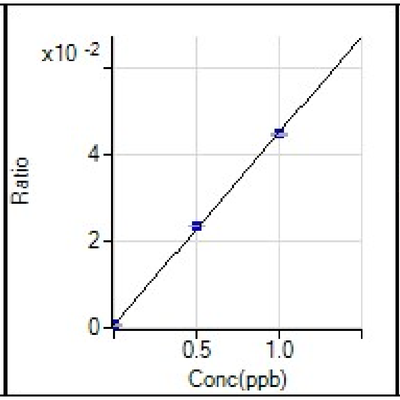

140 Ce [He]
ISTD: 175 Lu
$y = 4.456E-2 x + 6.728E-4$

R	0.9998
DL	0.002787
BEC	0.0151

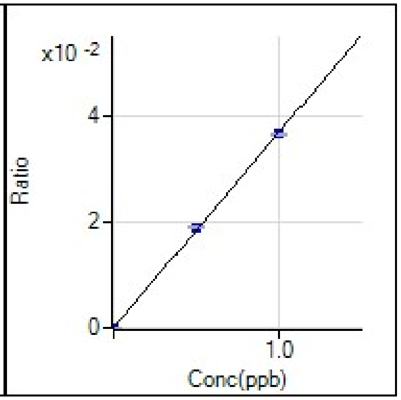

141 Pr [He]
ISTD: 175 Lu
$y = 3.700E-2 x + 3.914E-5$

R	0.9998
DL	0.001947
BEC	0.001058

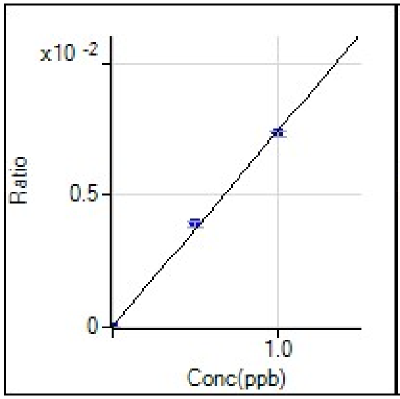

146 Nd [He]
ISTD: 175 Lu
$y = 7.404E-3 x + 1.098E-5$

R	0.9993
DL	0.001089
BEC	0.001482

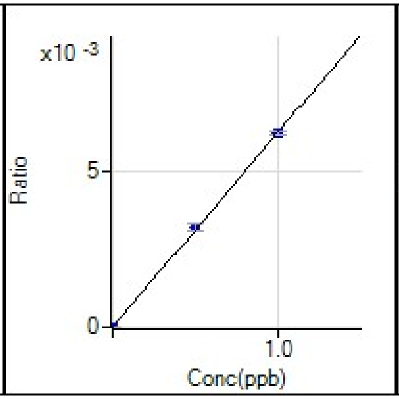

147 Sm [He]
ISTD: 175 Lu
$y = 6.241E-3 x + 1.563E-6$

R	0.9998
DL	0.001301
BEC	0.0002504

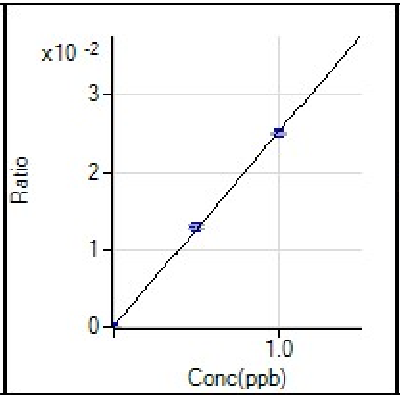

153 Eu [He]
ISTD: 175 Lu
$y = 2.519E-2 x + 6.252E-6$

R	0.9998
DL	0.0008539
BEC	0.0002482

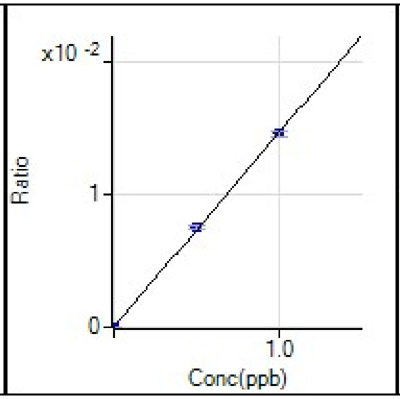

157 Gd [He]
ISTD: 175 Lu
$y = 1.466E-2 x + 1.416E-5$

R	0.9999
DL	0.002574
BEC	0.0009665

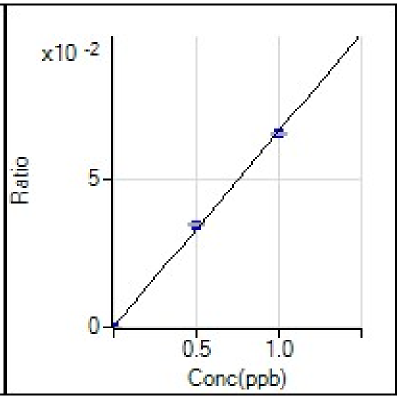

159 Tb [He]
ISTD: 175 Lu
$y = 6.621E-2 x + 3.442E-5$

R	0.9994
DL	0.0007997
BEC	0.0005199

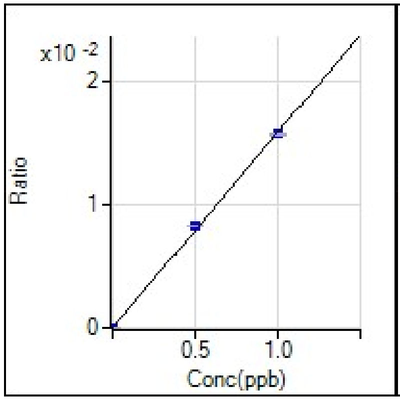

163 Dy [He]

ISTD: 175 Lu

y = 1.592E-2 x + 1.255E-5

R	0.9996
DL	0.0005199
BEC	0.0007887

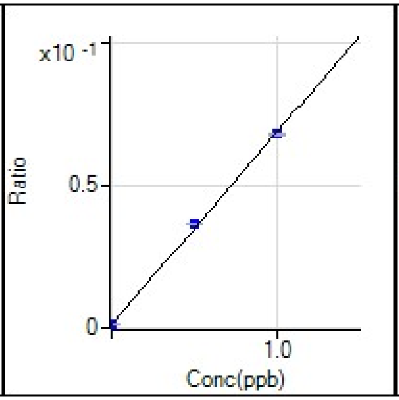

165 Ho [He]

ISTD: 175 Lu

y = 6.731E-2 x + 1.240E-3

R	0.9995
DL	0.001658
BEC	0.01843

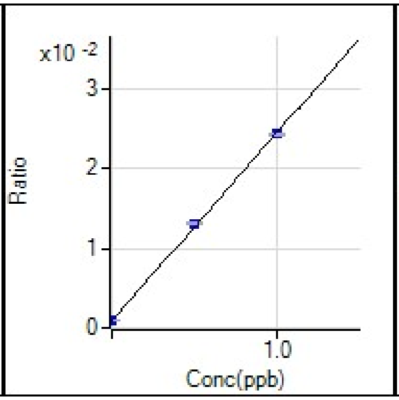

166 Er [He]

ISTD: 175 Lu

y = 2.359E-2 x + 9.359E-4

R	0.9997
DL	0.008019
BEC	0.03968

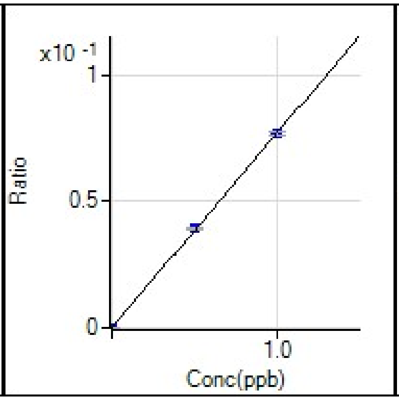

169 Tm [He]

ISTD: 175 Lu

y = 7.710E-2 x + 1.427E-4

R	0.9999
DL	8.716E-05
BEC	0.001851

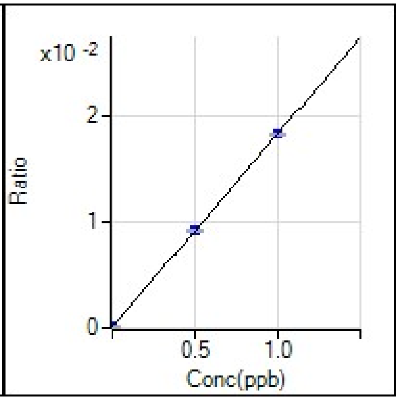

172 Yb [He]

ISTD: 175 Lu

y = 1.816E-2 x + 9.248E-5

R	1.0000
DL	0.002897
BEC	0.005093

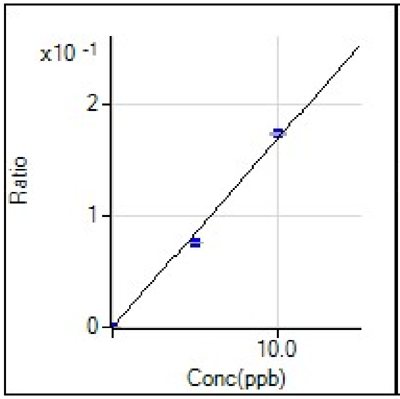

178 Hf [He]

ISTD: 175 Lu

y = 1.687E-2 x + 2.666E-5

R	0.9972
DL	0.001275
BEC	0.00158

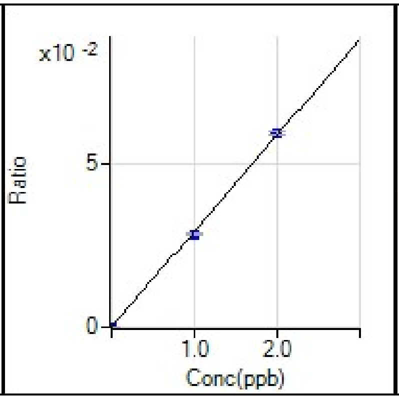

195 Pt [He]

ISTD: 175 Lu

y = 2.918E-2 x + 1.026E-4

R	0.9997
DL	0.0006133
BEC	0.003514

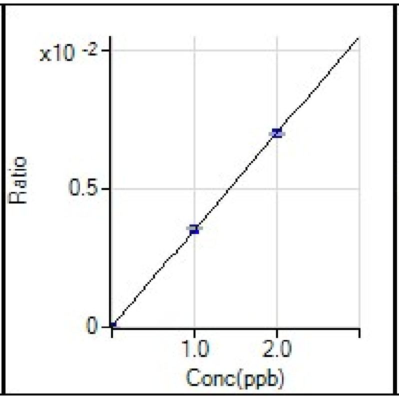

201 Hg [He]

ISTD: 175 Lu

y = 3.505E-3 x + 1.294E-5

R	0.9999
DL	0.001767
BEC	0.003691

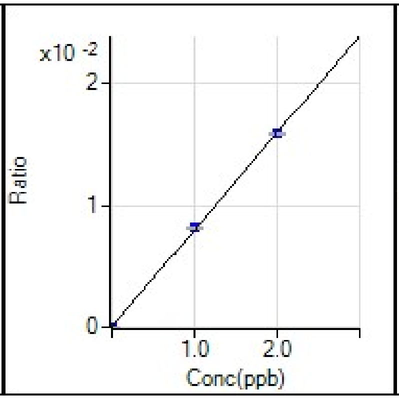

202 Hg [He]

ISTD: 175 Lu

y = 7.970E-3 x + 3.126E-5

R	0.9999
DL	0.002258
BEC	0.003923

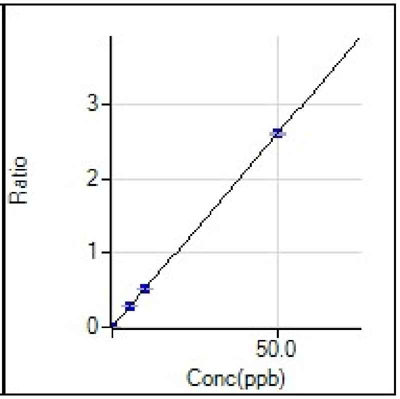

205 Tl [He]

ISTD: 175 Lu

y = 5.213E-2 x + 2.401E-4

R	1.0000
DL	0.003745
BEC	0.004606

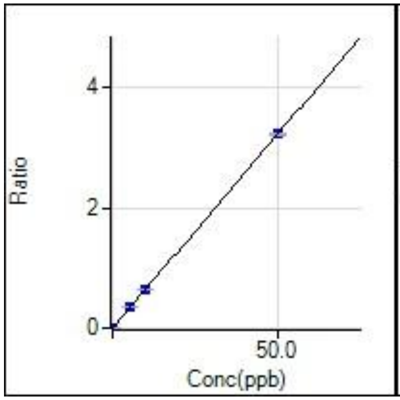

208 Pb [He]
ISTD: 175 Lu
y = 6.466E-2 x + 1.854E-3

R	1.0000
DL	0.009429
BEC	0.02868

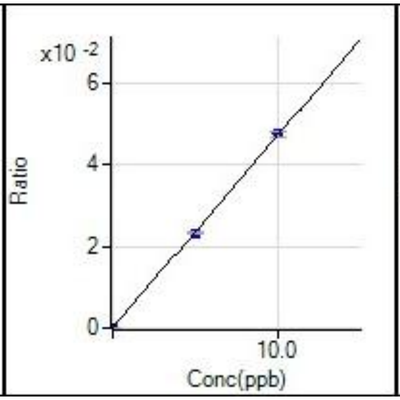

209 Bi [He]
ISTD: 175 Lu
y = 4.706E-3 x + 8.943E-5

R	0.9999
DL	0.008295
BEC	0.019

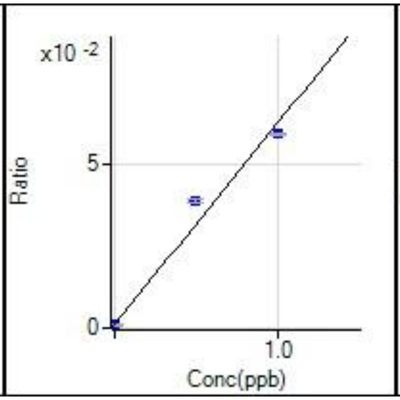

232 Th [He]
ISTD: 175 Lu
y = 6.202E-2 x + 9.470E-4

R	0.9849
DL	0.006537
BEC	0.01527

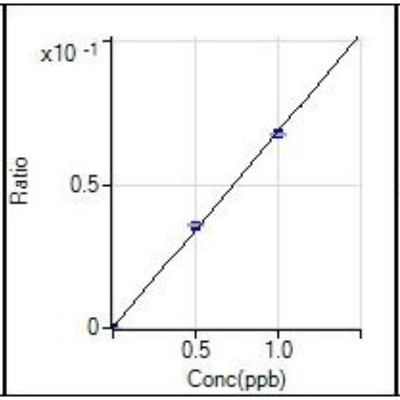

238 U [He]
ISTD: 175 Lu
y = 6.851E-2 x + 9.416E-5

R	0.9994
DL	0.001166
BEC	0.001374

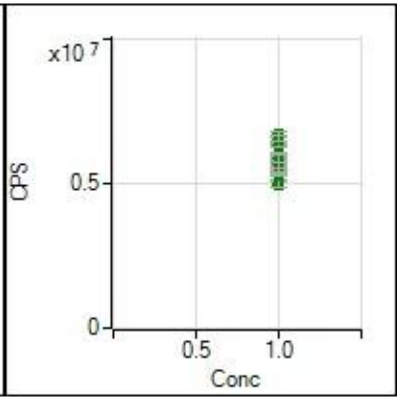

45 Sc [No Gas]
ISTD: ---
Excluded
R
DL
BEC

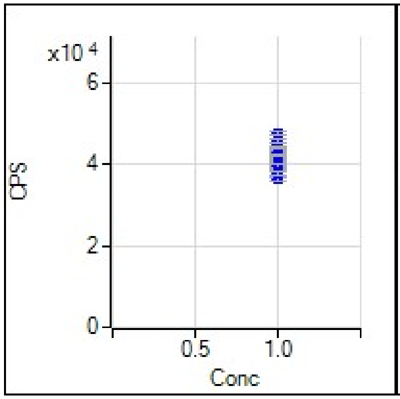

45 Sc [He]
ISTD: ---
Excluded
R
DL
BEC

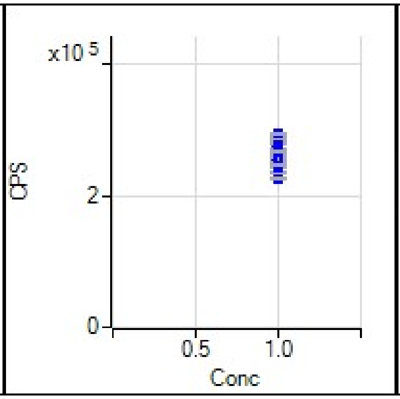

89 Y [He]
ISTD: ---
Excluded
R
DL
BEC

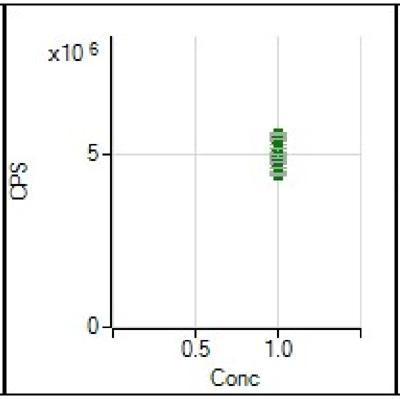

89 -> 105 Y [O2]
ISTD: ---
Excluded
R
DL
BEC

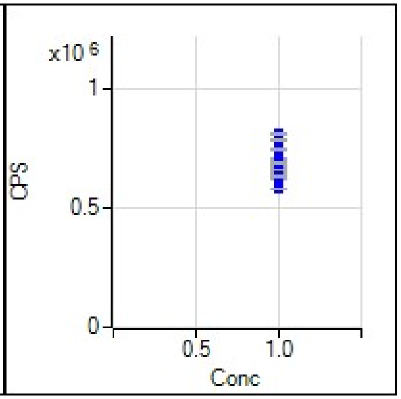

175 Lu [He]
ISTD: ---
Excluded
R
DL
BEC
