# Supplementary material for: Changes in the distribution of elements in the liver and various brain regions in suicides from southeastern Poland
Source: Sci Rep. 2025 May 29;15:18946. doi: 10.1038/s41598-025-03283-2 (PMC12123043; doi:10.1038/s41598-025-03283-2)
Supplement: Supplementary file 2 — Supplementary Material 2 [file 41598_2025_3283_MOESM2_ESM.pdf]

Table S1. Descriptive statistics (1-12) and comparative analysis (I-XII) for ICP-MS Elemental Measurements.

Legend for Table S1:

Hg denotes ^{201}Hg, Hg_2 denotes ^{202}Hg; As denotes ^{75}As, As_2 denotes $^{75\rightarrow 91}As$, Se denotes ^{78}Se, Se_2 denotes $^{78\rightarrow 94}Se$

1.frontal pole (A)

Group	variable	n	min	mean	median	max	sd
control	Ag	24	0.000	3.568	2.489	11.471	3.767
suicide	Ag	14	0.000	8.515	7.673	25.648	8.122
control	Al	24	19.915	342.311	206.231	1257.422	358.056
suicide	Al	14	19.782	239.022	199.443	888.156	252.557
control	As	24	0.000	0.000	0.000	0.000	0.000
suicide	As	14	0.000	0.000	0.000	0.000	0.000
control	As2	24	0.000	0.000	0.000	0.000	0.000
suicide	As2	14	0.000	0.000	0.000	0.000	0.000
control	Ba	24	2.141	8.725	6.365	22.569	6.022
suicide	Ba	14	2.544	7.340	6.777	14.203	3.950
control	Be	24	0.000	0.039	0.000	0.624	0.136
suicide	Be	14	0.000	0.015	0.000	0.149	0.042
control	Bi	24	0.000	0.974	0.867	2.867	0.798
suicide	Bi	14	0.000	13.113	0.645	169.258	44.969
control	Ca	24	33.216	142.189	105.360	679.448	125.768
suicide	Ca	14	48.165	119.836	109.150	248.443	58.212
control	Cd	24	2.423	40.086	21.281	295.251	60.469
suicide	Cd	14	3.932	42.045	20.254	151.460	47.062
control	Ce	24	0.000	0.759	0.211	9.478	1.984
suicide	Ce	14	0.000	0.679	0.214	5.578	1.460

Group	variable	n	min	mean	median	max	sd
control	Co	24	0.000	2.670	1.876	16.810	3.532
suicide	Co	14	0.000	2.267	1.855	8.184	2.431
control	Cr	24	0.000	34.280	11.647	188.635	49.520
suicide	Cr	14	0.000	32.565	17.219	94.163	32.629
control	Cs	24	1.076	3.536	3.480	6.287	1.714
suicide	Cs	14	1.678	4.207	3.569	9.653	2.277
control	Cu	24	2046.547	3774.692	3629.378	6768.235	1017.195
suicide	Cu	14	2259.469	4226.078	4125.214	6008.525	1055.734
control	Dy	24	0.000	0.018	0.017	0.058	0.016
suicide	Dy	14	0.000	0.014	0.010	0.033	0.012
control	Er	24	0.000	0.058	0.062	0.186	0.050
suicide	Er	14	0.000	0.050	0.052	0.167	0.051
control	Eu	24	0.000	0.010	0.008	0.043	0.009
suicide	Eu	14	0.000	0.011	0.008	0.032	0.009
control	Fe	24	13327.135	47271.695	40281.017	108615.435	21546.060
suicide	Fe	14	19111.913	56593.928	47948.162	136740.547	29596.330
control	Ga	24	0.000	0.157	0.011	1.050	0.257
suicide	Ga	14	0.000	0.234	0.063	0.763	0.300
control	Gd	24	0.000	0.053	0.028	0.512	0.104
suicide	Gd	14	0.000	0.067	0.040	0.336	0.089
control	Hf	24	0.000	0.003	0.000	0.036	0.009
suicide	Hf	14	0.000	0.041	0.000	0.432	0.117
control	Hg	24	1.284	4.153	3.417	19.416	3.681
suicide	Hg	14	0.596	4.387	3.285	19.168	4.611
control	Hg2	24	1.326	4.185	3.565	19.484	3.709
suicide	Hg2	14	0.475	4.391	3.335	19.116	4.601

Group	variable	n	min	mean	median	max	sd
control	Ho	24	0.000	0.012	0.000	0.055	0.018
suicide	Ho	14	0.000	0.009	0.000	0.044	0.016
control	K	24	1389.592	2270.393	2300.885	3814.142	610.458
suicide	K	14	1393.570	2416.781	2352.850	4547.802	748.211
control	La	24	0.000	0.501	0.120	6.743	1.418
suicide	La	14	0.008	0.472	0.109	4.148	1.105
control	Mg	24	44.964	89.994	81.464	202.142	39.672
suicide	Mg	14	40.626	91.834	87.907	137.226	27.714
control	Mn	24	95.959	193.244	185.656	335.411	52.973
suicide	Mn	14	115.826	199.101	200.259	285.525	50.779
control	Mo	24	0.000	228.334	170.276	1070.888	244.665
suicide	Mo	14	0.000	213.046	153.106	611.495	181.784
control	Na	24	814.713	2218.458	2206.590	3536.112	737.129
suicide	Na	14	988.548	2171.281	2118.007	3402.803	566.660
control	Nd	24	0.000	0.131	0.071	0.750	0.186
suicide	Nd	14	0.004	0.104	0.088	0.301	0.081
control	Ni	24	0.000	7.194	3.961	26.753	7.742
suicide	Ni	14	0.000	4.249	4.734	9.212	2.844
control	P	24	1187.215	1973.340	1953.879	3405.832	599.481
suicide	P	14	1142.625	2041.182	2047.325	3185.247	512.093
control	Pb	24	0.000	4.126	2.676	18.365	4.799
suicide	Pb	14	0.000	2.339	1.942	8.168	2.515
control	Pd	24	0.000	0.119	0.060	0.547	0.160
suicide	Pd	14	0.000	0.070	0.012	0.312	0.101
control	Pr	24	0.003	0.039	0.021	0.312	0.066
suicide	Pr	14	0.000	0.036	0.025	0.153	0.042

Group	variable	n	min	mean	median	max	sd
control	Pt	24	0.004	0.715	0.023	16.062	3.270
suicide	Pt	14	0.006	0.066	0.036	0.207	0.072
control	Rb	24	694.425	1607.640	1544.900	2685.769	595.583
suicide	Rb	14	1027.904	1794.419	1677.626	4049.433	779.213
control	Sb	24	0.000	0.058	0.000	0.707	0.161
suicide	Sb	14	0.000	0.067	0.000	0.849	0.226
control	Se	24	84.925	141.515	150.126	197.054	29.267
suicide	Se	14	55.621	144.366	147.824	218.192	38.457
control	Se2	24	66.081	124.678	125.743	191.684	28.265
suicide	Se2	14	51.882	125.197	125.793	217.156	39.511
control	Sm	24	0.000	0.017	0.012	0.065	0.020
suicide	Sm	14	0.000	0.021	0.010	0.094	0.028
control	Sn	24	0.229	1.601	1.453	3.516	0.859
suicide	Sn	14	0.000	1.318	1.041	4.195	1.186
control	Sr	24	92.995	355.282	231.666	1735.257	344.653
suicide	Sr	14	98.357	228.653	231.095	453.653	107.705
control	Tb	24	0.000	0.008	0.007	0.026	0.006
suicide	Tb	14	0.000	0.007	0.006	0.016	0.005
control	Th	24	0.000	0.000	0.000	0.000	0.000
suicide	Th	14	0.000	0.000	0.000	0.000	0.000
control	Ti	24	8.135	30.754	25.277	75.533	16.345
suicide	Ti	14	12.866	24.679	22.114	42.860	9.442
control	Tl	24	0.000	0.000	0.000	0.000	0.000
suicide	Tl	14	0.000	0.000	0.000	0.000	0.000
control	Tm	24	0.000	0.009	0.007	0.024	0.007
suicide	Tm	14	0.000	0.010	0.011	0.020	0.006

Group	variable	n	min	mean	median	max	sd
control	U	24	0.000	0.071	0.000	0.716	0.159
suicide	U	14	0.000	0.018	0.000	0.084	0.028
control	V	24	0.267	2.938	2.821	7.989	1.679
suicide	V	14	0.469	3.276	2.135	9.679	2.785
control	Yb	24	0.000	0.020	0.014	0.091	0.023
suicide	Yb	14	0.000	0.010	0.001	0.041	0.015
control	Zn	24	6285.875	16595.901	14444.711	31296.973	7088.374
suicide	Zn	14	8502.579	17663.073	15466.799	36129.549	7180.047
control	Zr	24	0.000	2.070	0.618	34.489	6.922
suicide	Zr	14	0.000	0.808	0.611	2.033	0.676

I. frontal pole (A)

Element	Control mean	Suicide mean	Dunn statistic	p
Ag	17.083	23.643	1.761	0.078
Al	20.958	17.000	−1.059	0.290
Ba	20.167	18.357	−0.484	0.628
Be	19.417	19.643	0.103	0.918
Bi	19.958	18.714	−0.333	0.739
Ca	19.792	19.000	−0.212	0.832
Cd	19.500	19.500	0.000	1.000
Ce	19.521	19.464	−0.015	0.988
Co	19.708	19.143	−0.153	0.878
Cr	19.312	19.821	0.136	0.892
Cs	18.458	21.286	0.757	0.449

Cu	17.500	22.929	1.453	0.146
Dy	19.958	18.714	−0.334	0.738
Er	20.250	18.214	−0.551	0.581
Eu	19.292	19.857	0.151	0.880
Fe	18.000	22.071	1.089	0.276
Ga	18.750	20.786	0.571	0.568
Gd	17.958	22.143	1.120	0.263
Hf	18.083	21.929	1.621	0.105
Hg	19.708	19.143	−0.151	0.880
Hg2	19.667	19.214	−0.121	0.904
Ho	20.083	18.500	−0.480	0.631
K	18.958	20.429	0.393	0.694
La	19.625	19.286	−0.091	0.928
Mg	18.542	21.143	0.696	0.486
Mn	18.542	21.143	0.696	0.486
Mo	19.333	19.786	0.121	0.904
Na	19.833	18.929	−0.242	0.809
Nd	18.792	20.714	0.514	0.607
Ni	20.062	18.536	−0.409	0.683
P	18.750	20.786	0.545	0.586
Pb	20.500	17.786	−0.735	0.462
Pd	20.979	16.964	−1.102	0.270

Pr	19.125	20.143	0.272	0.785
Pt	18.542	21.143	0.696	0.486
Rb	18.958	20.429	0.393	0.694
Sb	20.375	18.000	−0.852	0.394
Se	19.417	19.643	0.061	0.952
Se2	19.500	19.500	0.000	1.000
Sm	19.458	19.571	0.031	0.975
Sn	21.167	16.643	−1.210	0.226
Sr	20.958	17.000	−1.059	0.290
Tb	20.208	18.286	−0.515	0.607
Ti	21.000	16.929	−1.089	0.276
Tm	18.708	20.857	0.578	0.564
U	20.167	18.357	−0.531	0.595
V	19.917	18.786	−0.303	0.762
Yb	21.708	15.714	−1.637	0.102
Zn	18.708	20.857	0.575	0.565
Zr	19.250	19.929	0.182	0.856

2.precentral gyrus (B)

Group	variable	n	min	mean	median	max	sd
control	Ag	22	0.000	4.916	2.778	13.824	4.372
suicide	Ag	13	0.000	11.265	7.867	36.322	11.966

Group	variable	n	min	mean	median	max	sd
control	Al	22	0.000	279.374	243.521	831.725	228.634
suicide	Al	13	0.000	469.255	226.760	1883.312	519.387
control	As	22	0.000	0.000	0.000	0.000	0.000
suicide	As	13	0.000	0.000	0.000	0.000	0.000
control	As2	22	0.000	0.000	0.000	0.000	0.000
suicide	As2	13	0.000	0.000	0.000	0.000	0.000
control	Ba	22	0.982	6.844	6.996	13.807	3.484
suicide	Ba	13	1.491	7.605	5.922	26.515	6.335
control	Be	22	0.000	0.044	0.000	0.830	0.177
suicide	Be	13	0.000	0.001	0.000	0.013	0.004
control	Bi	22	0.000	3.794	0.920	64.873	13.663
suicide	Bi	13	0.000	0.902	0.480	3.507	1.163
control	Ca	22	23.792	86.218	76.952	165.900	31.376
suicide	Ca	13	45.839	92.957	88.588	146.272	29.491
control	Cd	22	3.961	40.133	23.675	207.401	50.562
suicide	Cd	13	5.175	19.034	9.958	48.269	15.816
control	Ce	22	0.000	0.519	0.128	5.844	1.233
suicide	Ce	13	0.040	0.192	0.127	0.794	0.195
control	Co	22	0.000	2.771	2.162	21.931	4.493
suicide	Co	13	0.000	1.644	1.946	3.481	1.454
control	Cr	22	0.000	25.414	9.311	122.022	36.504
suicide	Cr	13	0.000	123.834	14.084	1412.438	387.489

control	Cs	22	1.078	4.126	4.189	7.303	1.709
suicide	Cs	13	2.311	4.845	5.096	8.595	1.720
control	Cu	22	1057.645	4080.247	4286.100	6650.593	1337.622
suicide	Cu	13	2169.173	5229.358	4629.397	9086.587	1921.291
control	Dy	22	0.000	0.014	0.011	0.048	0.014
suicide	Dy	13	0.000	0.011	0.005	0.042	0.013
control	Er	22	0.000	0.046	0.016	0.232	0.064
suicide	Er	13	0.000	0.043	0.003	0.285	0.087
control	Eu	22	0.000	0.009	0.008	0.026	0.008
suicide	Eu	13	0.000	0.013	0.009	0.042	0.012
control	Fe	22	27337.866	73820.683	68551.018	147687.886	26737.923
suicide	Fe	13	45227.278	92192.499	85315.509	155129.594	38286.208
control	Ga	22	0.000	0.122	0.010	0.554	0.181
suicide	Ga	13	0.000	0.059	0.000	0.384	0.113
control	Gd	22	0.000	0.077	0.020	0.670	0.154
suicide	Gd	13	0.000	0.052	0.034	0.152	0.053
control	Hf	22	0.000	0.147	0.000	1.611	0.469
suicide	Hf	13	0.000	0.010	0.000	0.129	0.036
control	Hg	22	0.306	4.213	3.085	15.488	3.433
suicide	Hg	13	0.586	3.765	3.111	7.615	2.074
control	Hg2	22	0.341	4.153	2.981	15.289	3.413
suicide	Hg2	13	0.555	3.814	3.381	7.671	2.062

control	Ho	22	0.000	0.009	0.000	0.046	0.016
suicide	Ho	13	0.000	0.022	0.000	0.206	0.057
control	K	22	552.810	2205.462	2285.836	3015.041	497.433
suicide	K	13	1710.315	2462.314	2424.618	3737.492	569.313
control	La	22	0.000	0.377	0.093	4.248	0.900
suicide	La	13	0.000	0.131	0.112	0.394	0.106
control	Mg	22	17.121	89.528	83.700	170.954	33.716
suicide	Mg	13	63.618	103.709	102.939	183.104	33.078
control	Mn	22	47.862	216.492	226.604	271.606	47.236
suicide	Mn	13	166.646	250.898	220.352	496.320	92.977
control	Mo	22	0.000	197.929	145.707	685.443	178.677
suicide	Mo	13	0.000	149.288	142.470	406.703	124.863
control	Na	22	680.393	2033.295	2138.923	2678.142	509.585
suicide	Na	13	1640.675	2507.126	2349.095	3919.262	615.333
control	Nd	22	0.005	0.081	0.059	0.250	0.060
suicide	Nd	13	0.000	0.080	0.063	0.169	0.053
control	Ni	22	0.000	6.573	4.352	22.505	6.551
suicide	Ni	13	0.000	6.406	5.218	18.958	6.231
control	P	22	669.227	2193.105	2189.725	2958.899	511.949
suicide	P	13	1791.252	2493.205	2318.192	3668.037	510.743
control	Pb	22	0.000	2.937	2.640	10.647	2.749
suicide	Pb	13	0.000	1.580	1.344	4.934	1.571

control	Pd	22	0.000	0.091	0.069	0.289	0.089
suicide	Pd	13	0.000	0.085	0.017	0.483	0.142
control	Pr	22	0.000	0.030	0.027	0.101	0.028
suicide	Pr	13	0.000	0.019	0.010	0.094	0.026
control	Pt	22	0.000	0.049	0.047	0.144	0.038
suicide	Pt	13	0.000	0.078	0.055	0.289	0.084
control	Rb	22	458.228	1741.475	1770.319	2401.928	551.573
suicide	Rb	13	1226.570	1906.678	1899.634	2656.642	418.108
control	Sb	22	0.000	0.090	0.000	0.961	0.235
suicide	Sb	13	0.000	0.009	0.000	0.120	0.033
control	Se	22	30.856	148.802	144.829	222.094	40.664
suicide	Se	13	110.361	160.038	155.791	257.481	43.108
control	Se2	22	31.345	135.658	131.951	195.388	37.270
suicide	Se2	13	93.650	142.501	136.782	240.015	44.050
control	Sm	22	0.000	0.022	0.017	0.085	0.022
suicide	Sm	13	0.000	0.038	0.036	0.138	0.038
control	Sn	22	0.000	2.164	1.620	11.620	2.564
suicide	Sn	13	0.000	2.078	1.615	7.741	2.103
control	Sr	22	73.288	272.706	214.201	894.540	213.592
suicide	Sr	13	82.607	297.670	201.454	894.529	242.701
control	Tb	22	0.000	0.006	0.007	0.013	0.004
suicide	Tb	13	0.000	0.008	0.006	0.017	0.006

control	Th	22	0.000	0.000	0.000	0.000	0.000
suicide	Th	13	0.000	0.000	0.000	0.000	0.000
control	Ti	22	10.650	31.553	29.578	58.324	12.410
suicide	Ti	13	13.732	32.731	27.841	66.995	15.861
control	Tl	22	0.000	0.002	0.000	0.042	0.009
suicide	Tl	13	0.000	0.000	0.000	0.000	0.000
control	Tm	22	0.000	0.009	0.008	0.039	0.010
suicide	Tm	13	0.000	0.012	0.009	0.035	0.011
control	U	22	0.000	0.036	0.003	0.205	0.059
suicide	U	13	0.000	0.024	0.000	0.163	0.051
control	V	22	0.460	3.256	2.803	8.507	1.976
suicide	V	13	0.902	3.203	2.852	11.492	2.897
control	Yb	22	0.000	0.019	0.005	0.114	0.028
suicide	Yb	13	0.000	0.021	0.012	0.070	0.025
control	Zn	22	5602.760	14059.485	12087.331	28513.962	5443.916
suicide	Zn	13	9864.452	15688.263	17204.054	24380.313	5165.453
control	Zr	22	0.000	7.026	0.509	76.564	20.514
suicide	Zr	13	0.000	0.953	0.575	3.071	1.017

II.precentral gyrus (B)

Element	Control mean	Suicide mean	Dunn statistic	p
Ag	16.273	20.923	1.298	0.194

Al	16.932	19.808	0.802	0.422
Ba	18.682	16.846	−0.512	0.609
Be	18.455	17.231	−0.618	0.537
Bi	19.682	15.154	−1.265	0.206
Ca	17.136	19.462	0.649	0.517
Cd	20.091	14.462	−1.570	0.116
Ce	18.409	17.308	−0.307	0.759
Co	18.773	16.692	−0.590	0.556
Cr	17.682	18.538	0.240	0.810
Cs	16.545	20.462	1.092	0.275
Cu	15.818	21.692	1.639	0.101
Dy	18.750	16.731	−0.567	0.571
Er	19.045	16.231	−0.806	0.420
Eu	17.364	19.077	0.478	0.632
Fe	16.318	20.846	1.263	0.207
Ga	19.455	15.538	−1.175	0.240
Gd	17.727	18.462	0.205	0.838
Hf	18.682	16.846	−0.841	0.400
Hg	17.955	18.077	0.034	0.973
Hg2	17.636	18.615	0.273	0.785
Ho	17.250	19.269	0.650	0.516
K	16.545	20.462	1.092	0.275

La	17.977	18.038	0.017	0.986
Mg	16.455	20.615	1.161	0.246
Mn	17.773	18.385	0.171	0.864
Mo	19.045	16.231	−0.786	0.432
Na	15.500	22.231	1.878	0.060
Nd	17.545	18.769	0.341	0.733
Ni	18.023	17.962	−0.017	0.986
P	16.182	21.077	1.366	0.172
Pb	19.818	14.923	−1.382	0.167
Pd	19.341	15.731	−1.019	0.308
Pr	19.955	14.692	−1.470	0.142
Pt	17.227	19.308	0.581	0.562
Rb	17.000	19.692	0.751	0.453
Sb	19.318	15.769	−1.417	0.157
Se	17.591	18.692	0.307	0.759
Se2	18.045	17.923	−0.034	0.973
Sm	16.341	20.808	1.249	0.212
Sn	17.909	18.154	0.068	0.945
Sr	17.727	18.462	0.205	0.838
Tb	17.864	18.231	0.102	0.918
Ti	18.227	17.615	−0.171	0.864
Tl	18.295	17.500	−0.769	0.442

Tm	16.727	20.154	0.964	0.335
U	19.205	15.962	−1.003	0.316
V	18.773	16.692	−0.580	0.562
Yb	17.545	18.769	0.345	0.730
Zn	17.318	19.154	0.512	0.609
Zr	18.091	17.846	−0.069	0.945

3.postcentral gyrus (C)

Group	variable	n	min	mean	median	max	sd
control	Ag	22	0.000	4.541	2.685	16.814	4.589
suicide	Ag	12	0.000	10.656	6.009	36.788	12.450
control	Al	22	7.476	217.885	164.835	911.108	211.280
suicide	Al	12	0.000	203.673	192.522	437.350	135.997
control	As	22	0.000	0.000	0.000	0.000	0.000
suicide	As	12	0.000	0.000	0.000	0.000	0.000
control	As2	22	0.000	0.000	0.000	0.000	0.000
suicide	As2	12	0.000	0.000	0.000	0.000	0.000
control	Ba	22	1.041	9.900	7.532	36.779	9.275
suicide	Ba	12	2.418	8.058	4.308	50.166	13.303
control	Be	22	0.000	0.017	0.000	0.196	0.049
suicide	Be	12	0.000	0.000	0.000	0.000	0.000
control	Bi	22	0.000	0.763	0.777	1.918	0.584

suicide	Bi	12	0.000	76.054	0.508	904.319	260.837
control	Ca	22	39.406	84.324	87.416	133.883	21.719
suicide	Ca	12	43.762	73.354	74.874	114.396	21.451
control	Cd	22	4.062	38.775	28.626	148.172	35.052
suicide	Cd	12	4.373	22.645	16.986	54.260	16.475
control	Ce	22	0.000	0.591	0.189	3.221	0.800
suicide	Ce	12	0.000	0.174	0.193	0.407	0.129
control	Co	22	0.000	1.771	1.931	5.204	1.526
suicide	Co	12	0.000	2.262	1.829	10.427	2.876
control	Cr	22	0.000	29.001	10.490	242.194	53.866
suicide	Cr	12	0.000	13.716	8.677	49.979	14.882
control	Cs	22	0.777	4.489	4.588	7.981	1.783
suicide	Cs	12	2.115	4.179	3.762	7.224	1.383
control	Cu	22	2657.471	4231.684	4298.092	6438.289	1012.523
suicide	Cu	12	3047.560	4602.739	4039.982	8394.929	1488.832
control	Dy	22	0.000	0.012	0.004	0.056	0.016
suicide	Dy	12	0.000	0.015	0.008	0.055	0.017
control	Er	22	0.000	0.050	0.026	0.193	0.060
suicide	Er	12	0.000	0.101	0.065	0.298	0.101
control	Eu	22	0.000	0.013	0.012	0.038	0.011
suicide	Eu	12	0.000	0.010	0.006	0.031	0.011
control	Fe	22	33584.215	70036.723	59937.863	134043.200	26956.473

suicide	Fe	12	38508.734	67803.141	62373.159	102049.058	21315.687
control	Ga	22	0.000	0.147	0.003	0.768	0.224
suicide	Ga	12	0.000	0.106	0.006	0.449	0.156
control	Gd	22	0.007	0.189	0.037	3.036	0.638
suicide	Gd	12	0.001	0.055	0.029	0.239	0.080
control	Hf	22	0.000	0.014	0.000	0.316	0.067
suicide	Hf	12	0.000	0.003	0.000	0.031	0.009
control	Hg	22	1.042	3.976	2.959	14.585	3.061
suicide	Hg	12	0.356	3.676	2.542	8.750	2.808
control	Hg2	22	1.053	3.989	2.916	14.687	3.084
suicide	Hg2	12	0.365	3.560	2.448	8.685	2.744
control	Ho	22	0.000	0.013	0.000	0.085	0.022
suicide	Ho	12	0.000	0.011	0.000	0.098	0.028
control	K	22	1598.395	2351.670	2359.717	3189.417	382.293
suicide	K	12	1794.825	2318.567	2209.181	3109.660	403.810
control	La	22	0.000	0.440	0.180	2.482	0.628
suicide	La	12	0.034	0.102	0.092	0.222	0.061
control	Mg	22	56.321	86.227	86.613	139.403	23.175
suicide	Mg	12	36.020	92.166	95.875	141.646	28.265
control	Mn	22	134.111	207.079	207.087	255.371	28.290
suicide	Mn	12	153.739	214.272	194.125	394.503	68.551
control	Mo	22	0.000	245.309	196.496	1192.699	259.339

suicide	Mo	12	0.000	144.718	151.593	339.788	94.077
control	Na	22	1442.030	2288.369	2292.051	3262.253	553.804
suicide	Na	12	1617.669	2322.401	2266.067	3106.209	502.614
control	Nd	22	0.000	0.105	0.072	0.385	0.096
suicide	Nd	12	0.009	0.053	0.060	0.091	0.030
control	Ni	22	0.000	5.831	4.231	17.787	5.029
suicide	Ni	12	0.000	14.824	4.138	93.718	26.860
control	P	22	1592.256	2228.340	2233.795	3226.420	466.027
suicide	P	12	1832.519	2405.757	2334.005	2997.262	432.896
control	Pb	22	0.000	2.946	2.617	8.098	2.404
suicide	Pb	12	0.000	1.147	0.857	3.318	1.221
control	Pd	22	0.000	0.092	0.051	0.325	0.101
suicide	Pd	12	0.000	0.038	0.000	0.326	0.097
control	Pr	22	0.005	0.039	0.028	0.174	0.037
suicide	Pr	12	0.000	0.018	0.010	0.062	0.021
control	Pt	22	0.006	0.072	0.032	0.467	0.099
suicide	Pt	12	0.005	0.022	0.017	0.081	0.021
control	Rb	22	531.926	1837.549	1811.749	2718.673	555.709
suicide	Rb	12	1288.402	1703.037	1605.302	2254.755	311.375
control	Sb	22	0.000	0.049	0.000	0.478	0.122
suicide	Sb	12	0.000	0.000	0.000	0.000	0.000
control	Se	22	94.871	143.154	140.439	199.367	30.250

suicide	Se	12	109.232	154.489	151.084	217.850	33.416
control	Se2	22	95.735	132.265	131.142	192.418	26.282
suicide	Se2	12	94.002	140.506	137.844	187.408	28.220
control	Sm	22	0.000	0.019	0.018	0.067	0.020
suicide	Sm	12	0.000	0.026	0.019	0.094	0.030
control	Sn	22	0.000	1.801	1.314	6.383	1.584
suicide	Sn	12	0.000	2.069	2.017	4.585	1.452
control	Sr	22	79.203	238.774	186.744	625.231	150.441
suicide	Sr	12	62.334	162.812	150.235	331.013	85.923
control	Tb	22	0.000	0.003	0.002	0.017	0.004
suicide	Tb	12	0.000	0.007	0.003	0.030	0.009
control	Th	22	0.000	0.000	0.000	0.000	0.000
suicide	Th	12	0.000	0.000	0.000	0.000	0.000
control	Ti	22	11.015	27.267	25.899	42.202	9.313
suicide	Ti	12	16.534	27.259	29.096	46.439	8.322
control	Tl	22	0.000	0.000	0.000	0.002	0.001
suicide	Tl	12	0.000	0.000	0.000	0.000	0.000
control	Tm	22	0.000	0.013	0.012	0.035	0.011
suicide	Tm	12	0.000	0.013	0.009	0.055	0.015
control	U	22	0.000	0.038	0.008	0.320	0.071
suicide	U	12	0.000	0.009	0.000	0.087	0.025
control	V	22	0.235	3.515	3.525	10.833	2.309

suicide	V	12	0.361	2.535	2.353	5.036	1.509
control	Yb	22	0.000	0.027	0.018	0.110	0.030
suicide	Yb	12	0.000	0.028	0.019	0.087	0.031
control	Zn	22	8827.282	16238.205	12895.361	29354.039	6393.213
suicide	Zn	12	9924.236	15288.367	13757.077	23800.755	5218.760
control	Zr	22	0.000	1.300	0.420	16.851	3.519
suicide	Zr	12	0.000	0.459	0.262	1.805	0.591

III. postcentral gyrus (C)

Element	Control mean	Suicide mean	Dunn statistic	p
Ag	15.977	20.292	1.208	0.227
Al	16.955	18.500	0.432	0.665
Ba	19.682	13.500	−1.730	0.084
Be	18.318	16.000	−1.318	0.187
Bi	17.614	17.292	−0.090	0.928
Ca	19.500	13.833	−1.586	0.113
Cd	19.318	14.167	−1.441	0.149
Ce	19.409	14.000	−1.514	0.130
Co	17.500	17.500	0.000	1.000
Cr	17.864	16.833	−0.289	0.773
Cs	18.455	15.750	−0.757	0.449
Cu	16.864	18.667	0.505	0.614
Dy	16.591	19.167	0.730	0.465

Er	15.636	20.917	1.497	0.135
Eu	18.341	15.958	−0.668	0.504
Fe	17.636	17.250	−0.108	0.914
Ga	17.682	17.167	−0.154	0.878
Gd	19.091	14.583	−1.261	0.207
Hf	17.295	17.875	0.398	0.691
Hg	17.773	17.000	−0.216	0.829
Hg2	18.045	16.500	−0.432	0.665
Ho	18.091	16.417	−0.516	0.606
K	18.136	16.333	−0.505	0.614
La	20.000	12.917	−1.983	0.047
Mg	16.409	19.500	0.865	0.387
Mn	18.591	15.500	−0.865	0.387
Mo	18.909	14.917	−1.118	0.264
Na	17.227	18.000	0.216	0.829
Nd	19.273	14.250	−1.406	0.160
Ni	17.500	17.500	0.000	1.000
P	16.000	20.250	1.189	0.234
Pb	20.455	12.083	−2.353	0.019
Pd	20.023	12.875	−2.113	0.035
Pr	20.364	12.250	−2.271	0.023
Pt	20.182	12.583	−2.126	0.033

Rb	18.545	15.583	−0.829	0.407
Sb	18.591	15.500	−1.545	0.122
Se	16.273	19.750	0.973	0.331
Se2	16.455	19.417	0.829	0.407
Sm	16.886	18.625	0.490	0.624
Sn	16.591	19.167	0.721	0.471
Sr	19.318	14.167	−1.441	0.149
Tb	16.818	18.750	0.548	0.584
Ti	17.500	17.500	0.000	1.000
Tl	17.773	17.000	−0.739	0.460
Tm	18.068	16.458	−0.453	0.650
U	19.727	13.417	−1.978	0.048
V	18.818	15.083	−1.045	0.296
Yb	17.455	17.583	0.037	0.971
Zn	18.182	16.250	−0.541	0.589
Zr	18.455	15.750	−0.767	0.443

4.cingulate gyrus (D)

Group	variable	n	min	mean	median	max	sd
control	Ag	19	0.000	5.388	5.498	15.036	4.340
suicide	Ag	13	0.000	9.042	7.405	23.363	8.557
control	Al	19	2.876	324.694	165.970	1411.349	418.047

suicide	Al	13	54.023	186.629	121.447	630.113	164.598
control	As	19	0.000	0.000	0.000	0.000	0.000
suicide	As	13	0.000	0.000	0.000	0.000	0.000
control	As2	19	0.000	0.000	0.000	0.000	0.000
suicide	As2	13	0.000	0.000	0.000	0.000	0.000
control	Ba	19	0.816	10.026	6.273	41.281	10.425
suicide	Ba	13	0.992	5.362	5.088	11.602	3.610

control	Be	19	0.000	0.010	0.000	0.139	0.033
suicide	Be	13	0.000	0.107	0.000	1.228	0.339
control	Bi	19	0.000	3.888	0.592	60.037	13.615
suicide	Bi	13	0.000	12.448	0.557	91.248	29.189
control	Ca	19	60.467	90.414	82.857	194.812	31.099
suicide	Ca	13	48.454	76.443	73.646	157.062	28.143
control	Cd	19	5.649	20.145	16.081	58.322	13.596
suicide	Cd	13	3.102	24.099	15.033	85.790	24.018
control	Ce	19	0.000	0.199	0.150	0.831	0.210
suicide	Ce	13	0.000	0.217	0.093	1.367	0.358
control	Co	19	0.000	1.639	1.956	3.650	1.208
suicide	Co	13	0.000	1.364	1.617	3.633	1.401
control	Cr	19	0.000	38.171	11.670	335.231	75.621
suicide	Cr	13	0.000	24.862	8.936	193.769	52.026
control	Cs	19	1.073	5.155	4.910	11.027	2.551
suicide	Cs	13	2.683	5.641	4.814	11.186	2.628
control	Cu	19	1881.420	3788.522	3656.539	5364.388	1022.133
suicide	Cu	13	2344.261	4456.747	4647.757	5839.116	1006.094
control	Dy	19	0.000	0.012	0.013	0.057	0.015
suicide	Dy	13	0.000	0.016	0.010	0.054	0.016
control	Er	19	0.000	0.032	0.011	0.149	0.046
suicide	Er	13	0.000	0.025	0.000	0.134	0.044
control	Eu	19	0.000	0.008	0.005	0.022	0.007
suicide	Eu	13	0.000	0.010	0.008	0.040	0.011
control	Fe	19	19455.222	39319.885	35959.813	77233.143	15689.844
suicide	Fe	13	26156.986	53066.501	48569.808	112775.758	25589.518

Group	variable	n	min	mean	median	max	sd
control	Ga	19	0.000	0.132	0.011	0.548	0.192
suicide	Ga	13	0.000	0.323	0.000	3.416	0.937
control	Gd	19	0.004	0.037	0.034	0.130	0.030
suicide	Gd	13	0.000	0.100	0.023	0.941	0.254
control	Hf	19	0.000	0.011	0.000	0.176	0.041
suicide	Hf	13	0.000	0.051	0.000	0.661	0.183
control	Hg	19	1.183	3.630	2.840	8.726	2.173
suicide	Hg	13	0.949	3.299	3.044	5.756	1.806
control	Hg2	19	1.054	3.622	2.710	8.814	2.167
suicide	Hg2	13	0.878	3.284	3.157	5.640	1.747
control	Ho	19	0.000	0.003	0.000	0.033	0.008
suicide	Ho	13	0.000	0.007	0.000	0.057	0.016
control	K	19	1907.172	2698.900	2788.362	3702.990	520.555
suicide	K	13	2166.160	2897.808	2909.221	4012.573	536.931
control	La	19	0.000	0.104	0.082	0.411	0.104
suicide	La	13	0.000	0.098	0.085	0.236	0.072
control	Mg	19	73.404	102.948	102.616	134.563	21.599
suicide	Mg	13	64.816	106.238	101.423	144.389	26.260
control	Mn	19	147.216	214.275	214.457	252.249	29.420
suicide	Mn	13	170.130	227.770	221.207	337.337	45.367
control	Mo	19	0.000	124.057	110.833	257.123	67.466
suicide	Mo	13	0.000	118.204	103.838	277.139	82.432
control	Na	19	1263.757	2020.034	1912.555	2750.785	477.204
suicide	Na	13	1328.989	1983.693	2048.590	2449.808	307.814
control	Nd	19	0.000	0.079	0.049	0.360	0.087
suicide	Nd	13	0.000	0.109	0.042	0.908	0.242

Group	variable	n	min	mean	median	max	sd
control	Ni	19	0.000	5.843	6.232	22.113	5.400
suicide	Ni	13	0.000	4.696	3.223	12.732	4.348
control	P	19	1283.508	2048.463	1998.311	2989.107	396.737
suicide	P	13	1829.026	2263.668	2098.591	2955.806	391.844
control	Pb	19	0.000	2.338	2.378	7.290	2.069
suicide	Pb	13	0.000	4.233	1.489	38.905	10.493
control	Pd	19	0.000	0.120	0.084	0.632	0.157
suicide	Pd	13	0.000	0.060	0.024	0.199	0.073
control	Pr	19	0.000	0.020	0.016	0.063	0.018
suicide	Pr	13	0.000	0.030	0.009	0.252	0.068
control	Pt	19	0.000	0.068	0.032	0.737	0.164
suicide	Pt	13	0.000	0.648	0.017	7.644	2.106
control	Rb	19	665.242	2115.658	2078.465	3046.300	766.218
suicide	Rb	13	1750.753	2231.462	2046.406	3191.751	492.306
control	Sb	19	0.000	0.000	0.000	0.000	0.000
suicide	Sb	13	0.000	0.000	0.000	0.000	0.000
control	Se	19	91.309	138.991	137.842	187.393	29.690
suicide	Se	13	90.059	145.539	151.381	185.706	31.595
control	Se2	19	78.283	124.955	127.286	166.102	26.182
suicide	Se2	13	85.408	128.811	135.003	158.530	22.740
control	Sm	19	0.000	0.022	0.013	0.125	0.032
suicide	Sm	13	0.000	0.020	0.015	0.055	0.018
control	Sn	19	0.000	2.085	1.258	8.454	2.133
suicide	Sn	13	0.000	1.012	0.840	4.376	1.237
control	Sr	19	63.181	331.001	251.563	1095.960	266.262
suicide	Sr	13	65.063	171.568	145.258	382.941	91.778

Group	variable	n	min	mean	median	max	sd
control	Tb	19	0.000	0.005	0.005	0.013	0.004
suicide	Tb	13	0.000	0.004	0.002	0.014	0.005
control	Th	19	0.000	0.000	0.000	0.000	0.000
suicide	Th	13	0.000	0.000	0.000	0.000	0.000
control	Ti	19	10.284	25.826	25.190	53.455	10.225
suicide	Ti	13	14.135	24.855	25.379	36.157	6.406
control	Tl	19	0.000	0.000	0.000	0.000	0.000
suicide	Tl	13	0.000	0.000	0.000	0.000	0.000
control	Tm	19	0.000	0.009	0.006	0.026	0.008
suicide	Tm	13	0.000	0.009	0.009	0.025	0.008
control	U	19	0.000	0.068	0.016	0.379	0.112
suicide	U	13	0.000	0.013	0.000	0.099	0.033
control	V	19	1.033	3.696	2.184	22.357	4.755
suicide	V	13	0.283	3.202	1.904	15.039	3.812
control	Yb	19	0.000	0.018	0.003	0.083	0.027
suicide	Yb	13	0.000	0.014	0.013	0.044	0.016
control	Zn	19	9925.616	17013.975	14193.708	30709.642	6259.832
suicide	Zn	13	13956.860	19934.963	17829.641	29564.421	6023.234
control	Zr	19	0.000	0.960	0.769	3.063	0.861
suicide	Zr	13	0.000	3.333	0.220	39.492	10.873

IV.cingulate gyrus (D)

Element	Control mean	Suicide mean	Dunn statistic	p
Ag	15.368	18.154	0.826	0.409

Al	17.316	15.308	−0.595	0.552
Ba	18.000	14.308	−1.094	0.274
Be	15.684	17.692	0.941	0.347
Bi	16.368	16.692	0.096	0.924
Ca	18.895	13.000	−1.746	0.081
Cd	16.842	16.000	−0.249	0.803
Ce	17.026	15.731	−0.384	0.701
Co	17.105	15.615	−0.450	0.652
Cr	17.526	15.000	−0.751	0.453
Cs	16.105	17.077	0.288	0.774
Cu	14.105	20.000	1.746	0.081
Dy	15.026	18.654	1.083	0.279
Er	17.474	15.077	−0.749	0.454
Eu	16.263	16.846	0.174	0.862
Fe	14.737	19.077	1.285	0.199
Ga	17.789	14.615	−1.019	0.308
Gd	17.263	15.385	−0.556	0.578
Hf	16.184	16.962	0.401	0.689
Hg	16.895	15.923	−0.288	0.774
Hg2	16.737	16.154	−0.173	0.863
Ho	14.684	19.154	1.669	0.095
K	15.211	18.385	0.940	0.347

La	16.132	17.038	0.269	0.788
Mg	15.789	17.538	0.518	0.604
Mn	15.632	17.769	0.633	0.527
Mo	16.711	16.192	−0.153	0.878
Na	16.474	16.538	0.019	0.985
Nd	17.474	15.077	−0.710	0.477
Ni	16.947	15.846	−0.326	0.744
P	14.632	19.231	1.362	0.173
Pb	17.632	14.846	−0.834	0.404
Pd	17.737	14.692	−0.920	0.357
Pr	17.842	14.538	−0.983	0.325
Pt	17.158	15.538	−0.480	0.631
Rb	16.053	17.154	0.326	0.744
Se	15.789	17.538	0.518	0.604
Se2	16.000	17.231	0.365	0.715
Sm	15.816	17.500	0.500	0.617
Sn	19.079	12.731	−1.882	0.060
Sr	19.053	12.769	−1.861	0.063
Tb	18.053	14.231	−1.134	0.257
Ti	16.579	16.385	−0.058	0.954
Tm	16.842	16.000	−0.250	0.803
U	18.921	12.962	−2.030	0.042

V	17.421	15.154	−0.671	0.502
Yb	16.395	16.654	0.079	0.937
Zn	14.263	19.769	1.631	0.103
Zr	19.158	12.615	−1.944	0.052

5.hippocampus (E)

Group	variable	n	min	mean	median	max	sd
control	Ag	21	0.000	2.267	1.690	6.050	2.268
suicide	Ag	12	0.000	5.504	4.885	16.366	4.932
control	Al	21	0.000	329.715	209.639	1541.081	405.394
suicide	Al	12	26.081	113.559	113.979	226.389	57.205
control	As	21	0.000	0.000	0.000	0.000	0.000
suicide	As	12	0.000	0.000	0.000	0.000	0.000
control	As2	21	0.000	0.000	0.000	0.000	0.000
suicide	As2	12	0.000	0.000	0.000	0.000	0.000
control	Ba	21	1.858	6.872	5.005	28.935	5.799
suicide	Ba	12	0.636	5.190	4.943	9.359	2.598
control	Be	21	0.000	0.009	0.000	0.137	0.031
suicide	Be	12	0.000	0.000	0.000	0.000	0.000
control	Bi	21	0.000	13.934	0.425	272.246	59.229
suicide	Bi	12	0.000	6.502	0.310	73.211	21.012
control	Ca	21	57.944	105.867	79.675	272.835	63.566
suicide	Ca	12	52.906	95.869	62.487	205.221	56.487
control	Cd	21	5.660	32.318	24.613	90.672	25.044

suicide	Cd	12	4.965	18.169	14.709	39.195	12.883
control	Ce	21	0.017	0.693	0.183	2.851	0.949
suicide	Ce	12	0.024	0.252	0.146	1.003	0.324
control	Co	21	0.000	2.699	1.869	16.961	3.624
suicide	Co	12	0.000	1.477	1.844	3.263	1.198
control	Cr	21	0.000	20.251	6.699	99.163	26.687
suicide	Cr	12	0.000	7.646	4.129	41.934	11.648
control	Cs	21	1.271	6.054	5.266	14.559	3.518
suicide	Cs	12	3.588	6.441	5.334	14.005	3.151
control	Cu	21	1266.407	3174.812	3028.772	5253.612	961.245
suicide	Cu	12	2199.972	3409.158	3550.570	4272.795	644.398
control	Dy	21	0.000	0.014	0.008	0.062	0.020
suicide	Dy	12	0.000	0.010	0.007	0.037	0.011
control	Er	21	0.000	0.035	0.004	0.211	0.053
suicide	Er	12	0.000	0.054	0.045	0.158	0.056
control	Eu	21	0.000	0.008	0.007	0.017	0.006
suicide	Eu	12	0.000	0.008	0.005	0.025	0.009
control	Fe	21	23394.322	45093.377	43363.129	86175.898	16034.951
suicide	Fe	12	24294.481	47952.008	41863.361	72246.944	17202.135
control	Ga	21	0.000	0.037	0.000	0.277	0.077
suicide	Ga	12	0.000	0.099	0.002	0.511	0.176
control	Gd	21	0.000	0.079	0.033	0.627	0.137
suicide	Gd	12	0.001	0.039	0.033	0.098	0.032
control	Hf	21	0.000	0.000	0.000	0.000	0.000
suicide	Hf	12	0.000	0.030	0.000	0.356	0.103
control	Hg	21	0.989	4.201	2.839	17.204	3.935
suicide	Hg	12	0.492	3.088	3.363	5.743	1.672

control	Hg2	21	0.898	4.165	2.490	17.391	3.956
suicide	Hg2	12	0.479	3.091	3.290	5.797	1.643
control	Ho	21	0.000	0.003	0.000	0.044	0.010
suicide	Ho	12	0.000	0.007	0.000	0.036	0.013
control	K	21	1790.197	2638.254	2376.671	4913.281	812.106
suicide	K	12	1834.462	2582.068	2590.945	3228.724	444.159
control	La	21	0.000	0.484	0.124	2.150	0.723
suicide	La	12	0.010	0.170	0.077	0.760	0.242
control	Mg	21	64.708	109.070	94.332	232.542	42.982
suicide	Mg	12	65.140	97.615	88.147	139.805	24.987
control	Mn	21	177.083	298.094	277.556	699.543	105.322
suicide	Mn	12	197.577	274.084	257.945	399.199	72.171
control	Mo	21	0.000	143.771	103.003	482.133	128.773
suicide	Mo	12	0.000	109.242	119.805	234.660	72.844
control	Na	21	1216.773	1892.168	1764.005	3034.496	556.994
suicide	Na	12	1420.179	1815.118	1743.235	2274.930	254.497
control	Nd	21	0.008	0.101	0.081	0.582	0.125
suicide	Nd	12	0.000	0.055	0.043	0.181	0.053
control	Ni	21	0.000	6.411	4.076	37.061	9.109
suicide	Ni	12	0.000	3.938	2.612	9.624	3.692
control	P	21	1519.833	2246.508	2000.013	4221.633	671.572
suicide	P	12	1854.685	2269.550	2300.568	2690.883	241.211
control	Pb	21	0.000	4.729	2.362	33.589	7.304
suicide	Pb	12	0.000	4.213	1.624	22.605	6.473
control	Pd	21	0.000	0.056	0.000	0.274	0.084
suicide	Pd	12	0.000	0.079	0.042	0.374	0.106
control	Pr	21	0.000	0.039	0.024	0.186	0.043

suicide	Pr	12	0.000	0.024	0.018	0.108	0.032
control	Pt	21	0.006	3.981	0.044	74.776	16.292
suicide	Pt	12	0.000	0.821	0.022	9.584	2.760
control	Rb	21	701.021	2322.126	2013.645	4873.306	1072.321
suicide	Rb	12	1585.102	2234.269	2039.671	3293.390	513.973
control	Sb	21	0.000	0.039	0.000	0.792	0.173
suicide	Sb	12	0.000	0.000	0.000	0.004	0.001
control	Se	21	73.357	140.155	131.749	219.614	35.954
suicide	Se	12	100.809	144.152	141.572	177.571	25.281
control	Se2	21	75.741	127.996	121.025	199.751	33.676
suicide	Se2	12	77.299	126.731	126.310	169.537	27.141
control	Sm	21	0.000	0.028	0.012	0.111	0.038
suicide	Sm	12	0.000	0.013	0.008	0.036	0.013
control	Sn	21	0.000	2.272	1.702	15.435	3.253
suicide	Sn	12	0.000	1.527	1.291	5.252	1.423
control	Sr	21	61.111	180.963	176.048	487.149	88.133
suicide	Sr	12	65.273	183.723	117.663	519.012	144.656
control	Tb	21	0.000	0.009	0.006	0.042	0.010
suicide	Tb	12	0.000	0.006	0.004	0.016	0.005
control	Th	21	0.000	0.000	0.000	0.000	0.000
suicide	Th	12	0.000	0.000	0.000	0.000	0.000
control	Ti	21	12.509	29.165	27.294	64.912	13.240
suicide	Ti	12	12.455	23.454	26.025	31.598	6.405
control	Tl	21	0.000	0.001	0.000	0.028	0.006
suicide	Tl	12	0.000	0.000	0.000	0.000	0.000
control	Tm	21	0.000	0.009	0.005	0.043	0.011
suicide	Tm	12	0.000	0.011	0.009	0.028	0.010

control	U	21	0.000	0.021	0.000	0.096	0.032
suicide	U	12	0.000	0.009	0.000	0.061	0.020
control	V	21	0.674	3.827	2.980	9.780	2.791
suicide	V	12	0.511	1.964	1.972	3.803	0.969
control	Yb	21	0.000	0.025	0.023	0.105	0.029
suicide	Yb	12	0.000	0.020	0.012	0.053	0.021
control	Zn	21	13428.721	21111.080	20052.214	34072.798	6082.039
suicide	Zn	12	15008.576	21569.099	18477.030	34579.132	6990.045
control	Zr	21	0.000	0.490	0.292	2.528	0.581
suicide	Zr	12	0.000	0.627	0.395	2.405	0.800

V. hippocampus (E)

Element	Control mean	Suicide mean	Dunn statistic	p
Ag	14.571	21.250	1.918	0.055
Al	19.190	13.167	−1.722	0.085
Ba	17.286	16.500	−0.225	0.822
Be	17.571	16.000	−1.086	0.278
Bi	17.714	15.750	−0.562	0.574
Ca	18.619	14.167	−1.272	0.203
Cd	19.333	12.917	−1.834	0.067
Ce	18.667	14.083	−1.310	0.190
Co	18.048	15.167	−0.832	0.406
Cr	17.857	15.500	−0.677	0.499
Cs	16.619	17.667	0.299	0.765
Cu	15.952	18.833	0.823	0.410
Dy	17.095	16.833	−0.076	0.940
Er	15.571	19.500	1.159	0.247
Eu	17.429	16.250	−0.338	0.735
Fe	16.190	18.417	0.636	0.525
Ga	16.048	18.667	0.832	0.405
Gd	17.476	16.167	−0.374	0.708
Hf	16.500	17.875	1.323	0.186
Hg	17.143	16.750	−0.112	0.911

Hg2	17.190	16.667	−0.150	0.881
Ho	16.333	18.167	0.668	0.504
K	16.333	18.167	0.524	0.600
La	18.286	14.750	−1.010	0.312
Mg	17.667	15.833	−0.524	0.600
Mn	17.905	15.417	−0.711	0.477
Mo	17.476	16.167	−0.375	0.708
Na	17.000	17.000	0.000	1.000
Nd	18.667	14.083	−1.310	0.190
Ni	17.333	16.417	−0.262	0.793
P	15.238	20.083	1.385	0.166
Pb	17.952	15.333	−0.751	0.453
Pd	16.048	18.667	0.786	0.432
Pr	18.833	13.792	−1.442	0.149
Pt	18.667	14.083	−1.310	0.190
Rb	16.571	17.750	0.337	0.736
Sb	17.119	16.792	−0.188	0.851
Se	16.524	17.833	0.374	0.708
Se2	16.810	17.333	0.150	0.881
Sm	17.524	16.083	−0.416	0.678
Sn	17.524	16.083	−0.412	0.680
Sr	18.429	14.500	−1.123	0.262

Tb	17.571	16.000	−0.450	0.652
Ti	18.190	14.917	−0.936	0.349
Tl	17.286	16.500	−0.756	0.450
Tm	16.143	18.500	0.676	0.499
U	18.452	14.458	−1.294	0.196
V	19.238	13.083	−1.759	0.079
Yb	17.095	16.833	−0.076	0.940
Zn	16.905	17.167	0.075	0.940
Zr	17.024	16.958	−0.019	0.985

6.head of caudate nucleus (F)

Group	variable	n	min	mean	median	max	sd
control	Ag	22	0.000	5.515	2.824	46.203	9.713
suicide	Ag	13	0.360	8.402	6.897	27.213	8.422
control	Al	22	29.855	240.870	166.097	1103.017	250.037
suicide	Al	13	4.226	184.117	113.689	522.944	168.072
control	As	22	0.000	0.000	0.000	0.000	0.000
suicide	As	13	0.000	0.000	0.000	0.000	0.000
control	As2	22	0.000	0.000	0.000	0.000	0.000
suicide	As2	13	0.000	0.000	0.000	0.000	0.000
control	Ba	22	0.898	5.034	4.944	11.982	2.419
suicide	Ba	13	1.363	5.035	4.954	11.002	2.898
control	Be	22	0.000	0.121	0.000	2.450	0.521
suicide	Be	13	0.000	0.002	0.000	0.029	0.008
control	Bi	22	0.000	15.798	1.400	314.510	66.731

suicide	Bi	13	0.313	7.345	1.614	76.644	20.863
control	Ca	22	24.469	75.388	67.912	135.730	25.344
suicide	Ca	13	50.466	61.331	58.894	77.268	8.229
control	Cd	22	5.702	28.636	21.198	80.636	20.835
suicide	Cd	13	6.460	32.274	27.946	72.259	24.045
control	Ce	22	0.028	0.258	0.176	1.294	0.273
suicide	Ce	13	0.029	0.291	0.228	0.879	0.224
control	Co	22	0.000	1.797	2.147	4.859	1.507
suicide	Co	13	0.000	1.868	2.069	4.108	1.690
control	Cr	22	0.000	15.351	4.192	141.563	31.049
suicide	Cr	13	3.035	11.400	9.131	33.877	8.966
control	Cs	22	1.445	5.843	6.432	13.069	2.759
suicide	Cs	13	3.009	6.530	6.149	11.973	2.437
control	Cu	22	1384.025	4653.551	4426.766	9917.516	1765.089
suicide	Cu	13	3514.389	5054.742	5001.546	6797.928	1057.434
control	Dy	22	0.000	0.011	0.007	0.030	0.009
suicide	Dy	13	0.000	0.011	0.010	0.030	0.010
control	Er	22	0.000	0.033	0.014	0.228	0.053
suicide	Er	13	0.000	0.025	0.006	0.078	0.030
control	Eu	22	0.000	0.007	0.004	0.032	0.009
suicide	Eu	13	0.000	0.007	0.006	0.019	0.006
control	Fe	22	25951.014	116974.489	99679.482	278763.116	55058.240
suicide	Fe	13	94706.231	137544.121	124728.428	260418.527	43793.365
control	Ga	22	0.000	0.458	0.002	8.539	1.808
suicide	Ga	13	0.000	0.064	0.000	0.477	0.132
control	Gd	22	0.000	0.130	0.026	2.231	0.470
suicide	Gd	13	0.016	0.059	0.030	0.247	0.076

control	Hf	22	0.000	0.008	0.000	0.093	0.023
suicide	Hf	13	0.000	0.011	0.000	0.061	0.021
control	Hg	22	0.703	3.707	2.660	10.286	2.964
suicide	Hg	13	0.841	3.943	3.567	10.248	2.570
control	Hg2	22	0.673	3.694	2.627	10.055	2.920
suicide	Hg2	13	0.684	3.871	3.506	9.777	2.489
control	Ho	22	0.000	0.007	0.000	0.062	0.015
suicide	Ho	13	0.000	0.004	0.000	0.040	0.012
control	K	22	905.593	2581.050	2492.737	4423.881	744.527
suicide	K	13	2026.257	2550.517	2583.055	2850.529	244.852
control	La	22	0.000	0.158	0.110	0.804	0.173
suicide	La	13	0.024	0.180	0.141	0.495	0.137
control	Mg	22	41.069	107.579	109.436	183.548	33.029
suicide	Mg	13	73.640	104.964	106.238	138.710	17.365
control	Mn	22	81.753	387.275	413.136	580.747	101.759
suicide	Mn	13	277.342	411.277	398.029	706.006	102.712
control	Mo	22	0.000	418.496	411.255	764.265	203.897
suicide	Mo	13	239.365	549.196	546.339	920.014	214.710
control	Na	22	767.699	1784.643	1838.139	2369.331	409.244
suicide	Na	13	1379.439	1752.317	1700.858	2135.849	246.008
control	Nd	22	0.000	0.069	0.054	0.165	0.043
suicide	Nd	13	0.018	0.077	0.067	0.271	0.066
control	Ni	22	0.000	3.410	1.864	11.365	3.888
suicide	Ni	13	0.000	2.886	2.134	9.001	2.669
control	P	22	827.132	2363.131	2220.705	4352.181	814.272
suicide	P	13	1841.028	2387.601	2476.247	2987.775	369.160
control	Pb	22	0.000	6.028	3.449	52.024	10.690

suicide	Pb	13	0.000	2.564	2.519	5.509	1.781
control	Pd	22	0.000	0.092	0.032	0.464	0.128
suicide	Pd	13	0.000	0.080	0.093	0.208	0.076
control	Pr	22	0.000	0.019	0.017	0.047	0.014
suicide	Pr	13	0.000	0.027	0.023	0.086	0.020
control	Pt	22	0.000	0.031	0.026	0.173	0.036
suicide	Pt	13	0.000	0.022	0.017	0.065	0.019
control	Rb	22	729.152	2338.175	2443.791	4337.415	927.656
suicide	Rb	13	1572.515	2385.298	2397.145	3304.543	446.716
control	Sb	22	0.000	0.029	0.000	0.310	0.071
suicide	Sb	13	0.000	0.016	0.000	0.167	0.047
control	Se	22	42.303	168.024	171.048	260.563	44.994
suicide	Se	13	102.667	175.595	178.634	221.126	32.864
control	Se2	22	32.901	150.741	153.768	236.317	41.401
suicide	Se2	13	94.126	156.381	161.941	197.150	31.490
control	Sm	22	0.000	0.019	0.012	0.122	0.026
suicide	Sm	13	0.000	0.012	0.006	0.040	0.013
control	Sn	22	0.000	1.826	1.022	6.315	1.876
suicide	Sn	13	0.000	2.046	1.405	5.831	1.811
control	Sr	22	42.016	175.816	142.372	609.245	135.248
suicide	Sr	13	36.334	221.790	101.713	861.478	272.264
control	Tb	22	0.000	0.003	0.003	0.008	0.003
suicide	Tb	13	0.000	0.003	0.003	0.009	0.003
control	Th	22	0.000	0.000	0.000	0.000	0.000
suicide	Th	13	0.000	0.001	0.000	0.012	0.003
control	Ti	22	10.114	22.714	19.130	50.976	11.049
suicide	Ti	13	13.554	23.892	23.296	34.054	7.446

control	Tl	22	0.000	0.006	0.000	0.095	0.022
suicide	Tl	13	0.000	0.000	0.000	0.000	0.000
control	Tm	22	0.000	0.007	0.003	0.030	0.008
suicide	Tm	13	0.000	0.005	0.004	0.019	0.006
control	U	22	0.000	0.027	0.000	0.264	0.060
suicide	U	13	0.000	0.010	0.000	0.072	0.021
control	V	22	0.444	2.070	1.895	6.864	1.493
suicide	V	13	0.477	1.342	1.006	2.841	0.771
control	Yb	22	0.000	0.022	0.021	0.115	0.025
suicide	Yb	13	0.000	0.015	0.011	0.045	0.015
control	Zn	22	8599.896	16216.079	13567.778	40672.160	7159.289
suicide	Zn	13	12253.903	16850.829	14916.825	25971.074	4775.584
control	Zr	22	0.000	0.802	0.265	5.087	1.219
suicide	Zr	13	0.000	0.955	0.358	3.846	1.330

VI.head of caudate nucleus (F)

Element	Control mean	Suicide mean	Dunn statistic	p
Ag	15.955	21.462	1.536	0.124
Al	18.545	17.077	−0.410	0.682
Ba	18.364	17.385	−0.273	0.785
Be	18.455	17.231	−0.618	0.537
Bi	18.045	17.923	−0.034	0.973
Ca	20.727	13.385	−2.048	0.041
Cd	17.818	18.308	0.137	0.891
Ce	16.818	20.000	0.888	0.375
Co	17.841	18.269	0.122	0.903
Cr	16.136	21.154	1.401	0.161
Cs	17.636	18.615	0.273	0.785
Cu	16.273	20.923	1.297	0.195
Dy	17.955	18.077	0.034	0.973
Er	17.864	18.231	0.107	0.915
Eu	17.318	19.154	0.515	0.606
Fe	15.136	22.846	2.151	0.031
Ga	18.795	16.654	−0.643	0.520
Gd	17.091	19.538	0.683	0.495
Hf	17.636	18.615	0.391	0.696
Hg	17.273	19.231	0.546	0.585

Hg2	17.409	19.000	0.444	0.657
Ho	18.932	16.423	−0.911	0.362
K	17.591	18.692	0.307	0.759
La	17.136	19.462	0.649	0.517
Mg	18.455	17.231	−0.341	0.733
Mn	18.273	17.538	−0.205	0.838
Mo	15.955	21.462	1.536	0.124
Na	18.545	17.077	−0.410	0.682
Nd	18.273	17.538	−0.205	0.838
Ni	18.000	18.000	0.000	1.000
P	17.273	19.231	0.546	0.585
Pb	19.591	15.308	−1.197	0.231
Pd	17.682	18.538	0.243	0.808
Pr	16.682	20.231	0.990	0.322
Pt	19.318	15.769	−0.990	0.322
Rb	18.136	17.769	−0.102	0.918
Sb	18.523	17.115	−0.562	0.574
Se	17.364	19.077	0.478	0.633
Se2	17.500	18.846	0.376	0.707
Sm	19.159	16.038	−0.873	0.383
Sn	17.227	19.308	0.581	0.561
Sr	18.636	16.923	−0.478	0.633

Tb	18.000	18.000	0.000	1.000
Th	17.500	18.846	1.301	0.193
Ti	16.682	20.231	0.990	0.322
Tl	18.591	17.000	−1.103	0.270
Tm	18.727	16.769	−0.565	0.572
U	18.727	16.769	−0.606	0.545
V	20.045	14.538	−1.536	0.124
Yb	19.136	16.077	−0.861	0.389
Zn	16.682	20.231	0.990	0.322
Zr	17.500	18.846	0.377	0.706

7. superior longitudinal fasciculus of brain (G)

Group	variable	n	min	mean	median	max	sd
control	Ag	23	0.000	2.739	2.974	7.835	2.531
suicide	Ag	13	0.039	8.271	6.245	30.531	9.152
control	Al	23	0.000	226.041	164.125	978.983	225.457
suicide	Al	13	36.894	139.143	118.466	514.937	121.318
control	As	23	0.000	0.000	0.000	0.000	0.000
suicide	As	13	0.000	0.419	0.000	3.751	1.077
control	As2	23	0.000	0.000	0.000	0.000	0.000
suicide	As2	13	0.000	0.268	0.000	2.412	0.709
control	Ba	23	1.060	5.647	4.237	11.390	3.284
suicide	Ba	13	0.441	4.119	2.960	10.291	3.180

Group	variable	n	min	mean	median	max	sd
control	Be	23	0.000	0.002	0.000	0.037	0.008
suicide	Be	13	0.000	0.014	0.000	0.118	0.036
control	Bi	23	0.000	1.455	0.428	14.993	3.174
suicide	Bi	13	0.000	12.202	0.438	115.231	32.536
control	Ca	23	46.412	68.263	65.789	136.028	19.329
suicide	Ca	13	45.525	62.288	59.319	80.170	11.372
control	Cd	23	3.824	30.200	25.024	96.625	24.468
suicide	Cd	13	3.909	20.548	15.230	60.090	18.810
control	Ce	23	0.025	0.348	0.195	1.922	0.520
suicide	Ce	13	0.000	0.191	0.177	0.667	0.194
control	Co	23	0.000	1.325	1.484	4.950	1.377
suicide	Co	13	0.000	1.788	1.565	8.263	2.330
control	Cr	23	0.000	25.122	5.235	185.455	44.844
suicide	Cr	13	0.000	11.274	6.921	60.546	16.227
control	Cs	23	2.042	6.856	7.327	13.710	2.655
suicide	Cs	13	4.466	7.357	6.797	13.806	2.598
control	Cu	23	2195.864	3835.274	3507.614	10842.750	1703.350
suicide	Cu	13	2325.222	3953.407	3947.848	7143.547	1254.079
control	Dy	23	0.000	0.012	0.009	0.057	0.013
suicide	Dy	13	0.000	0.009	0.008	0.032	0.011
control	Er	23	0.000	0.036	0.022	0.126	0.039
suicide	Er	13	0.000	0.016	0.006	0.059	0.020
control	Eu	23	0.000	0.007	0.004	0.051	0.011
suicide	Eu	13	0.000	0.008	0.007	0.017	0.006
control	Fe	23	30701.006	65850.883	55155.614	176536.741	31800.922
suicide	Fe	13	28214.422	66474.773	55120.138	127172.250	30497.580

Group	variable	n	min	mean	median	max	sd
control	Ga	23	0.000	0.036	0.000	0.223	0.062
suicide	Ga	13	0.000	0.229	0.005	2.140	0.597
control	Gd	23	0.001	0.060	0.035	0.518	0.104
suicide	Gd	13	0.000	0.041	0.021	0.128	0.044
control	Hf	23	0.000	0.001	0.000	0.025	0.005
suicide	Hf	13	0.000	0.002	0.000	0.021	0.006
control	Hg	23	0.595	3.292	2.750	8.407	2.295
suicide	Hg	13	0.244	2.720	2.541	7.519	1.963
control	Hg2	23	0.674	3.281	2.797	8.420	2.285
suicide	Hg2	13	0.248	2.710	2.601	7.304	1.911
control	Ho	23	0.000	0.009	0.000	0.054	0.015
suicide	Ho	13	0.000	0.007	0.000	0.079	0.022
control	K	23	2164.939	2988.938	2721.257	7689.984	1089.224
suicide	K	13	2157.321	2721.563	2657.551	3969.922	454.776
control	La	23	0.000	0.226	0.092	1.622	0.420
suicide	La	13	0.035	0.135	0.103	0.430	0.121
control	Mg	23	91.670	145.644	146.207	316.028	44.782
suicide	Mg	13	90.222	126.709	125.772	173.849	26.453
control	Mn	23	241.395	359.512	353.343	553.481	82.906
suicide	Mn	13	204.861	369.223	346.703	722.683	148.717
control	Mo	23	0.000	61.915	50.339	355.377	85.062
suicide	Mo	13	0.000	45.368	29.729	168.963	52.685
control	Na	23	1001.411	1658.543	1613.204	4573.973	687.631
suicide	Na	13	1091.304	1519.906	1526.796	1947.508	224.281
control	Nd	23	0.006	0.076	0.056	0.267	0.062
suicide	Nd	13	0.011	0.058	0.050	0.167	0.045

Group	variable	n	min	mean	median	max	sd
control	Ni	23	0.000	5.269	3.247	19.866	4.759
suicide	Ni	13	0.000	2.056	1.059	7.974	2.419
control	P	23	2918.775	4393.309	4191.446	6707.008	826.470
suicide	P	13	2841.544	4216.507	4274.560	5489.908	746.901
control	Pb	23	0.000	2.139	1.741	7.146	2.153
suicide	Pb	13	0.000	1.205	1.227	2.769	1.009
control	Pd	23	0.000	0.044	0.014	0.234	0.063
suicide	Pd	13	0.000	0.049	0.000	0.184	0.068
control	Pr	23	0.000	0.023	0.021	0.095	0.022
suicide	Pr	13	0.000	0.016	0.010	0.058	0.017
control	Pt	23	0.000	0.054	0.021	0.459	0.098
suicide	Pt	13	0.001	0.086	0.024	0.832	0.225
control	Rb	23	869.195	2412.281	2416.914	4369.126	745.013
suicide	Rb	13	1995.176	2409.641	2275.481	3255.814	402.129
control	Sb	23	0.000	0.016	0.000	0.366	0.076
suicide	Sb	13	0.000	0.003	0.000	0.042	0.012
control	Se	23	85.590	152.040	134.512	408.057	62.146
suicide	Se	13	107.481	146.346	150.871	188.740	24.584
control	Se2	23	75.683	135.869	121.737	325.844	51.391
suicide	Se2	13	96.082	130.331	136.012	175.495	24.188
control	Sm	23	0.000	0.017	0.018	0.049	0.013
suicide	Sm	13	0.000	0.007	0.005	0.032	0.009
control	Sn	23	0.000	1.571	1.139	8.129	1.764
suicide	Sn	13	0.000	2.284	1.277	9.330	2.869
control	Sr	23	38.544	191.694	146.839	730.160	174.533
suicide	Sr	13	37.852	129.292	113.515	242.077	60.009

Group	variable	n	min	mean	median	max	sd
control	Tb	23	0.000	0.003	0.003	0.007	0.003
suicide	Tb	13	0.001	0.005	0.003	0.016	0.004
control	Th	23	0.000	0.000	0.000	0.000	0.000
suicide	Th	13	0.000	0.000	0.000	0.000	0.000
control	Ti	23	24.517	43.348	42.310	69.074	11.212
suicide	Ti	13	22.125	37.912	39.058	52.175	8.352
control	Tl	23	0.000	0.003	0.000	0.071	0.015
suicide	Tl	13	0.000	0.000	0.000	0.000	0.000
control	Tm	23	0.000	0.010	0.010	0.026	0.008
suicide	Tm	13	0.001	0.007	0.007	0.019	0.005
control	U	23	0.000	0.011	0.000	0.102	0.023
suicide	U	13	0.000	0.007	0.000	0.050	0.015
control	V	23	0.882	2.780	2.039	8.632	2.190
suicide	V	13	0.368	1.680	1.142	4.922	1.325
control	Yb	23	0.000	0.013	0.006	0.056	0.018
suicide	Yb	13	0.000	0.013	0.000	0.054	0.019
control	Zn	23	6650.399	15008.972	11437.048	66257.674	12004.918
suicide	Zn	13	7615.167	12272.248	10283.665	23868.270	5129.870
control	Zr	23	0.000	0.777	0.433	2.767	0.852
suicide	Zr	13	0.000	0.328	0.265	0.904	0.326

VII.superior longitudinal fasciculus of brain (G)

Element	Control mean	Suicide mean	Dunn statistic	p
Ag	16.130	22.692	1.795	0.073
Al	20.130	15.615	−1.235	0.217
As	17.000	21.154	2.370	0.018
As2	17.500	20.269	1.908	0.056
Ba	20.565	14.846	−1.564	0.118
Be	17.739	19.846	1.202	0.229
Bi	18.022	19.346	0.363	0.717
Ca	19.522	16.692	−0.774	0.439
Cd	20.130	15.615	−1.235	0.217
Ce	19.609	16.538	−0.840	0.401
Co	18.022	19.346	0.373	0.709
Cr	18.522	18.462	−0.016	0.987
Cs	18.652	18.231	−0.115	0.908
Cu	17.391	20.462	0.840	0.401
Dy	19.283	17.115	−0.594	0.552
Er	20.543	14.885	−1.565	0.118
Eu	17.130	20.923	1.046	0.296
Fe	18.609	18.308	−0.082	0.934

Ga	17.391	20.462	0.938	0.348
Gd	19.522	16.692	−0.774	0.439
Hf	18.109	19.192	0.543	0.587
Hg	19.391	16.923	−0.675	0.500
Hg2	19.478	16.769	−0.741	0.459
Ho	19.109	17.423	−0.550	0.583
K	19.739	16.308	−0.939	0.348
La	17.913	19.538	0.445	0.657
Mg	20.348	15.231	−1.400	0.162
Mn	19.130	17.385	−0.478	0.633
Mo	18.630	18.269	−0.101	0.920
Na	18.913	17.769	−0.313	0.754
Nd	19.783	16.231	−0.972	0.331
Ni	21.348	13.462	−2.160	0.031
P	19.043	17.538	−0.412	0.681
Pb	19.913	16.000	−1.082	0.279
Pd	18.587	18.346	−0.070	0.944
Pr	19.783	16.231	−0.972	0.331
Pt	18.217	19.000	0.214	0.830
Rb	18.391	18.692	0.082	0.934
Sb	18.304	18.846	0.373	0.709
Se	17.826	19.692	0.510	0.610

Se2	18.043	19.308	0.346	0.729
Sm	21.217	13.692	−2.066	0.039
Sn	18.043	19.308	0.346	0.729
Sr	19.870	16.077	−1.037	0.300
Tb	16.870	21.385	1.240	0.215
Ti	20.522	14.923	−1.531	0.126
Tl	18.783	18.000	−0.752	0.452
Tm	19.348	17.000	−0.642	0.521
U	19.391	16.923	−0.805	0.421
V	21.130	13.846	−1.993	0.046
Yb	18.978	17.654	−0.379	0.704
Zn	19.565	16.615	−0.807	0.420
Zr	20.478	15.000	−1.510	0.131

8.inferior longitudinal fasciculus of brain (H)

Group	variable	n	min	mean	median	max	sd
control	Ag	23	0.000	3.306	3.079	9.990	2.914
suicide	Ag	14	0.000	5.513	3.887	15.567	5.464
control	Al	23	15.815	311.298	208.402	1507.193	382.005
suicide	Al	14	30.932	164.972	121.349	587.608	148.920
control	As	23	0.000	0.000	0.000	0.000	0.000
suicide	As	14	0.000	0.333	0.000	3.725	1.008

control	As2	23	0.000	0.000	0.000	0.000	0.000
suicide	As2	14	0.000	0.172	0.000	2.415	0.645
control	Ba	23	1.662	12.589	4.128	113.261	23.812
suicide	Ba	14	0.363	5.050	4.665	14.169	3.340
control	Be	23	0.000	0.104	0.000	2.063	0.431
suicide	Be	14	0.000	0.002	0.000	0.024	0.006
control	Bi	23	0.066	4.297	0.616	56.015	12.690
suicide	Bi	14	0.000	0.952	0.395	5.249	1.529
control	Ca	23	44.544	67.651	62.742	153.327	20.316
suicide	Ca	14	44.830	63.141	61.640	110.172	15.964
control	Cd	23	3.038	19.766	16.256	67.532	15.085
suicide	Cd	14	2.603	34.784	14.744	152.155	45.466
control	Ce	23	0.016	0.167	0.164	0.592	0.117
suicide	Ce	14	0.003	0.524	0.210	2.895	0.775
control	Co	23	0.000	1.682	1.474	10.825	2.258
suicide	Co	14	0.000	1.212	1.521	2.950	1.159
control	Cr	23	0.000	11.236	7.244	50.831	13.076
suicide	Cr	14	0.000	18.852	6.626	84.065	24.470
control	Cs	23	1.709	6.834	7.802	12.124	2.683
suicide	Cs	14	2.789	7.023	6.604	12.960	2.842
control	Cu	23	1818.653	3569.430	3168.228	6919.096	1277.237
suicide	Cu	14	1178.506	3374.602	3622.874	4784.811	992.575
control	Dy	23	0.000	0.011	0.006	0.055	0.013
suicide	Dy	14	0.000	0.007	0.005	0.021	0.007
control	Er	23	0.000	0.047	0.020	0.144	0.049
suicide	Er	14	0.000	0.017	0.008	0.074	0.025
control	Eu	23	0.000	0.011	0.008	0.040	0.011

suicide	Eu	14	0.000	0.008	0.005	0.030	0.009
control	Fe	23	29523.256	57502.180	48941.825	107557.148	25365.981
suicide	Fe	14	29919.603	64645.156	47585.772	115187.095	32147.192
control	Ga	23	0.000	0.105	0.006	0.884	0.205
suicide	Ga	14	0.000	0.038	0.000	0.144	0.056
control	Gd	23	0.002	0.028	0.028	0.084	0.019
suicide	Gd	14	0.000	0.096	0.029	0.897	0.233
control	Hf	23	0.000	0.000	0.000	0.010	0.002
suicide	Hf	14	0.000	0.001	0.000	0.009	0.002
control	Hg	23	0.806	2.426	2.255	5.387	1.175
suicide	Hg	14	0.748	2.068	1.780	4.856	1.071
control	Hg2	23	0.810	2.419	2.345	5.404	1.182
suicide	Hg2	14	0.789	2.093	1.903	5.074	1.087
control	Ho	23	0.000	0.013	0.000	0.079	0.022
suicide	Ho	14	0.000	0.006	0.000	0.024	0.010
control	K	23	1549.681	2753.319	2714.476	3777.064	451.209
suicide	K	14	1165.396	2569.313	2536.307	3597.768	514.073
control	La	23	0.001	0.099	0.101	0.349	0.075
suicide	La	14	0.034	0.355	0.123	2.003	0.538
control	Mg	23	71.581	132.748	135.760	198.886	32.217
suicide	Mg	14	58.988	117.682	117.764	180.686	35.645
control	Mn	23	153.465	347.902	333.605	506.755	92.350
suicide	Mn	14	198.787	321.727	307.813	598.835	103.483
control	Mo	23	0.000	37.865	14.858	146.886	48.014
suicide	Mo	14	0.000	85.583	52.286	321.168	108.207
control	Na	23	953.020	1503.239	1428.453	2352.452	319.309
suicide	Na	14	1141.961	1478.461	1469.798	1921.642	209.597

control	Nd	23	0.000	0.060	0.058	0.130	0.041
suicide	Nd	14	0.012	0.078	0.047	0.349	0.086
control	Ni	23	0.000	6.033	2.879	25.078	6.622
suicide	Ni	14	0.000	3.147	2.200	8.610	2.989
control	P	23	1906.373	4180.370	4209.228	6117.763	855.349
suicide	P	14	1452.187	4003.357	4260.988	5779.851	1059.791
control	Pb	23	0.000	2.831	1.644	21.744	4.645
suicide	Pb	14	0.000	1.978	1.483	4.220	1.533
control	Pd	23	0.000	0.054	0.003	0.260	0.079
suicide	Pd	14	0.000	0.101	0.045	0.423	0.134
control	Pr	23	0.000	0.015	0.011	0.057	0.014
suicide	Pr	14	0.000	0.030	0.016	0.144	0.041
control	Pt	23	0.000	0.038	0.017	0.348	0.070
suicide	Pt	14	0.000	0.118	0.022	1.337	0.352
control	Rb	23	1028.627	2357.634	2539.685	3754.948	685.543
suicide	Rb	14	1066.045	2275.895	2397.439	3094.103	502.275
control	Sb	23	0.000	0.026	0.000	0.402	0.090
suicide	Sb	14	0.000	0.000	0.000	0.000	0.000
control	Se	23	97.705	132.015	128.530	164.617	18.776
suicide	Se	14	60.205	135.719	141.680	180.209	31.172
control	Se2	23	95.601	120.676	117.890	163.905	18.044
suicide	Se2	14	49.534	121.821	123.233	159.233	29.420
control	Sm	23	0.000	0.014	0.008	0.063	0.017
suicide	Sm	14	0.000	0.013	0.007	0.066	0.018
control	Sn	23	0.000	1.213	0.922	3.120	1.043
suicide	Sn	14	0.000	1.098	0.801	3.953	1.101
control	Sr	23	42.175	178.748	144.855	557.536	118.071

suicide	Sr	14	70.582	278.813	129.139	1558.349	397.870
control	Tb	23	0.000	0.005	0.005	0.014	0.004
suicide	Tb	14	0.000	0.004	0.004	0.010	0.003
control	Th	23	0.000	0.000	0.000	0.000	0.000
suicide	Th	14	0.000	0.000	0.000	0.000	0.000
control	Ti	23	21.474	42.213	38.949	81.828	15.167
suicide	Ti	14	17.240	35.739	33.722	59.381	10.346
control	Tl	23	0.000	0.000	0.000	0.000	0.000
suicide	Tl	14	0.000	0.000	0.000	0.000	0.000
control	Tm	23	0.000	0.009	0.006	0.024	0.007
suicide	Tm	14	0.000	0.006	0.004	0.021	0.007
control	U	23	0.000	0.019	0.000	0.261	0.055
suicide	U	14	0.000	0.034	0.000	0.251	0.081
control	V	23	0.475	1.863	1.832	2.911	0.731
suicide	V	14	0.553	1.838	1.804	3.641	0.951
control	Yb	23	0.000	0.015	0.013	0.080	0.019
suicide	Yb	14	0.000	0.019	0.008	0.053	0.020
control	Zn	23	6209.208	13569.335	9223.567	59556.481	11155.515
suicide	Zn	14	6735.517	12422.049	10341.474	24208.856	5115.906
control	Zr	23	0.000	0.465	0.485	0.910	0.292
suicide	Zr	14	0.000	0.620	0.279	2.140	0.737

VIII.inferior longitudinal fasciculus of brain (H)

Element	Control mean	Suicide mean	Dunn statistic	p
Ag	17.739	21.071	0.909	0.363
Al	20.652	16.286	−1.190	0.234
As	18.000	20.643	1.838	0.066
As2	18.500	19.821	1.282	0.200
Ba	19.348	18.429	−0.251	0.802
Be	19.478	18.214	−0.639	0.523
Bi	21.043	15.643	−1.473	0.141
Ca	20.087	17.214	−0.783	0.434
Cd	18.870	19.214	0.094	0.925
Ce	17.609	21.286	1.002	0.316
Co	19.043	18.929	−0.032	0.974
Cr	18.217	20.286	0.566	0.572
Cs	19.043	18.929	−0.031	0.975
Cu	18.913	19.143	0.063	0.950
Dy	19.543	18.107	−0.392	0.695
Er	21.457	14.964	−1.787	0.074
Eu	19.283	18.536	−0.204	0.838
Fe	18.478	19.857	0.376	0.707

Ga	20.217	17.000	−0.932	0.351
Gd	18.304	20.143	0.501	0.616
Hf	18.826	19.286	0.320	0.749
Hg	20.478	16.571	−1.065	0.287
Hg2	20.261	16.929	−0.908	0.364
Ho	18.804	19.321	0.159	0.874
K	20.870	15.929	−1.347	0.178
La	17.217	21.929	1.284	0.199
Mg	20.870	15.929	−1.347	0.178
Mn	20.609	16.357	−1.159	0.247
Mo	17.348	21.714	1.223	0.221
Na	18.957	19.071	0.031	0.975
Nd	18.652	19.571	0.251	0.802
Ni	21.022	15.679	−1.456	0.145
P	19.304	18.500	−0.219	0.826
Pb	18.522	19.786	0.346	0.730
Pd	17.630	21.250	1.029	0.304
Pr	18.370	20.036	0.455	0.649
Pt	18.826	19.286	0.125	0.900
Rb	19.826	17.643	−0.595	0.552
Sb	19.913	17.500	−1.389	0.165
Se	18.174	20.357	0.595	0.552

Se2	18.391	20.000	0.438	0.661
Sm	19.304	18.500	−0.221	0.825
Sn	19.565	18.071	−0.408	0.684
Sr	19.043	18.929	−0.031	0.975
Tb	19.957	17.429	−0.690	0.490
Ti	20.652	16.286	−1.190	0.234
Tm	20.609	16.357	−1.160	0.246
U	18.978	19.036	0.018	0.985
V	19.522	18.143	−0.376	0.707
Yb	17.783	21.000	0.889	0.374
Zn	18.348	20.071	0.470	0.639
Zr	19.522	18.143	−0.376	0.707

9.dorsal thalamus (I)

Group	variable	n	min	mean	median	max	sd
control	Ag	21	0.000	3.442	2.852	14.194	3.295
suicide	Ag	14	0.379	6.901	6.199	16.768	5.405
control	Al	21	34.328	280.199	147.799	1219.214	283.213
suicide	Al	14	43.237	287.478	159.213	834.199	269.631
control	As	21	0.000	0.000	0.000	0.000	0.000
suicide	As	14	0.000	0.000	0.000	0.000	0.000
control	As2	21	0.000	0.000	0.000	0.000	0.000
suicide	As2	14	0.000	0.000	0.000	0.000	0.000
control	Ba	21	1.398	5.162	4.495	11.511	2.778
suicide	Ba	14	0.703	4.998	3.923	12.234	3.516
control	Be	21	0.000	0.005	0.000	0.096	0.021
suicide	Be	14	0.000	0.004	0.000	0.057	0.015
control	Bi	21	0.000	0.762	0.638	2.608	0.648
suicide	Bi	14	0.000	0.959	0.335	4.361	1.289
control	Ca	21	58.804	75.874	73.150	106.177	14.050
suicide	Ca	14	53.227	63.813	60.488	83.405	10.370
control	Cd	21	5.162	56.759	27.059	360.076	84.062
suicide	Cd	14	4.074	39.863	18.774	189.844	50.943
control	Ce	21	0.038	0.396	0.225	2.365	0.516
suicide	Ce	14	0.049	0.404	0.231	2.709	0.683
control	Co	21	0.000	2.873	2.735	11.894	2.623
suicide	Co	14	0.000	1.580	1.050	4.448	1.734
control	Cr	21	0.000	29.506	6.478	139.171	43.373
suicide	Cr	14	0.000	21.473	7.015	142.817	38.708

Group	variable	n	min	mean	median	max	sd
control	Cs	21	1.447	6.259	5.982	13.774	2.818
suicide	Cs	14	4.124	6.886	6.618	11.997	2.310
control	Cu	21	1284.695	3306.914	3216.830	5815.298	1225.122
suicide	Cu	14	1701.205	3487.271	3739.773	4385.252	818.088
control	Dy	21	0.000	0.013	0.012	0.055	0.013
suicide	Dy	14	0.000	0.010	0.008	0.036	0.011
control	Er	21	0.000	0.044	0.029	0.131	0.045
suicide	Er	14	0.000	0.015	0.000	0.071	0.025
control	Eu	21	0.000	0.005	0.003	0.016	0.005
suicide	Eu	14	0.000	0.007	0.004	0.024	0.007
control	Fe	21	40914.771	75411.577	62759.428	169799.634	37586.239
suicide	Fe	14	45820.403	94377.760	87441.475	158166.153	35587.992
control	Ga	21	0.000	0.098	0.005	0.504	0.155
suicide	Ga	14	0.000	0.100	0.039	0.427	0.139
control	Gd	21	0.006	0.038	0.036	0.132	0.028
suicide	Gd	14	0.000	0.121	0.028	1.107	0.291
control	Hf	21	0.000	0.006	0.000	0.116	0.025
suicide	Hf	14	0.000	0.001	0.000	0.019	0.005
control	Hg	21	0.961	3.764	2.519	17.014	3.541
suicide	Hg	14	0.335	3.175	3.351	6.342	1.919
control	Hg2	21	0.884	3.748	2.473	17.009	3.525
suicide	Hg2	14	0.344	3.134	3.340	6.131	1.887
control	Ho	21	0.000	0.005	0.000	0.034	0.009
suicide	Ho	14	0.000	0.006	0.000	0.041	0.013
control	K	21	1479.388	2462.912	2439.055	4047.021	531.660
suicide	K	14	2130.864	2648.100	2695.797	3169.687	288.570

Group	variable	n	min	mean	median	max	sd
control	La	21	0.013	0.259	0.145	1.468	0.324
suicide	La	14	0.068	0.275	0.105	1.927	0.491
control	Mg	21	55.250	104.042	100.851	153.869	27.990
suicide	Mg	14	72.752	114.202	114.525	152.531	26.595
control	Mn	21	177.123	331.020	335.718	450.677	72.565
suicide	Mn	14	274.571	348.970	332.103	494.417	68.601
control	Mo	21	0.000	222.046	164.493	625.139	165.697
suicide	Mo	14	0.000	196.389	171.788	814.515	189.444
control	Na	21	1100.691	1701.856	1734.780	2114.960	321.344
suicide	Na	14	1363.372	1681.555	1679.968	2103.258	220.529
control	Nd	21	0.000	0.085	0.058	0.290	0.075
suicide	Nd	14	0.000	0.060	0.046	0.206	0.054
control	Ni	21	0.000	3.931	3.349	16.009	3.854
suicide	Ni	14	0.000	3.034	1.688	12.191	3.536
control	P	21	1420.941	2577.168	2558.491	3813.211	604.665
suicide	P	14	2337.676	3144.562	2976.262	4561.866	574.659
control	Pb	21	0.000	3.448	2.300	22.919	4.923
suicide	Pb	14	0.000	2.261	1.514	6.887	2.105
control	Pd	21	0.000	0.099	0.029	0.488	0.130
suicide	Pd	14	0.000	0.030	0.011	0.154	0.046
control	Pr	21	0.000	0.025	0.023	0.102	0.025
suicide	Pr	14	0.000	0.025	0.014	0.144	0.036
control	Pt	21	0.000	0.060	0.024	0.410	0.095
suicide	Pt	14	0.001	0.080	0.016	0.619	0.167
control	Rb	21	781.239	2422.393	2423.726	4113.105	831.267
suicide	Rb	14	1944.499	2539.145	2403.262	3453.652	430.089

Group	variable	n	min	mean	median	max	sd
control	Sb	21	0.000	0.023	0.000	0.223	0.066
suicide	Sb	14	0.000	0.000	0.000	0.000	0.000
control	Se	21	116.914	180.537	183.086	261.581	40.533
suicide	Se	14	132.793	196.907	196.195	248.828	36.871
control	Se2	21	104.647	163.321	169.922	229.819	35.599
suicide	Se2	14	111.768	177.038	187.985	210.621	29.552
control	Sm	21	0.000	0.022	0.019	0.075	0.019
suicide	Sm	14	0.000	0.019	0.013	0.069	0.019
control	Sn	21	0.000	3.742	2.889	11.066	3.353
suicide	Sn	14	0.207	2.630	2.536	6.082	1.929
control	Sr	21	57.263	206.759	187.207	597.498	125.495
suicide	Sr	14	46.195	146.543	124.506	295.940	65.669
control	Tb	21	0.000	0.004	0.003	0.010	0.003
suicide	Tb	14	0.000	0.003	0.002	0.008	0.003
control	Th	21	0.000	0.000	0.000	0.000	0.000
suicide	Th	14	0.000	0.000	0.000	0.000	0.000
control	Ti	21	18.183	28.863	28.089	48.184	7.862
suicide	Ti	14	21.417	31.140	31.693	41.987	5.875
control	Tl	21	0.000	0.008	0.000	0.085	0.025
suicide	Tl	14	0.000	0.005	0.000	0.072	0.019
control	Tm	21	0.000	0.007	0.003	0.025	0.007
suicide	Tm	14	0.000	0.008	0.007	0.021	0.006
control	U	21	0.000	0.050	0.004	0.292	0.077
suicide	U	14	0.000	0.009	0.000	0.039	0.014
control	V	21	0.576	2.759	2.006	14.456	3.174
suicide	V	14	0.577	1.820	1.523	5.277	1.433

Group	variable	n	min	mean	median	max	sd
control	Yb	21	0.000	0.008	0.002	0.040	0.013
suicide	Yb	14	0.000	0.009	0.006	0.026	0.010
control	Zn	21	8942.232	15169.880	13313.351	30276.184	5756.220
suicide	Zn	14	10835.582	17039.134	16183.593	35837.501	6870.247
control	Zr	21	0.000	0.953	0.710	7.240	1.526
suicide	Zr	14	0.000	1.123	0.478	9.644	2.479

IX.dorsal thalamus (I)

Element	Control mean	Suicide mean	Dunn statistic	p
Ag	15.286	22.071	1.919	0.055
Al	17.857	18.214	0.101	0.920
Ba	18.476	17.286	−0.337	0.736
Be	17.857	18.214	0.251	0.802
Bi	19.190	16.214	−0.842	0.400
Ca	21.810	12.286	−2.694	0.007
Cd	18.952	16.571	−0.673	0.501
Ce	18.571	17.143	−0.404	0.686
Co	20.024	14.964	−1.461	0.144
Cr	18.786	16.821	−0.556	0.578
Cs	17.190	19.214	0.572	0.567
Cu	16.810	19.786	0.842	0.400
Dy	19.024	16.464	−0.724	0.469

Er	20.857	13.714	−2.105	0.035
Eu	17.310	19.036	0.491	0.623
Fe	15.429	21.857	1.818	0.069
Ga	17.643	18.536	0.261	0.794
Gd	18.762	16.857	−0.539	0.590
Hf	17.381	18.929	0.901	0.367
Hg	18.095	17.857	−0.067	0.946
Hg2	18.238	17.643	−0.168	0.866
Ho	18.381	17.429	−0.318	0.750
K	15.905	21.143	1.482	0.138
La	19.238	16.143	−0.875	0.381
Mg	16.714	19.929	0.909	0.363
Mn	17.429	18.857	0.404	0.686
Mo	18.738	16.893	−0.522	0.602
Na	18.429	17.357	−0.303	0.762
Nd	19.214	16.179	−0.859	0.391
Ni	18.952	16.571	−0.674	0.500
P	14.095	23.857	2.761	0.006
Pb	18.857	16.714	−0.608	0.543
Pd	19.143	16.286	−0.830	0.407
Pr	18.357	17.464	−0.253	0.800
Pt	19.238	16.143	−0.875	0.381

Rb	17.286	19.071	0.505	0.614
Sb	19.000	16.500	−1.456	0.145
Se	16.048	20.929	1.381	0.167
Se2	16.143	20.786	1.313	0.189
Sm	18.762	16.857	−0.539	0.590
Sn	19.143	16.286	−0.808	0.419
Sr	20.190	14.714	−1.549	0.121
Tb	19.048	16.429	−0.749	0.454
Ti	16.333	20.500	1.179	0.239
Tl	18.214	17.679	−0.312	0.755
Tm	17.095	19.357	0.644	0.520
U	20.190	14.714	−1.628	0.103
V	19.524	15.714	−1.077	0.281
Yb	16.952	19.571	0.772	0.440
Zn	17.000	19.500	0.707	0.480
Zr	18.929	16.607	−0.658	0.510

10.nucleus accumbens septi (J)

Group	variable	n	min	mean	median	max	sd
control	Ag	20	0.000	4.622	3.331	17.774	4.764
suicide	Ag	14	0.000	8.481	6.471	25.878	8.661
control	Al	20	34.631	380.487	233.352	1291.516	355.716

suicide	Al	14	5.497	165.257	107.539	929.546	230.909
control	As	20	0.000	0.000	0.000	0.000	0.000
suicide	As	14	0.000	0.000	0.000	0.000	0.000
control	As2	20	0.000	0.000	0.000	0.000	0.000
suicide	As2	14	0.000	0.000	0.000	0.000	0.000
control	Ba	20	0.385	7.260	5.973	27.617	5.753
suicide	Ba	14	0.287	6.480	5.283	16.302	5.470
control	Be	20	0.000	0.033	0.000	0.666	0.149
suicide	Be	14	0.000	0.056	0.000	0.739	0.197
control	Bi	20	0.000	23.371	1.175	438.907	97.816
suicide	Bi	14	0.000	1.978	1.244	8.369	2.578
control	Ca	20	31.953	81.440	76.397	151.434	25.105
suicide	Ca	14	46.024	66.035	64.715	90.596	12.042
control	Cd	20	12.242	63.671	25.708	550.571	118.172
suicide	Cd	14	5.461	28.661	19.527	87.259	25.407
control	Ce	20	0.053	0.805	0.267	7.790	1.721
suicide	Ce	14	0.000	0.260	0.124	0.679	0.254
control	Co	20	0.000	2.592	2.434	11.972	2.701
suicide	Co	14	0.000	2.139	2.291	4.876	1.846
control	Cr	20	0.000	32.046	12.210	190.170	46.368
suicide	Cr	14	0.000	20.719	15.288	63.313	21.838
control	Cs	20	1.558	6.721	6.712	15.175	3.426
suicide	Cs	14	3.719	7.857	7.095	15.643	3.397
control	Cu	20	2470.552	5174.663	5104.892	7500.748	1508.661
suicide	Cu	14	3152.582	5728.310	5464.588	8040.343	1433.772
control	Dy	20	0.000	0.021	0.015	0.097	0.023
suicide	Dy	14	0.000	0.012	0.009	0.037	0.012

control	Er	20	0.000	0.062	0.028	0.207	0.076
suicide	Er	14	0.000	0.052	0.026	0.272	0.077
control	Eu	20	0.000	0.016	0.013	0.084	0.021
suicide	Eu	14	0.000	0.010	0.007	0.030	0.010
control	Fe	20	37721.151	138745.908	125748.536	266788.358	75534.354
suicide	Fe	14	89556.670	167414.351	167532.699	286249.785	54452.148
control	Ga	20	0.000	0.092	0.000	0.854	0.205
suicide	Ga	14	0.000	0.092	0.006	0.417	0.142
control	Gd	20	0.000	0.171	0.033	2.378	0.523
suicide	Gd	14	0.000	0.051	0.015	0.413	0.108
control	Hf	20	0.000	0.012	0.000	0.179	0.041
suicide	Hf	14	0.000	0.000	0.000	0.000	0.000
control	Hg	20	1.092	5.062	3.449	19.851	4.509
suicide	Hg	14	0.568	4.903	5.775	7.651	2.400
control	Hg2	20	1.012	5.044	3.602	19.836	4.465
suicide	Hg2	14	0.602	4.970	5.722	7.477	2.357
control	Ho	20	0.000	0.009	0.000	0.046	0.015
suicide	Ho	14	0.000	0.014	0.001	0.057	0.020
control	K	20	1262.094	2979.797	3171.302	4054.377	808.856
suicide	K	14	2155.899	3321.459	3353.237	4651.435	571.976
control	La	20	0.028	0.545	0.179	5.554	1.224
suicide	La	14	0.043	0.165	0.138	0.366	0.110
control	Mg	20	79.588	123.178	117.270	190.122	34.879
suicide	Mg	14	71.165	128.636	124.504	168.337	27.182
control	Mn	20	204.018	454.849	463.115	712.515	146.753
suicide	Mn	14	221.101	494.683	488.833	963.727	174.733
control	Mo	20	10.248	495.675	416.867	1064.759	294.534

suicide	Mo	14	0.000	546.650	572.818	905.926	243.402
control	Na	20	987.231	1602.158	1626.938	2313.159	372.048
suicide	Na	14	1063.954	1706.405	1506.270	2392.554	441.118
control	Nd	20	0.000	0.169	0.093	0.962	0.220
suicide	Nd	14	0.021	0.090	0.072	0.206	0.058
control	Ni	20	0.000	7.003	5.430	18.073	6.120
suicide	Ni	14	0.000	5.953	4.133	23.040	6.422
control	P	20	1342.569	2607.000	2619.692	4130.273	730.045
suicide	P	14	2039.396	2904.055	2814.444	3610.649	468.130
control	Pb	20	0.000	4.984	5.065	12.446	4.233
suicide	Pb	14	0.000	2.935	2.777	7.492	2.412
control	Pd	20	0.000	0.170	0.116	0.562	0.176
suicide	Pd	14	0.000	0.111	0.075	0.340	0.121
control	Pr	20	0.000	0.050	0.031	0.304	0.071
suicide	Pr	14	0.000	0.021	0.013	0.081	0.024
control	Pt	20	0.000	0.202	0.042	3.165	0.699
suicide	Pt	14	0.004	1.376	0.030	14.175	3.815
control	Rb	20	782.655	2600.214	2770.424	4271.733	1005.821
suicide	Rb	14	1448.964	2942.547	3028.926	3925.558	681.132
control	Sb	20	0.000	0.018	0.000	0.223	0.054
suicide	Sb	14	0.000	0.003	0.000	0.036	0.010
control	Se	20	80.751	165.387	171.501	228.878	45.142
suicide	Se	14	128.481	199.225	201.062	281.910	36.239
control	Se2	20	65.986	152.756	150.896	238.804	46.930
suicide	Se2	14	99.222	172.958	176.753	264.425	37.656
control	Sm	20	0.000	0.031	0.022	0.093	0.027
suicide	Sm	14	0.000	0.014	0.012	0.070	0.019

control	Sn	20	0.000	2.263	1.675	9.453	2.309
suicide	Sn	14	0.000	2.215	1.841	5.475	1.794
control	Sr	20	82.256	298.970	168.048	1186.765	319.378
suicide	Sr	14	97.489	232.995	209.165	770.506	171.586
control	Tb	20	0.000	0.011	0.008	0.041	0.010
suicide	Tb	14	0.000	0.004	0.004	0.012	0.005
control	Th	20	0.000	0.003	0.000	0.061	0.014
suicide	Th	14	0.000	0.000	0.000	0.000	0.000
control	Ti	20	11.923	34.093	31.814	69.340	15.450
suicide	Ti	14	22.088	33.750	34.756	44.523	7.212
control	Tl	20	0.000	0.000	0.000	0.000	0.000
suicide	Tl	14	0.000	0.000	0.000	0.000	0.000
control	Tm	20	0.000	0.019	0.015	0.079	0.020
suicide	Tm	14	0.000	0.012	0.010	0.030	0.010
control	U	20	0.000	0.081	0.000	0.753	0.192
suicide	U	14	0.000	0.029	0.000	0.288	0.076
control	V	20	0.578	7.949	2.977	99.812	21.661
suicide	V	14	0.557	3.471	2.321	18.785	4.668
control	Yb	20	0.000	0.022	0.020	0.073	0.025
suicide	Yb	14	0.000	0.026	0.000	0.123	0.043
control	Zn	20	7022.948	17005.958	15681.175	34693.495	6842.212
suicide	Zn	14	10535.666	19286.004	17333.404	31603.630	6238.854
control	Zr	20	0.000	2.073	0.926	13.576	3.283
suicide	Zr	14	0.000	0.558	0.208	2.397	0.772

X.nucleus accumbens septi (J)

Element	Control mean	Suicide mean	Dunn statistic	p
Ag	15.600	20.214	1.330	0.183
Element	Control mean	Suicide mean	Dunn statistic	p
Al	21.250	12.143	−2.624	0.009
Ba	18.500	16.071	−0.700	0.484
Be	16.850	18.429	0.924	0.355
Bi	18.150	16.571	−0.455	0.649
Ca	20.750	12.857	−2.275	0.023
Cd	19.350	14.857	−1.295	0.195
Ce	19.550	14.571	−1.435	0.151
Co	17.675	17.250	−0.124	0.901
Cr	18.050	16.714	−0.387	0.699
Cs	16.400	19.071	0.770	0.441
Cu	15.950	19.714	1.085	0.278
Dy	19.000	15.357	−1.057	0.291
Er	18.000	16.786	−0.360	0.719
Eu	18.175	16.536	−0.478	0.632
Fe	15.650	20.143	1.295	0.195
Ga	16.500	18.929	0.758	0.448
Gd	19.800	14.214	−1.610	0.107
Hf	18.550	16.000	−1.493	0.135

Hg	16.700	18.643	0.560	0.576
Hg2	16.500	18.929	0.700	0.484
Ho	16.100	19.500	1.078	0.281
K	15.700	20.071	1.260	0.208
La	19.150	15.143	−1.155	0.248
Mg	16.100	19.500	0.980	0.327
Mn	16.800	18.500	0.490	0.624
Mo	16.250	19.286	0.875	0.382
Na	16.450	19.000	0.735	0.462
Nd	18.650	15.857	−0.805	0.421
Ni	18.650	15.857	−0.805	0.421
P	15.500	20.357	1.400	0.162
Pb	19.050	15.286	−1.089	0.276
Pd	18.950	15.429	−1.018	0.309
Pr	19.625	14.464	−1.491	0.136
Pt	17.600	17.357	−0.070	0.944
Rb	16.250	19.286	0.875	0.382
Sb	18.125	16.607	−0.782	0.434
Se	14.650	21.571	1.995	0.046
Se2	15.850	19.857	1.155	0.248
Sm	20.500	13.214	−2.105	0.035
Sn	17.150	18.000	0.245	0.806

Sr	17.150	18.000	0.245	0.806
Tb	21.000	12.500	−2.460	0.014
Th	17.850	17.000	−0.837	0.403
Ti	16.850	18.429	0.455	0.649
Tm	19.000	15.357	−1.054	0.292
U	18.000	16.786	−0.385	0.700
V	20.000	13.929	−1.750	0.080
Yb	18.300	16.357	−0.591	0.554
Zn	15.900	19.786	1.120	0.263
Zr	20.600	13.071	−2.179	0.029

11.insula (K)

Group	variable	n	min	mean	median	max	sd
control	Ag	18	0.000	5.145	5.507	14.240	3.718
suicide	Ag	13	0.000	8.693	7.096	23.331	8.397
control	Al	18	17.168	196.118	141.305	648.807	165.272
suicide	Al	13	32.032	260.691	127.976	1522.535	390.853
control	As	18	0.000	0.058	0.000	1.038	0.245
suicide	As	13	0.000	0.000	0.000	0.000	0.000
control	As2	18	0.000	0.000	0.000	0.000	0.000
suicide	As2	13	0.000	0.000	0.000	0.000	0.000
control	Ba	18	0.831	4.816	3.532	10.823	3.068
suicide	Ba	13	0.372	4.946	4.280	12.704	3.278

control	Be	18	0.000	0.015	0.000	0.269	0.063
suicide	Be	13	0.000	0.008	0.000	0.051	0.019
control	Bi	18	0.002	0.895	0.973	2.402	0.606
suicide	Bi	13	0.000	5.161	0.797	58.479	16.033
control	Ca	18	54.207	71.159	68.054	102.524	13.565
suicide	Ca	13	43.312	61.674	60.434	89.533	12.148
control	Cd	18	6.314	39.304	22.846	276.182	62.126
suicide	Cd	13	3.792	29.775	18.944	89.502	27.847
control	Ce	18	0.000	0.459	0.134	5.553	1.281
suicide	Ce	13	0.048	0.457	0.127	2.360	0.713
control	Co	18	0.000	2.510	1.997	9.460	2.534
suicide	Co	13	0.000	1.457	1.799	3.932	1.334
control	Cr	18	0.000	19.341	8.813	156.705	37.505
suicide	Cr	13	0.000	14.127	3.009	62.366	19.483
control	Cs	18	1.008	5.719	5.601	12.244	2.843
suicide	Cs	13	2.927	5.495	5.059	13.452	2.786
control	Cu	18	1978.271	4178.497	4131.662	6550.309	1155.310
suicide	Cu	13	2548.483	4267.988	4308.146	5626.397	960.986
control	Dy	18	0.000	0.007	0.004	0.025	0.009
suicide	Dy	13	0.001	0.018	0.014	0.041	0.013
control	Er	18	0.000	0.060	0.048	0.203	0.063
suicide	Er	13	0.000	0.064	0.057	0.218	0.060
control	Eu	18	0.000	0.009	0.007	0.023	0.007
suicide	Eu	13	0.000	0.009	0.009	0.028	0.009
control	Fe	18	33160.413	55359.690	51751.027	120821.693	20492.983
suicide	Fe	13	35424.614	55368.495	48680.269	106612.135	19968.355
control	Ga	18	0.000	0.177	0.118	0.982	0.258

suicide	Ga	13	0.000	0.100	0.006	0.517	0.174
control	Gd	18	0.000	0.281	0.021	4.657	1.092
suicide	Gd	13	0.000	0.051	0.022	0.206	0.062
control	Hf	18	0.000	0.009	0.000	0.151	0.035
suicide	Hf	13	0.000	0.000	0.000	0.000	0.000
control	Hg	18	0.497	4.207	4.116	12.357	3.110
suicide	Hg	13	0.903	2.834	2.750	6.012	1.503
control	Hg2	18	0.501	4.213	4.105	12.481	3.131
suicide	Hg2	13	0.963	2.836	2.625	6.093	1.509
control	Ho	18	0.000	0.013	0.000	0.049	0.018
suicide	Ho	13	0.000	0.006	0.000	0.028	0.009
control	K	18	2207.393	2712.748	2672.222	3387.484	375.969
suicide	K	13	2083.543	2735.412	2824.040	3334.853	382.974
control	La	18	0.000	0.291	0.071	3.676	0.853
suicide	La	13	0.031	0.274	0.067	1.411	0.438
control	Mg	18	77.198	99.615	96.787	140.558	16.233
suicide	Mg	13	62.445	102.324	96.913	143.132	23.426
control	Mn	18	161.130	231.839	218.097	448.258	63.834
suicide	Mn	13	137.413	200.212	201.599	320.983	44.345
control	Mo	18	66.091	228.206	162.156	1484.332	319.941
suicide	Mo	13	17.399	175.241	162.091	349.367	103.071
control	Na	18	1124.208	1785.257	1692.984	2744.234	465.118
suicide	Na	13	1345.303	1831.662	1847.461	2230.818	251.823
control	Nd	18	0.000	0.093	0.054	0.658	0.148
suicide	Nd	13	0.000	0.084	0.073	0.386	0.100
control	Ni	18	0.000	5.410	4.847	16.019	4.671
suicide	Ni	13	0.000	2.922	1.273	14.445	4.074

control	P	18	1507.681	2153.199	2118.940	2999.538	334.914
suicide	P	13	1657.775	2282.412	2210.509	3024.818	429.799
control	Pb	18	0.000	2.869	2.629	11.155	3.003
suicide	Pb	13	0.000	1.275	1.161	4.289	1.321
control	Pd	18	0.000	0.039	0.017	0.136	0.046
suicide	Pd	13	0.000	0.057	0.000	0.261	0.080
control	Pr	18	0.000	0.037	0.020	0.321	0.072
suicide	Pr	13	0.000	0.027	0.020	0.099	0.029
control	Pt	18	0.000	0.035	0.022	0.241	0.055
suicide	Pt	13	0.007	5.137	0.033	36.602	12.233
control	Rb	18	789.080	2227.851	2270.232	3378.027	709.833
suicide	Rb	13	1422.143	2110.127	2107.329	3418.519	494.332
control	Sb	18	0.000	0.018	0.000	0.323	0.076
suicide	Sb	13	0.000	0.000	0.000	0.000	0.000
control	Se	18	107.837	152.585	152.072	211.961	24.063
suicide	Se	13	116.697	148.756	143.117	181.190	21.082
control	Se2	18	81.506	137.727	133.743	186.463	26.075
suicide	Se2	13	97.648	131.586	128.793	164.628	21.114
control	Sm	18	0.000	0.012	0.012	0.041	0.010
suicide	Sm	13	0.000	0.012	0.012	0.029	0.010
control	Sn	18	0.000	1.430	1.419	4.525	1.248
suicide	Sn	13	0.000	1.190	1.029	3.829	1.069
control	Sr	18	47.242	156.956	139.100	357.767	89.365
suicide	Sr	13	49.217	180.476	135.447	572.009	143.224
control	Tb	18	0.000	0.006	0.006	0.020	0.005
suicide	Tb	13	0.000	0.004	0.003	0.011	0.004
control	Th	18	0.000	0.000	0.000	0.000	0.000

suicide	Th	13	0.000	0.000	0.000	0.000	0.000
control	Ti	18	10.841	25.901	26.768	42.899	8.908
suicide	Ti	13	15.818	28.439	26.826	53.523	10.316
control	Tl	18	0.000	0.006	0.000	0.107	0.025
suicide	Tl	13	0.000	0.000	0.000	0.000	0.000
control	Tm	18	0.000	0.010	0.010	0.028	0.007
suicide	Tm	13	0.000	0.011	0.013	0.028	0.010
control	U	18	0.000	0.014	0.000	0.086	0.026
suicide	U	13	0.000	0.049	0.000	0.365	0.103
control	V	18	0.976	2.328	1.748	9.100	1.831
suicide	V	13	0.730	5.170	2.804	26.632	7.049
control	Yb	18	0.000	0.015	0.007	0.064	0.020
suicide	Yb	13	0.000	0.021	0.022	0.053	0.019
control	Zn	18	12178.182	17560.598	16457.442	27844.842	4749.844
suicide	Zn	13	12636.993	19034.616	15184.662	31963.993	6651.272
control	Zr	18	0.000	0.814	0.385	8.529	1.956
suicide	Zr	13	0.000	0.742	0.325	3.406	0.951

XI.insula (K)

Element	Control mean	Suicide mean	Dunn statistic	p
Ag	14.583	17.962	1.021	0.307
Al	16.000	16.000	0.000	1.000
As	16.361	15.500	−0.850	0.395
Ba	15.833	16.231	0.120	0.904
Be	15.417	16.808	0.819	0.413
Bi	16.944	14.692	−0.681	0.496
Ca	18.889	12.000	−2.082	0.037
Cd	16.500	15.308	−0.360	0.719
Ce	14.833	17.615	0.841	0.401
Co	17.389	14.077	−1.013	0.311
Cr	16.444	15.385	−0.322	0.747
Cs	16.833	14.846	−0.600	0.548
Cu	15.500	16.692	0.360	0.719
Dy	12.556	20.769	2.513	0.012
Er	15.167	17.154	0.603	0.547
Eu	16.250	15.654	−0.180	0.857
Fe	15.944	16.077	0.040	0.968
Ga	17.333	14.154	−0.983	0.326
Gd	14.861	17.577	0.821	0.411

Hf	16.722	15.000	−1.222	0.222
Hg	17.667	13.692	−1.201	0.230
Hg2	17.722	13.615	−1.241	0.215
Ho	16.778	14.923	−0.613	0.540
K	15.722	16.385	0.200	0.841
La	15.667	16.462	0.240	0.810
Mg	15.667	16.462	0.240	0.810
Mn	18.667	12.308	−1.922	0.055
Mo	15.556	16.615	0.320	0.749
Na	14.944	17.462	0.761	0.447
Nd	16.167	15.769	−0.120	0.904
Ni	18.528	12.500	−1.823	0.068
P	14.944	17.462	0.761	0.447
Pb	18.139	13.038	−1.568	0.117
Pd	16.056	15.923	−0.042	0.967
Pr	16.111	15.846	−0.080	0.936
Pt	13.222	19.846	2.002	0.045
Rb	16.944	14.692	−0.681	0.496
Sb	16.361	15.500	−0.850	0.395
Se	16.667	15.077	−0.480	0.631
Se2	17.167	14.385	−0.841	0.401
Sm	15.806	16.269	0.141	0.888

Sn	16.667	15.077	−0.481	0.630
Sr	15.667	16.462	0.240	0.810
Tb	17.444	14.000	−1.045	0.296
Ti	15.556	16.615	0.320	0.749
Tl	16.361	15.500	−0.850	0.395
Tm	15.611	16.538	0.281	0.779
U	14.944	17.462	0.867	0.386
V	14.222	18.462	1.281	0.200
Yb	14.611	17.923	1.009	0.313
Zn	15.500	16.692	0.360	0.719
Zr	15.639	16.500	0.261	0.794

12. liver (L)

Group	variable	n	min	mean	median	max	sd
control	Ag	22	0.000	12.248	1.583	191.169	40.433
suicide	Ag	13	0.580	11.659	4.546	88.455	23.687
control	Al	22	41.278	317.294	223.921	1137.002	260.328
suicide	Al	13	38.608	354.421	388.144	982.118	269.126
control	As	22	0.000	3.339	0.449	23.952	6.158
suicide	As	13	0.000	1.433	0.000	7.731	2.305
control	As2	22	0.000	2.600	0.000	20.988	5.410
suicide	As2	13	0.000	1.089	0.000	6.402	1.863
control	Ba	22	1.286	5.161	3.769	18.330	3.643
suicide	Ba	13	0.920	4.638	3.757	11.750	3.112
control	Be	22	0.000	0.027	0.000	0.244	0.062
suicide	Be	13	0.000	0.036	0.000	0.292	0.082
control	Bi	22	0.000	6.019	0.989	87.452	18.766
suicide	Bi	13	0.000	4.858	0.530	55.644	15.269
control	Ca	22	28.331	61.241	53.037	150.488	31.613
suicide	Ca	13	39.851	57.355	57.875	80.583	11.882
control	Cd	22	261.225	1352.775	892.928	6388.117	1365.454
suicide	Cd	13	85.056	1444.723	1022.055	2962.927	936.075
control	Ce	22	1.256	19.970	9.313	147.921	31.525

suicide	Ce	13	0.509	29.934	23.141	78.497	23.005
control	Co	22	2.667	35.285	29.590	94.742	24.045
suicide	Co	13	3.028	27.626	25.975	53.735	15.689
control	Cr	22	0.000	8.517	4.244	43.875	10.319
suicide	Cr	13	0.000	18.369	7.744	121.061	32.333
control	Cs	22	4.177	8.857	8.021	21.550	4.170
suicide	Cs	13	4.274	9.526	8.484	18.450	4.684
control	Cu	22	1092.651	4892.847	4374.574	25320.294	4884.397
suicide	Cu	13	2276.768	4765.193	4787.786	6856.578	1448.561
control	Dy	22	0.003	0.053	0.047	0.202	0.049
suicide	Dy	13	0.012	0.069	0.052	0.221	0.053
control	Er	22	0.000	0.028	0.025	0.073	0.024
suicide	Er	13	0.000	0.043	0.038	0.119	0.037
control	Eu	22	0.001	0.022	0.018	0.087	0.020
suicide	Eu	13	0.004	0.037	0.036	0.108	0.025
control	Fe	22	48363.701	214732.619	166606.441	550601.754	148975.665
suicide	Fe	13	91458.676	276441.495	240253.777	751844.162	165001.946
control	Ga	22	0.000	1.244	0.495	8.113	1.940
suicide	Ga	13	0.000	1.904	2.246	4.425	1.386
control	Gd	22	0.066	1.816	0.316	26.437	5.583
suicide	Gd	13	0.067	1.137	0.951	2.528	0.812
control	Hf	22	0.000	0.014	0.000	0.147	0.033

suicide	Hf	13	0.000	0.037	0.000	0.399	0.109
control	Hg	22	2.456	35.835	23.218	123.754	36.790
suicide	Hg	13	5.044	27.410	26.069	54.783	17.818
control	Hg2	22	2.456	35.669	23.216	123.020	36.649
suicide	Hg2	13	4.952	27.352	25.826	55.275	17.954
control	Ho	22	0.000	0.008	0.000	0.036	0.011
suicide	Ho	13	0.000	0.010	0.002	0.060	0.020
control	K	22	1142.525	1865.809	1895.056	2378.933	372.189
suicide	K	13	1470.198	2014.333	2013.158	2500.399	310.430
control	La	22	0.882	12.106	5.759	80.111	17.430
suicide	La	13	0.295	17.801	14.964	40.157	12.408
control	Mg	22	46.500	97.500	93.991	160.051	30.994
suicide	Mg	13	83.181	119.076	113.514	180.098	26.477
control	Mn	22	543.898	1090.833	1131.330	1899.042	336.325
suicide	Mn	13	712.661	1175.415	1221.865	1739.616	287.181
control	Mo	22	1296.969	6158.721	6092.869	16113.281	3773.103
suicide	Mo	13	1944.120	6516.164	6828.324	10143.657	2695.205
control	Na	22	565.210	939.681	919.654	2057.614	335.728
suicide	Na	13	539.343	998.906	1038.287	1247.740	188.698
control	Nd	22	0.225	2.128	1.393	12.445	2.706
suicide	Nd	13	0.117	3.780	3.044	9.385	2.722
control	Ni	22	0.000	3.507	2.617	20.921	4.360

suicide	Ni	13	0.000	3.189	3.011	11.389	3.151
control	P	22	1472.424	2107.125	1995.386	3092.355	404.866
suicide	P	13	1676.560	2214.519	2134.111	2771.774	356.940
control	Pb	22	11.523	46.451	36.566	175.125	37.070
suicide	Pb	13	14.301	44.372	26.221	103.299	30.019
control	Pd	22	0.000	0.153	0.067	1.091	0.238
suicide	Pd	13	0.000	0.066	0.062	0.188	0.064
control	Pr	22	0.084	0.946	0.535	5.796	1.274
suicide	Pr	13	0.046	1.649	1.445	4.854	1.303
control	Pt	22	0.000	0.044	0.012	0.285	0.077
suicide	Pt	13	0.001	0.086	0.013	0.877	0.239
control	Rb	22	1485.732	2969.240	2698.379	4953.381	964.906
suicide	Rb	13	2202.878	3411.702	3353.565	5374.588	824.875
control	Sb	22	0.000	1.603	1.035	4.233	1.382
suicide	Sb	13	0.000	0.932	0.526	4.234	1.116
control	Se	22	209.628	441.216	451.592	684.611	117.952
suicide	Se	13	257.576	442.796	467.472	598.394	96.647
control	Se2	22	191.601	396.726	411.489	597.100	102.306
suicide	Se2	13	253.552	403.325	433.004	535.463	79.936
control	Sm	22	0.020	0.116	0.082	0.571	0.125
suicide	Sm	13	0.008	0.189	0.156	0.773	0.193
control	Sn	22	0.777	27.242	12.433	257.239	54.303

suicide	Sn	13	0.673	34.201	12.630	167.177	47.378
control	Sr	22	41.591	244.076	199.246	883.603	192.085
suicide	Sr	13	54.467	230.614	141.464	614.655	189.183
control	Tb	22	0.003	0.028	0.019	0.153	0.032
suicide	Tb	13	0.000	0.045	0.040	0.098	0.029
control	Th	22	0.000	0.000	0.000	0.000	0.000
suicide	Th	13	0.000	0.000	0.000	0.000	0.000
control	Ti	22	12.465	27.619	27.154	46.892	8.243
suicide	Ti	13	15.747	27.704	29.183	35.244	6.062
control	Tl	22	0.000	0.074	0.000	0.439	0.124
suicide	Tl	13	0.000	0.046	0.033	0.126	0.050
control	Tm	22	0.000	0.008	0.008	0.017	0.005
suicide	Tm	13	0.000	0.010	0.007	0.026	0.008
control	U	22	0.000	0.107	0.076	0.566	0.133
suicide	U	13	0.000	0.060	0.010	0.345	0.099
control	V	22	0.847	4.409	2.774	25.196	5.151
suicide	V	13	1.186	4.355	3.535	11.053	2.928
control	Yb	22	0.000	0.038	0.030	0.151	0.036
suicide	Yb	13	0.000	0.054	0.017	0.313	0.082
control	Zn	22	23125.740	65063.161	53762.031	206078.032	41502.915
suicide	Zn	13	21777.193	75351.381	69178.928	175316.419	44475.388
control	Zr	22	0.000	1.413	1.127	6.163	1.302

suicide	Zr	13	0.000	2.613	1.112	20.617	5.450

XII.liver (L)

Element	Control mean	Suicide mean	Dunn statistic	p
Ag	15.909	21.538	1.573	0.116
Al	17.591	18.692	0.307	0.759
As	18.523	17.115	−0.422	0.673
As2	18.182	17.692	−0.157	0.875
Ba	18.364	17.385	−0.273	0.785
Be	17.409	19.000	0.539	0.590
Bi	19.318	15.769	−0.991	0.321
Ca	17.364	19.077	0.478	0.633
Cd	16.773	20.077	0.922	0.357
Ce	15.227	22.692	2.083	0.037
Co	18.909	16.462	−0.683	0.495
Cr	16.909	19.846	0.823	0.411
Cs	17.591	18.692	0.307	0.759
Cu	16.500	20.538	1.127	0.260
Dy	16.227	21.000	1.331	0.183
Er	16.545	20.462	1.095	0.273
Eu	14.500	23.923	2.629	0.009
Fe	15.636	22.000	1.775	0.076
Ga	15.682	21.923	1.748	0.080
Gd	15.091	22.923	2.185	0.029

Hf	16.568	20.423	1.192	0.233
Hg	17.909	18.154	0.068	0.946
Hg2	17.909	18.154	0.068	0.946
Ho	18.000	18.000	0.000	1.000
K	16.500	20.538	1.127	0.260
La	15.182	22.769	2.117	0.034
Mg	15.318	22.538	2.014	0.044
Mn	16.591	20.385	1.058	0.290
Mo	17.091	19.538	0.683	0.495
Na	16.182	21.077	1.366	0.172
Nd	15.091	22.923	2.185	0.029
Ni	18.091	17.846	−0.068	0.946
P	16.545	20.462	1.092	0.275
Pb	18.273	17.538	−0.205	0.838
Pd	19.341	15.731	−1.010	0.313
Pr	15.045	23.000	2.219	0.026
Pt	17.000	19.692	0.752	0.452
Rb	15.773	21.769	1.673	0.094
Sb	19.682	15.154	−1.264	0.206
Se	17.909	18.154	0.068	0.946
Se2	17.682	18.538	0.239	0.811
Sm	15.636	22.000	1.775	0.076
Sn	17.909	18.154	0.068	0.946
Sr	18.545	17.077	−0.410	0.682
Tb	14.955	23.154	2.287	0.022

Ti	17.636	18.615	0.273	0.785
Tl	17.636	18.615	0.303	0.762
Tm	17.682	18.538	0.239	0.811
U	19.773	15.000	−1.352	0.176
V	17.000	19.692	0.751	0.453
Yb	18.114	17.808	−0.085	0.932
Zn	16.909	19.846	0.819	0.413
Zr	18.068	17.885	−0.051	0.959
